# Integrative multi-omics reveals energy metabolism–related prognostic signatures and immunogenetic landscapes in lung adenocarcinoma

**DOI:** 10.3389/fimmu.2025.1679464

**Published:** 2025-10-14

**Authors:** Lei Xie, Yajie Zhou, Zijian Hu, Wenxiong Zhang, Xiaoqiang Zhang

**Affiliations:** ^1^ Department of Thoracic Surgery, The Second Affiliated Hospital, Jiangxi Medical College, Nanchang University, Nanchang, China; ^2^ Lung Cancer Center, The Second Affiliated Hospital, Jiangxi Medical College, Nanchang University, Nanchang, China

**Keywords:** energy metabolism, lung adenocarcinoma, prognostic model, machine learning, multi-omics

## Abstract

**Background:**

Energy metabolism (EM) is critically involved in driving tumor development, therapeutic resistance, and modulation of the immune response. However, its genetic basis and prognostic value in lung adenocarcinoma (LUAD) remain unclear. This study integrates multi-omics approaches to develop an EM-related prognostic model for assessing LUAD prognosis and uncovering relevant immunogenetic pathways.

**Methods:**

Differential analysis combined with Mendelian randomization was used to identify EM-related genes (EMRGs) with a causal link to LUAD, which were then used to build a prognostic model via machine learning. Nomogram integrating clinical features and risk model was developed to enhance prognostic accuracy. Subsequent analyses, including immune invasion, enrichment analysis, and tumor mutational burden (TMB), were conducted to explore biological associations. The heterogeneity and cell-specific expression of critical EMRGs were explored through single-cell RNA sequencing (scRNA-seq). The transcriptional levels of the chosen EMRGs were experimentally validated using reverse transcription quantitative PCR (RT-qPCR).

**Results:**

A prognostic model was established in our study using Random Survival Forest (RSF) machine learning (ML) algorithm. Survival outcomes were substantially lower in the high-risk group (HRG) than in the low-risk group (LRG), as reflected by an AUC value of 0.73. A nomogram incorporating this risk model outperformed one without it. Gene Ontology (GO)/Kyoto Encyclopedia of Genes and Genomes (KEGG)-based analyses showed a significant enrichment of these genes in pathways linked to immune regulation and extracellular matrix (ECM) dynamics. An elevated TMB in HRG may predict a worse prognosis. Evaluation of pharmacologic susceptibility revealed enhanced drug sensitivity in the HRG, such as Cytotoxic Chemotherapy and Apoptosis-inducing small molecule inhibitors, etc. ScRNA-seq revealed that prognostic EMRGs were mainly enriched in T and NK cells, myeloid cells, and fibroblasts, suggesting their involvement in immune regulation and remodeling of the tumor microenvironment (TME). RT-qPCR confirmed their differential expression in LUAD and normal cell lines.

**Conclusions:**

This integrative model reveals the prognostic and therapeutic relevance of EMRGs in LUAD, presenting a novel structure for immunogenetic risk assessment and personalized treatment strategies.

## Introduction

The incidence of lung cancer is steadily increasing worldwide, making it the leading cause of cancer-related mortality, particularly in younger populations (1). Lung adenocarcinoma (LUAD), which makes up more than half of the patients, is the most commonly occurring form of non-small cell lung cancer (NSCLC) (2, 3). In LUAD treatment, immune checkpoint inhibitors (ICIs), have proven to be effective, yet only a few patients benefit from prolonged clinical improvement (4–6). Identifying novel molecular biomarkers is crucial for improving prognostic classification and facilitating personalized therapy in LUAD (7).

Energy metabolism (EM) alterations are now commonly acknowledged as a hallmark of cancer. Cells in LUAD frequently favor aerobic glycolysis over oxidative phosphorylation to satisfy their bioenergetic and biosynthetic needs—a phenomenon called the Warburg effect (8). Beyond glycolysis, aberrant regulation of other metabolic pathways, such as fatty acid oxidation, glutaminolysis, and mitochondrial function, also contributes to tumor progression, immune evasion, and therapy resistance (9, 10). Tumor EM has been increasingly recognized as a key regulator of the tumor microenvironment (TME), affecting both immune landscape and stromal remodeling, thereby influencing treatment outcomes (11, 12).

However, the prognostic significance and genetic underpinnings of energy metabolism-related genes (EMRGs) in LUAD remain insufficiently characterized. To address this gap, we built a robust prognostic model based on EMRGs by integrating multi-omics and multiple machine learning (ML) algorithms. Using genetic variants as instrumental variables, Mendelian randomization (MR) infers causal relationships between gene expression and clinical outcomes, minimizing biases like confounding and reverse causality (13). Complementarily, ML algorithms offer high-dimensional feature selection and predictive modeling capabilities, enabling the identification of gene signatures with superior prognostic accuracy (14). And single-cell RNA sequencing (scRNA-seq) was utilized to analyze the cellular localization and heterogeneity of key EMRGs, providing a high-resolution view of their functional roles within the TME (15).

## Materials and methods

### Data acquisition

LUAD data of survey are primarily derived from The Cancer Genome Atlas (TCGA) database (https://portal.gdc.cancer.gov/, till March 1st, 2025). In addition, three external validation cohorts (GSE30219, GSE72094 and GSE50081) and a single-cell sequencing cohort (GSE189357) were download the Gene Expression Omnibus (GEO) repository (https://www.ncbi.nlm.nih.gov/geo/, accessible until March 1st, 2025). Initially, 9950 EMRGs were derived from GeneCards database (https://www.genecards.org/, on March 1st, 2025) using “EM” as the search term and a relevance score exceeding 1. Expression profiles of these genes in TCGA-LUAD samples were then extracted via the “limma” R package. Differentially expressed genes (DEGs) were screened based on criteria of |log2 fold change| greater than 1 and a false discovery rate (FDR) less than 0.05 for further analysis.

For MR analysis, summary statistics for LUAD and a blood Expression Quantitative Trait Loci (eQTL) data from eQTLGen consortium were retrieved via the IEU GWAS platform (https://gwas.mrcieu.ac.uk/). The eQTL data encompassed associations for 19,942 genes derived from 31,684 blood samples of healthy individuals of European descent (16). In parallel, GWAS summary data on LUAD (11,273 cases and 55,483 controls; study ID: “ebi-a-GCST004744”) were obtained, comprising 7,849,324 single nucleotide polymorphisms (SNPs) from a European-ancestry cohort.

### MR statistical analysis and evaluation

To generate eQTL instruments for individual genes, we selected SNPs with strong associations (P < 5×10^-8^) from GWAS-derived summary eQTL datasets as instrumental variables (IVs). To minimize confounding due to high linkage disequilibrium (LD), SNPs exhibiting r² > 0.001 within a 10 Mb window were excluded in favor of the most strongly associated variant. The F-statistic was used to quantify instrument strength, calculated as F = R² × (N-2)/(1-R²) (N: the sample size; R²: the proportion of variance in the exposure explained by the SNP) (17).

We utilized the “TwoSampleMR” R package to perform five MR approaches for evaluating the potential causal association between eQTLs and LUAD. In order to strengthen the credibility of our findings, we applied sensitivity analyses, with Cochran’s Q test being one of the key methods used, funnel plot visualization, leave-one-out diagnostics, and the MR-Egger intercept method. Heterogeneity among SNP instruments was examined via Cochran’s Q statistic (P-value less than 0.05 indicated significant heterogeneity). Funnel plots were employed to detect asymmetry suggestive of bias. Leave-one-out analysis was implemented to determine whether any single SNP disproportionately influenced the outcome. Additionally, the presence of horizontal pleiotropy was assessed using the MR-Egger intercept; a significant intercept (*P* < 0.05) implied pleiotropy, which would violate core MR assumptions and lead to exclusion of that result (18).

### Signature constructed through machine learning–driven integrative method

To achieve a robust and reliable consensus on prognostic EMRGs, we implemented a comprehensive strategy that integrated ten machine learning techniques across 117 unique algorithmic combinations. Our approach leveraged ML algorithms, including Random Survival Forest (RSF), Supervised Principal Components, Generalized Boosted Regression Modeling (GBM), Partial Least Squares for Cox regression, Stepwise Cox, CoxBoost, Survival Support Vector Machine, Ridge, Elastic Net, and Least Absolute Shrinkage and Selection Operator (LASSO) (19).The workflow for constructing the prognostic model consisted of the following steps: (a) DEGs that overlapped with the MR-identified candidates were selected to define LUAD-relevant EMRGs; (b) The 117 combinations of algorithms were evaluated via Leave-One-Out Cross-Validation (LOOCV), utilizing the intersected gene set as input for model construction; (c) Each model’s performance was externally validated using three GEO cohorts (GSE30219, GSE72094, and GSE50081); (d) The models were evaluated using the concordance index (C-index) across all datasets, and the model with the highest mean C-index was considered the optimal one (20).

### Validation model and nomogram

With the optimal ML algorithm, we estimated risk scores from the prognostic gene panel and stratified patients into high, low risk group (HRG&LRG) according median values (21). Our study plotted Kaplan-Meier (K-M) survival curves using R packages “survival”, “survminer” to assess overall survival (OS) in different groups. In addition, we created scatterplots of risk distributions, Principal component analysis (PCA), and gene expression heat maps to observe differences between HRG&LRG. Subsequently, we plotted K-M curves for various clinical features to investigate their feasibility in the model.

To determine whether EMRGs serve as independent prognostic indicators in LUAD patients, Cox regression analyses, including both univariate and multivariate models, were performed (22), evaluating their association with clinical variables. To generate the prognostic nomogram, TCGA-LUAD cohort data were analyzed using the “rms”, “replot” packages in R. To assess the clinical utility and accuracy of the nomogram, we performed decision curve analysis (DCA), receiver operating characteristic (ROC) curve analysis, and calibration plots.

### Biological function and pathway enrichment analysis

DEGs between the HRG&LRG were identified and annotated using “clusterProfiler”, “https://bioconductor.org/packages/release/data/annotation/html/org.Hs.eg.db.html” R packages (23). To delineate pathway differences between HRG&LRG, we applied Gene Ontology (GO), Kyoto Encyclopedia of Genes and Genomes (KEGG) for in-depth analyses.

### Tumor mutation burden

Using R package “maftools”, we computed the TMB score for each patient and created waterfall plots to visualize the mutation data for the TCGA-LUAD cohort (24). In addition, we performed survival curve analysis to distinguish differences in OS between groups of patients classified according to different TMB levels (25).

### Tumor microenvironment analysis

Differences in ESTIMATE, immune, and stromal composite scores between HRG&LRG were calculated using the ‘ESTIMATE’ R tool. Furthermore, immune cell infiltration levels were evaluated using multiple computational tools, including XCELL, TIMER, QUANTISEQ, and MCPCOUNTER, to enhance the robustness of the immune profiling (26). We performed immunoinfiltration analysis on multiple immune cell lines to estimate the abundance of different immune cell subsets. To examine immune functional states in LUAD population, we performed single-sample gene set enrichment analysis, which formed the basis for differential analysis between HRG & LRG.

### Immunotherapy and chemotherapy effectiveness in LUAD

To explore the variance in rejection reactions, immune dysfunction between HRG&LRG, we employed Tumor Immune Dysfunction and Exclusion (TIDE) website (http://tide.dfci.harvard.edu/) to calculate TIDE scores (27). We compared the TIDE scores between the two groups to assess differences in immunotherapy responses. Using the “oncoPredict” R package integrated with Genomics of Drug Sensitivity in Cancer (GDSC) datasets, we estimated the half-maximal inhibitory concentration (IC50) values of multiple anticancer agents (28). Drug sensitivity differences between HRG&LRG were then evaluated by comparing these IC50 estimates.

### Single-cell sequencing analysis of EMRGs

The scRNA-seq dataset GSE189357, comprising nine LUAD samples, was retrieved from GEO database. Using “Seurat” R package, the data were transformed into Seurat objects. We excluded cells with less than 500 genes and genes present in fewer than three cells. Mitochondrial gene percentages were computed via the PercentageFeatureSet function, and violin plots were generated with VlnPlot to assess gene expression distributions. Quality control involved filtering out outliers based on gene count ranges and transcript abundance, excluding cells with over 25% mitochondrial content (29). PCA was employed for initial dimensionality reduction, while batch effects were mitigated using Harmony, which iteratively determined optimal reduction thresholds. The visualization of cell clusters was performed using Uniform Manifold Approximation and Projection (UMAP) (30). Cellular distances were estimated using FindNeighbors, and clusters were defined using the FindClusters function with a specified resolution parameter. Using reference transcriptomic datasets, cell-type annotation was performed with the “SingleR” package.

### CeRNA regulatory network

To identify potential miRNA targets, we utilized TargetScan Human (https://www.targetscan.org), miRWalk (http://mirwalk.umm.uni-heidelberg.de/). Predictions of miRNA-lncRNA interactions were performed using SpongeScan (https://www.repository.cam.ac.uk/). The regulatory network comprising lncRNA, miRNA, and mRNA was assembled and displayed via Cytoscape software (31).

### Verify protein and mRNA expression of EMRGs

The protein expression profiles of EMRGs in tumor versus normal cell lines were downloaded from Human Protein Atlas database (https://www.proteinatlas.org/). Furthermore, we obtained HBE (a human lung fibroblast cell line), PC-9, A549 and H1299 (three human lung adenocarcinoma cell lines) supplied by Procell life science &technology co. ltd. Subsequently, we used the TRIzol Reagent and the Prime Script RT Kit (obtained from VWR International) to extract the RNA from these cell lines, and reverse transcribed it. Finally, we analyzed their gene expression by RT-qPCR (32). **Supplementary Table S1** shows the primers for EMRGs.

## Results

### Causal relationship between EMRGs and LUAD

**Figure 1:**
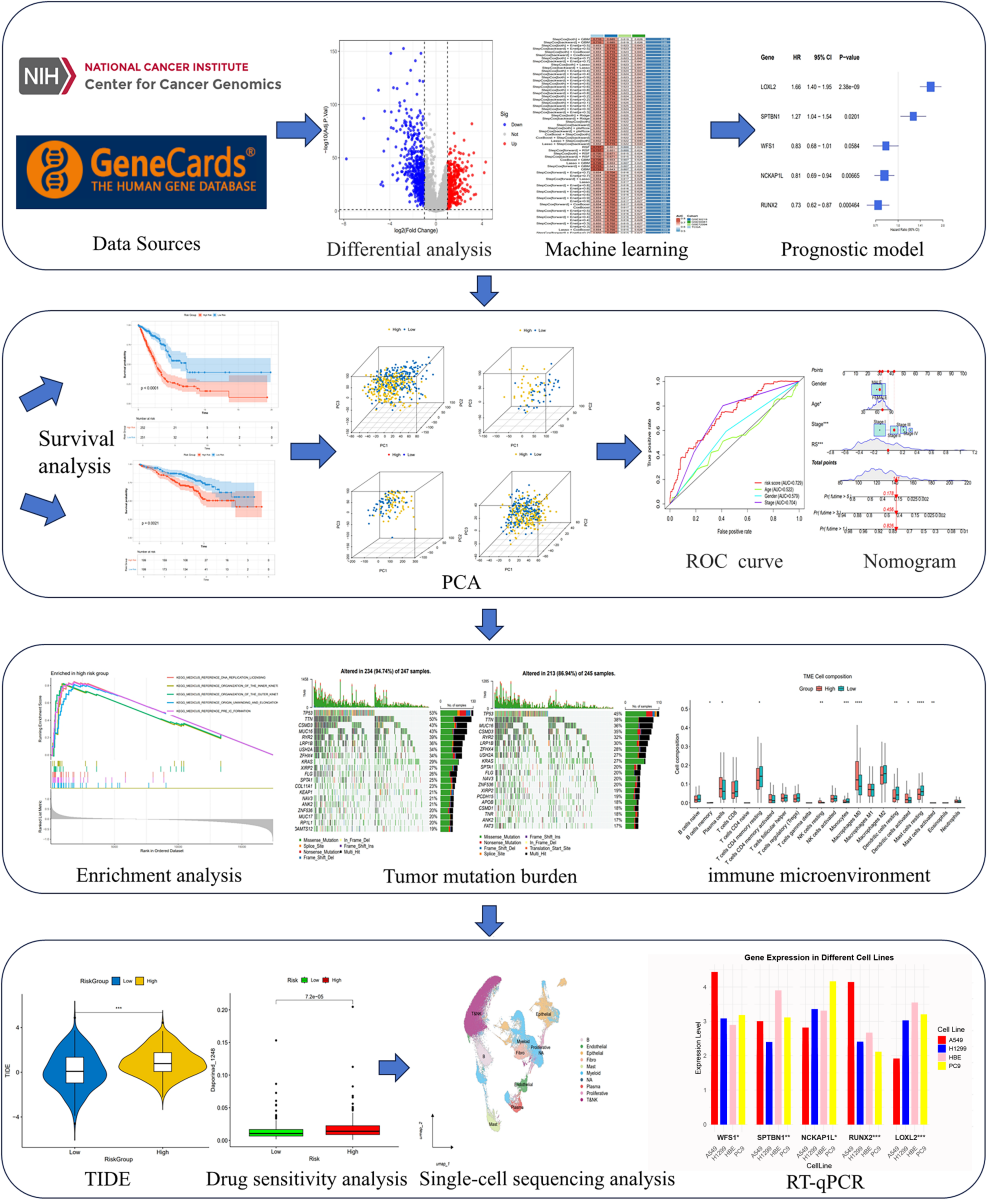
Flow chart of the study design. An overview of the analytical workflow including data collection, Mendelian randomization, machine learning-based prognostic modeling, validation, and downstream tumor microenvironment and therapeutic analyses.

**Figure 2:**
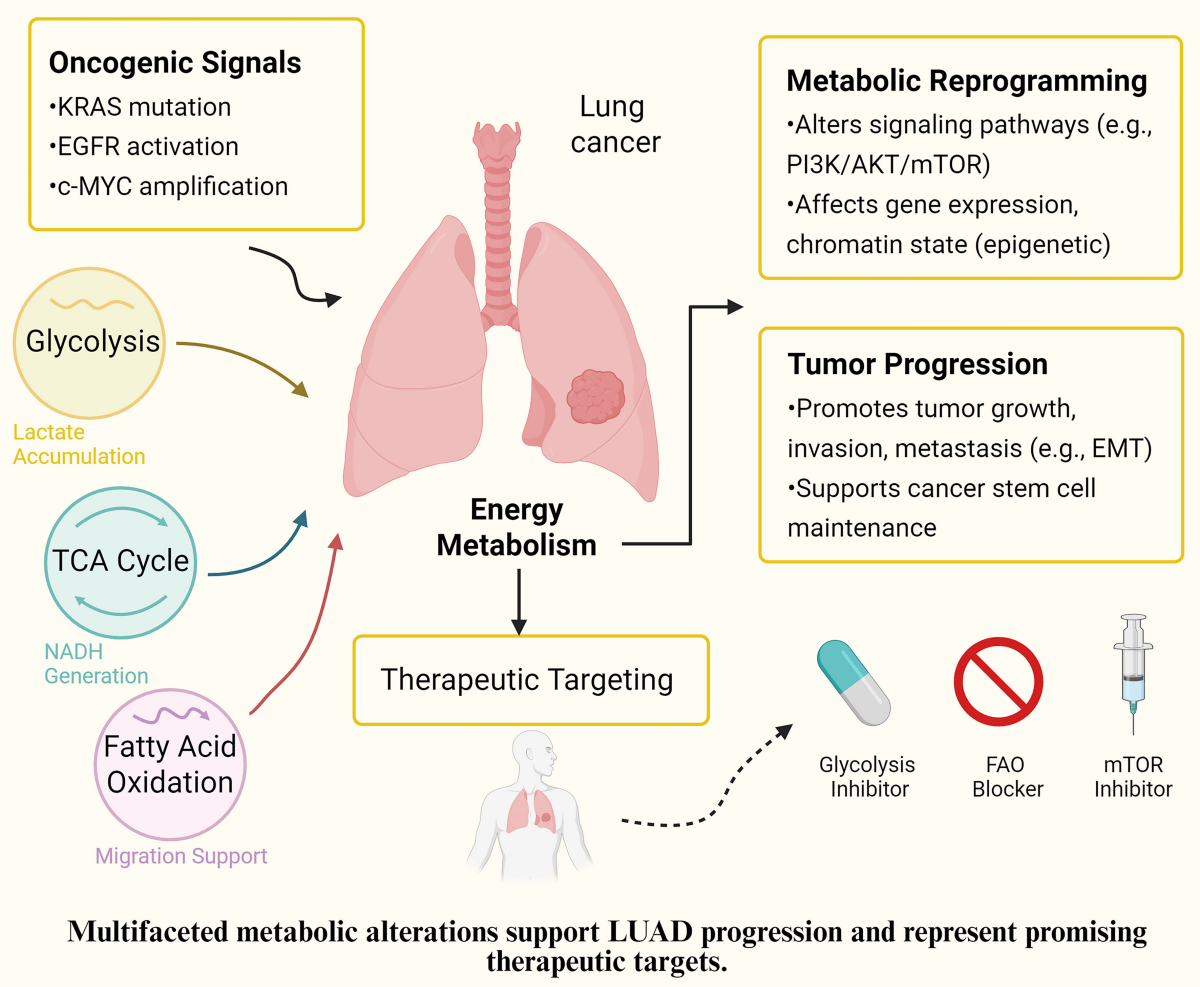
Proposed mechanism of EMRGs in LUAD. Schematic representation of how the identified energy metabolism-related genes (EMRGs: WFS1, SPTBN1, NCKAP1L, RUNX2, and LOXL2) regulate glycolysis, extracellular matrix remodeling, and m^6A methylation, thereby shaping the tumor microenvironment (immune cell infiltration, fibroblast activation, and myeloid-derived suppressor cells) and ultimately influencing prognosis and therapeutic responses in LUAD.

**Figure 3:**
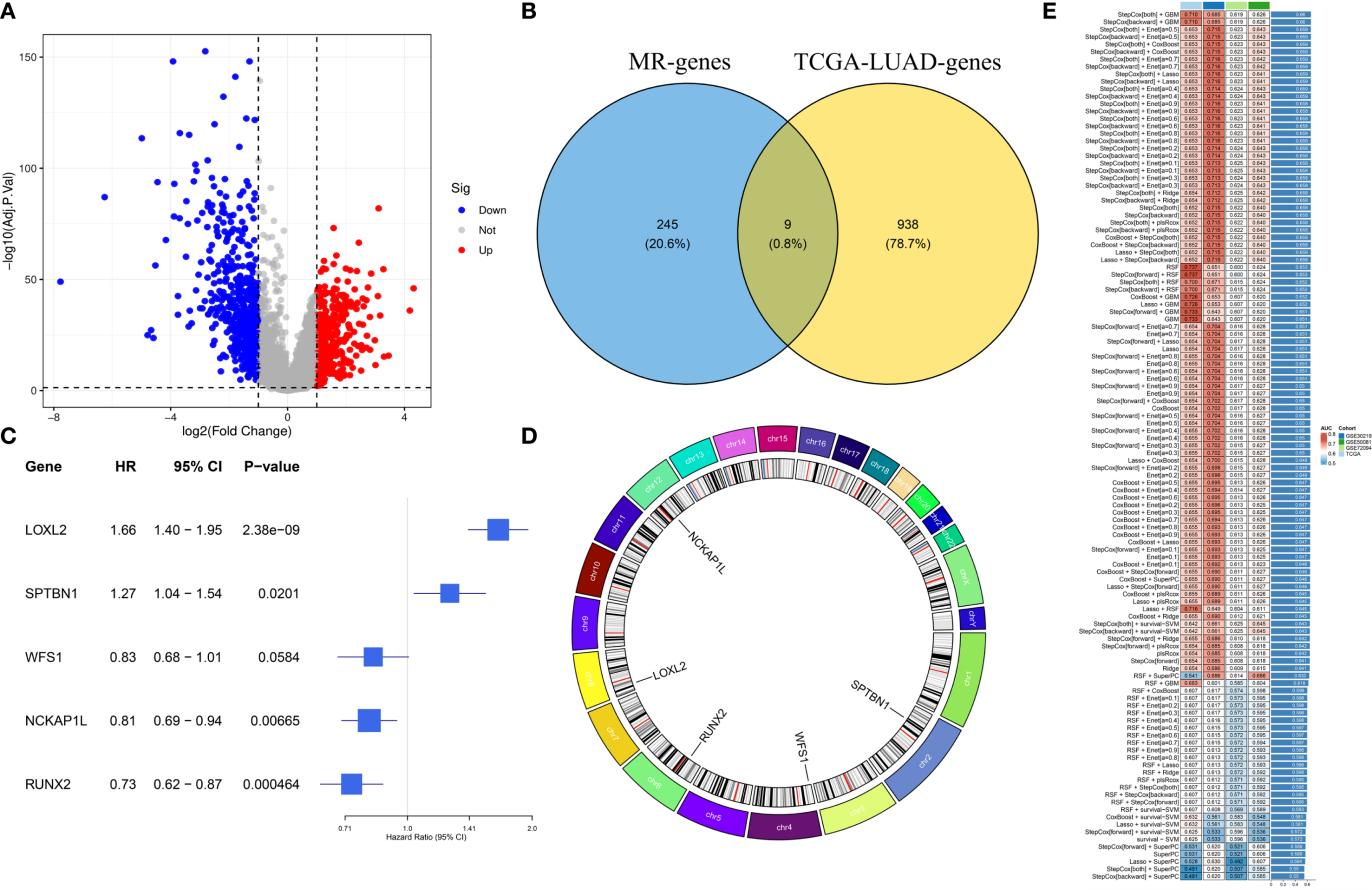
Identification of prognostic EMRGs. **(A)** Volcano plot of differentially expressed EMRGs. **(B)** Venn diagram integrating Mendelian randomization and differential expression analyses. **(C)** Machine learning selection of 5 EMRGs associated with prognosis. **(D)** Circos plot showing chromosomal locations and expression levels of the model genes. **(E)** Model performance: the C-index of 117 predictive models generated by ten-fold cross-validation, evaluated across TCGA and GEO datasets.

We have drawn a specific technology roadmap for this article (**Figure 1**). The specific mechanism of EM is shown in **Figure 2**. Through MR analysis, we found 245 gene symbols causally associated with LUAD. All IVs showed F-statistics > 10, and the findings were consistent across five different MR methods, suggesting robust causal estimates (**Supplementary Table S2**). We obtained the LUAD data from TCGA database, including 503 oncology patients and 59 normal patients (**Table 1**). A total of 9,950 EMRGs were initially identified in the TCGA-LUAD dataset, from which 947 DEGs were filtered (**Figure 3A**). By intersecting these with the MR results, 9 overlapping genes were obtained (**Supplementary Table S3**) (**Figure 3B**). Furthermore, both Cochran’s Q test and Egger’s intercept analysis revealed no significant evidence of heterogeneity or horizontal pleiotropy (**Supplementary Table S4**). Forest and scatter plots also indicated minimal variability contributed by individual SNPs (**Supplementary Figures S1**, **S2**).

**Table 1:** Clinical information of the patients in the TCGA and GEO groups.

Characteristics	TCGA(n=503)		GSE30219(n=85)		GSE72094(n=442)		GSE50081(n=127)	
	n	%	n	%	n	%	n	%
Age								
<=65	238	47.32	60	70.59	127	28.73	42	33.07
>65	255	50.70	25	29.41	294	66.52	85	66.93
Unknow	10	1.99	–	–	21	4.75	–	–
Status								
Alive	321	63.82	40	47.06	298	67.42	76	59.84
Dead	182	36.18	45	52.94	122	27.60	51	40.16
Unknow	–	–	–	–	22	4.98	–	–
Gender								
Female	272	54.08	19	22.35	240	54.30	62	48.82
Male	231	45.92	66	77.65	202	45.70	65	51.18
Stage								
StageI	270	53.68	–	–	265	59.95	92	72.44
StageII	120	23.86	–	–	69	15.61	35	27.56
StageIII	80	15.90	–	–	63	14.25	–	–
StageIV	25	4.97	–	–	17	3.86	–	–
Unknow	8	1.59	–	–	28	6.33	–	–
T stage								
T1	168	33.40	71	83.53	–	–	43	33.86
T2	269	53.48	12	14.12	–	–	82	64.57
T3	45	8.95	2	2.35	–	–	2	1.57
T4	18	3.58	–	–	–	–	–	–
Unknow	3	0.60	–		–	–	–	–
M stage								
M0	335	66.60	85	100	–	–	127	100
M1	24	4.77	–	–	–	–	–	–
Unknow	144	28.63	–	–	–	–	–	–
N stage								
N0	326	64.81	82	96.47	–	–	94	74.02
N1	95	18.89	3	3.53	–	–	33	25.98
N2	69	13.72	–	–	–	–	–	–
N3	2	0.40	–	–	–	–	–	–
Unknow	11	2.19	–	–	–	–	–	–

GEO, Gene Expression Omnibus; TCGA, The Cancer Genome Atlas; T stage, Tumor stage; N stage, Node stage; M stage, metastasis stage.

### Establish and validate the risk model

We developed a prognostic model using a machine learning-based integrative approach and expression profiles of 46 DEGs. Using the TCGA-LUAD and GEO datasets, Through LOOCV, 117 predictive models were systematically evaluated, and their C-index scores were subsequently calculated (**Figure 3E**). Notably, the stepCOX [both] + GBM combination achieved the highest C-index. As a result, the algorithm was used for feature selection and model construction, culminating in the identification of the five most influential genes (**Figure 3C**). The chromosomal position of each gene is shown in **Figure 3D**. The GBM model was applied to generate risk estimates for each LUAD case, which were then split into HRG&LRG according to the median threshold. **Table 2** summarizes the clinical information for the HRG&LRG populations. KM curves revealed significantly longer OS in the LRG versus the HRG across all cohorts (TCGA: P<0.0001; GSE30219: P = 0.0077; GSE72094: P<0.0021; GSE50081: P = 0.018). Consistently, time-dependent ROC analysis demonstrated strong predictive performance, with 1-, 3-, 5-year AUCs of 0.736/0.760/0.737 (TCGA), 0.64/0.735/0.740 (GSE30219), 0.618/0.656/0.679 (GSE72094), and 0.704/0.684/0.680 (GSE50081) (**Supplementary Figures S3A–H**). Scatter plots of risk scores and gene expression heatmaps further distinguished HRG&LRG across cohorts (**Supplementary Figures S4A–D**).

**Table 2:** Clinical information for 503 TCGA patients in different risk categories.

Characteristics	High-risk group (n=251)		Low-risk group (n=252)	
	n	%	n	%
Age				
<=65	128	51.00	110	43.65
>65	118	47.01	137	54.37
Unknow	5	1.99	5	1.98
Status				
Alive	122	48.61	199	78.97
Dead	129	51.39	53	21.03
Gender				
Female	125	49.80	147	58.33
Male	126	50.20	105	41.67
Stage				
Stage I	109	43.43	161	63.89
Stage II	72	28.69	48	19.05
Stage III	53	21.12	27	10.71
Stage IV	15	5.98	10	3.97
Unknow	2	0.80	6	2.38
T stage				
T1	71	28.29	97	38.49
T2	135	53.78	134	53.17
T3	30	11.95	15	5.95
T4	13	5.18	5	1.98
Unknow	2	0.80	1	0.40
M stage				
M0	176	70.12	159	63.10
M1	14	5.58	10	3.97
Unknow	61	24.30	83	32.94
N stage				
N0	142	56.57	184	73.02
N1	61	24.30	34	13.49
N2	45	17.93	24	9.52
N3	–	–	2	0.79
Unknow	3	1.20	8	3.17

TCGA, The Cancer Genome Atlas**;** T stage, Tumor stage; N stage, Node stage; M stage, metastasis stage.

### Prediction nomogram for this model

**Figure 4:**
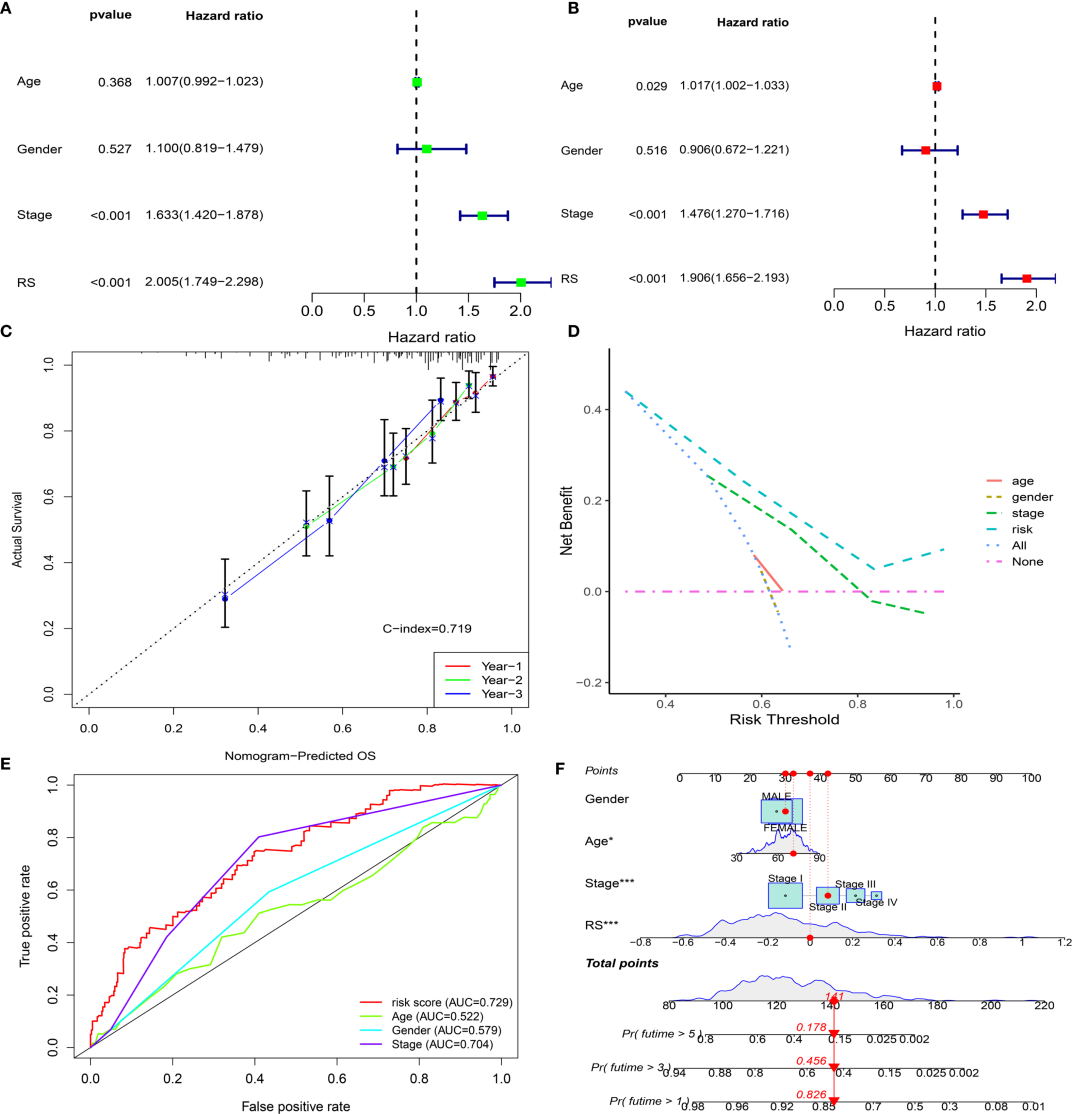
Development and validation of the EMRG prognostic signature. **(A, B)** Univariate and multivariate Cox analyses of clinical characteristics and risk scores in the TCGA cohort. **(C)** Calibration plot for predicting 1-, 2-, and 3-year overall survival. **(D)** Decision curve analysis (DCA) for 5-year survival. **(E)** ROC curves comparing clinical factors with the EMRG signature. **(F)** Nomogram integrating the EMRG signature and clinical features to predict patient prognosis.

The prognostic independence of EMRGs was examined using univariate and multivariate Cox regression methods. Univariate results showed that EMRGs were obviously associated with survival risk (HR= 2.005, 95% CI = 1.749–2.298, P< 0.001) (**Figure 4A**). Even after adjusting for potential confounding variables, multivariate analysis affirmed EMRGs as independent prognostic indicators (HR = 1.906, 95% CI = 1.656–2.193, P< 0.001) (**Figure 4B**). Drawing on the multivariate Cox analysis, we designed a nomogram to predict OS at 1, 3, and 5 years in LUAD (**Figure 4F**). Our nomogram’s accuracy was assessed through calibration curve analysis (**Figure 4C**), while ROC curves and DCA confirmed the model’s enhanced predictive capability over traditional clinical parameters (**Figures 4D, E**). Scatter plots illustrate associations between clinical traits and risk scores (**Supplementary Figures S5A–D**). A heatmap integrates the expression patterns of 5 EMRGs with clinical data and risk groups (**Supplementary Figure S5E**). Stratified survival analysis across clinical subgroups consistently supported the model’s predictive value (**Supplementary Figures S6A–K**).

### KEGG and GO enrichment analysis

Enrichment analyses of EMRGs were conducted across HRG& LRG cohorts to explore their functional disparities. KEGG pathway analysis revealed that top six obviously enriched pathways were mainly associated with oncogenesis, immune modulation, and DNA damage repair processes. These included DNA replication licensing, pre-initiation complex assembly, outer and inner kinetochore organization, TRAIP-mediated replisome disassembly, and replication origin unwinding and elongation (**Supplementary Figure S7A**) (**Supplementary Table S5**). Meanwhile, GO enrichment of biological processes highlighted several immune and structural remodeling functions, such as tissue repair, regulation of body fluid dynamics, enhancement of cell adhesion, and organization of the extracellular matrix and encapsulating structures (**Supplementary Figure S7B**) (**Supplementary Table S6**).

### Tumor mutational burden

**Figure 5:**
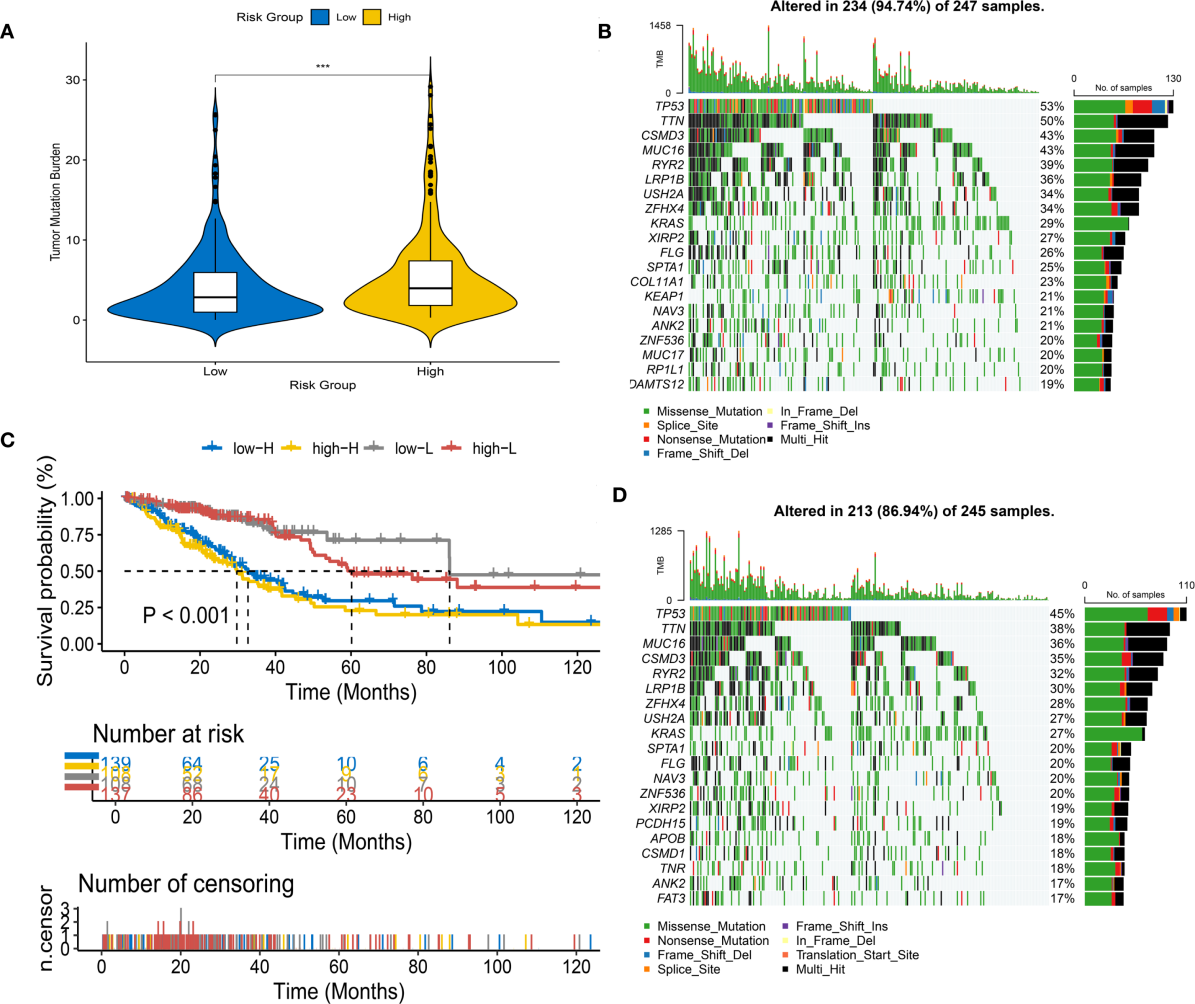
Tumor mutational burden (TMB) analysis in different risk groups. **(A)** Violin plot of TMB levels in high-risk (HRG) and low-risk groups (LRG). **(B)** Survival analysis stratified by risk score and mutation frequency. **(C, D)** Waterfall plots of mutation profiles in LRG **(C)** and HRG **(D)**.

We calculated the TMB values for each patient. We found significant differences in TMB between HRG&LRG (P<0.001). We further stratified patients into HRG&LRG, revealing a highly significant survival disparity between the two groups (P < 0.001) (**Figures 5A, B**). We developed waterfall plots based on different subclusters, including the top 20 mutant genes, depicting a mutation landscape of the ten most prevalent genes in the two risk groups (**Figures 5C, D**).

### Immune microenvironment analysis

**Figure 6:**
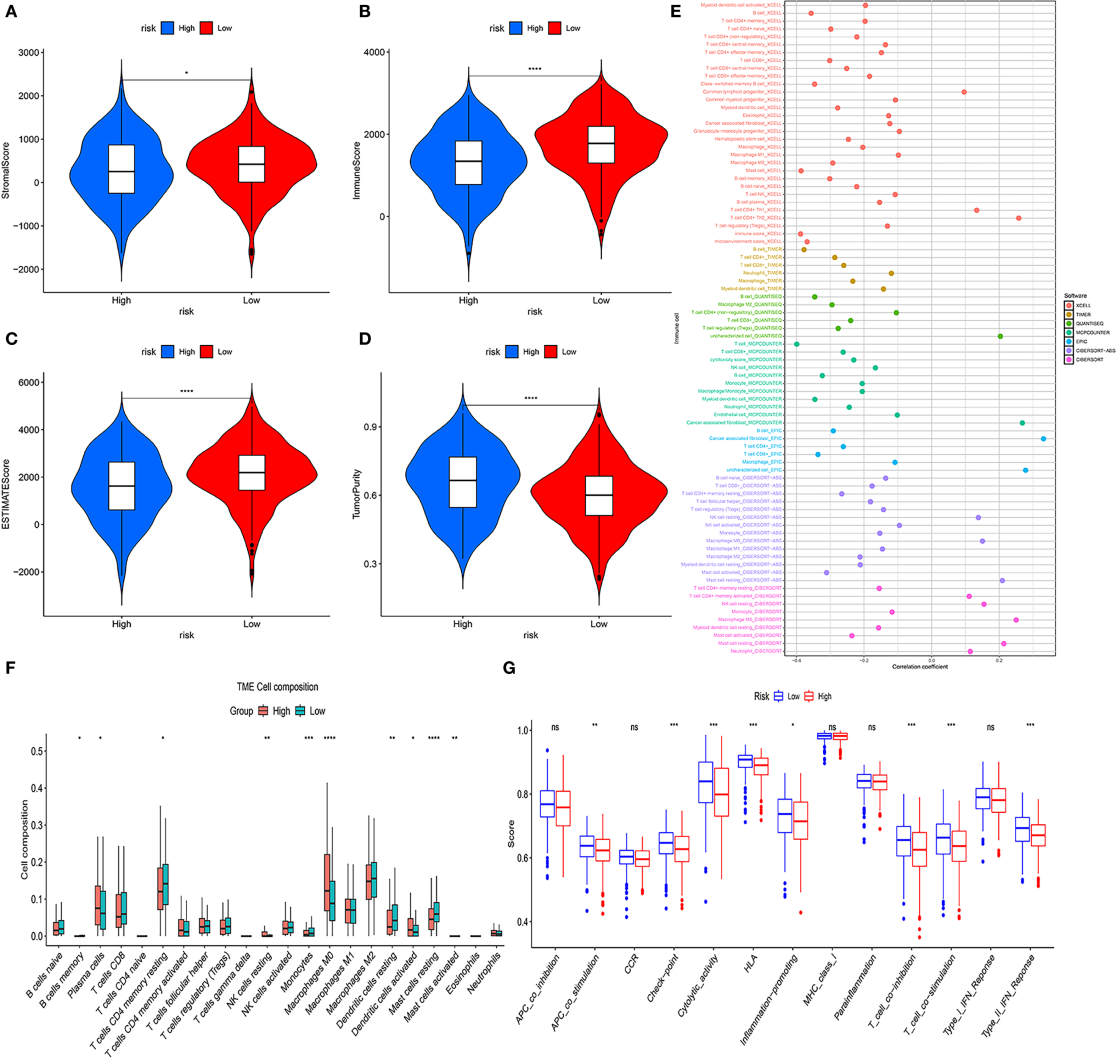
Tumor microenvironment (TME) analysis of risk subgroups. **(A–D)** Violin plots showing stromal scores, immune scores, ESTIMATE scores, and tumor purity. **(E)** Bubble plots illustrating correlations between risk scores and immune cell abundance under six algorithms. **(F)** CIBERSORT analysis of proportions of 22 immune cells in the two subgroups. **(G)** ssGSEA immune function score comparison between HRG and LRG (ns, not significant, *P<0.05, **P<0.01, ***P<0.001, ****P < 0.0001).

TME analysis revealed notable immune cell distribution differences between the risk groups. Specifically, the HRG showed significantly higher infiltration of M0 macrophages (P < 0.0001), neutrophils (P < 0.001), and activated mast cells (P < 0.05). In contrast, the LRG had elevated levels of B lymphocytes (P < 0.0001), resting memory CD4+ T cells (P < 0.001), monocytes (P < 0.05), plasma cells (P < 0.05), and resting mast cells (P < 0.05) (**Figure 6F**). Furthermore, among 13 immune-related functional signatures, the HRG displayed significantly reduced activity in checkpoint pathways (P < 0.001), cytolytic processes (P < 0.01), HLA molecule expression (P < 0.001), inflammation-promoting signals (P < 0.01), as well as both T cell co-inhibitory and co-stimulatory pathways (P < 0.001), type II interferon responses (P < 0.001) (**Figure 6G**). Complementary analysis revealed that the LRG exhibited notably elevated immune, stromal, and ESTIMATE scores (immune score: P < 0.0001; stromal score: P < 0.05; ESTIMATE scores: P < 0.0001), while the HRG exhibited significantly higher tumor purity than the LRG (P < 0.001) (**Figures 6A–D**). These findings were further corroborated by various deconvolution algorithms that identified robust associations between immune cell infiltration and risk stratification (**Figure 6E**), with extended results visualized in **Supplementary Figure S8**.

### Immune targets and chemotherapy

TIDE score analysis showed that in the HRG characterized by EM-related features, the T cell dysfunction score (**Supplementary Figure S9A**) was lower than those in the LRG, while the TIDE score (**Supplementary Figure S9B**) and T cell exclusion score (**Supplementary Figure S9C**) were higher. These results indicate that tumors in the HRG may have higher levels of T cell infiltration accompanied by greater functional impairment, potentially contributing to enhanced immune evasion. Furthermore, we calculated the IC50 of antitumor drugs for both HRG&LRG patients. We screened 89 chemotherapeutic agents and identified substantial differences in predicted IC50 values between HRG & LRG (**Supplementary Table S7**). The HRG appears to exhibit greater sensitivity to certain drugs, such as BI.2536 and BMS.536924. Whereas the LRG is sensitive to Docetaxel_1007 and Topotecan. The IC50 values of these drugs can help guide the selection of appropriate treatments.

### Single-cell analysis of EMRGs

**Figure 7:**
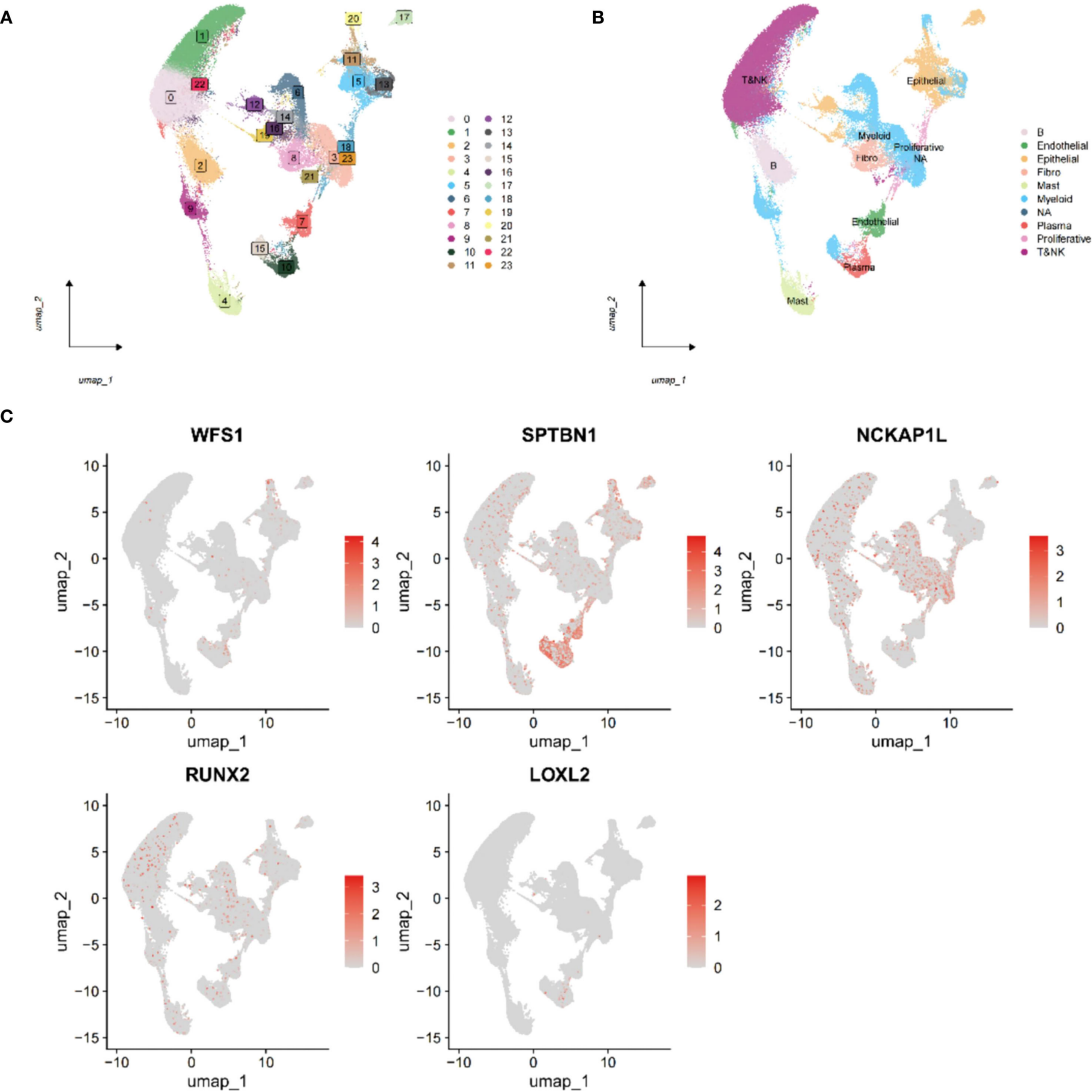
Single-cell transcriptome analysis of EMRGs. **(A)** Cell clusters identified at different resolution levels using the FindClusters function. **(B)** Cell type annotation of identified clusters. **(C)** Distribution of the five EMRGs across different cell subtypes, highlighting their cell-specific expression patterns.

To explore the interaction between immune cells and EMRGs, single-cell analysis was conducted. After quality control using Seurat (nFeature: 300–5,000; nCount: 5,000–20,000), 23,451 genes and 118,602 cells were retained. nFeature was positively correlated with nCount (r = 0.9), while percent.mt was negatively associated with nCount (r = -0.11) (**Supplementary Figures S10A–B**). Using UMAP and a clustering resolution of 0.5, 23 distinct cell subclusters were identified (**Figure 7A**) (**Supplementary Figure S10C**), annotated as Epithelial cells, B cells, Myeloid cells, Endothelial cells, Fibroblasts, Mast cells, Plasma cells, Proliferative cells, and T&NK cells (**Figure 7B**). EMRGs were mainly distributed in T&NK and Myeloid cells (**Figure 7C**). PCA demonstrated minimal batch variation, with the top nine principal components selected based on the ElbowPlot criteria (**Supplementary Figures S10D, E**).

### CeRNA network of EMRGs

A competing endogenous RNA (ceRNA) network involving lncRNA, miRNA, and mRNA was constructed, revealing that numerous lncRNAs and miRNAs could potentially be regulated by EMRGs. Offering mechanistic insights into LUAD, this network may inform the design of future therapeutic interventions (**Supplementary Figure S11**) (**Supplementary Table S8**).

### Verify protein and mRNA expression of EMRGs

**Figure 8:**
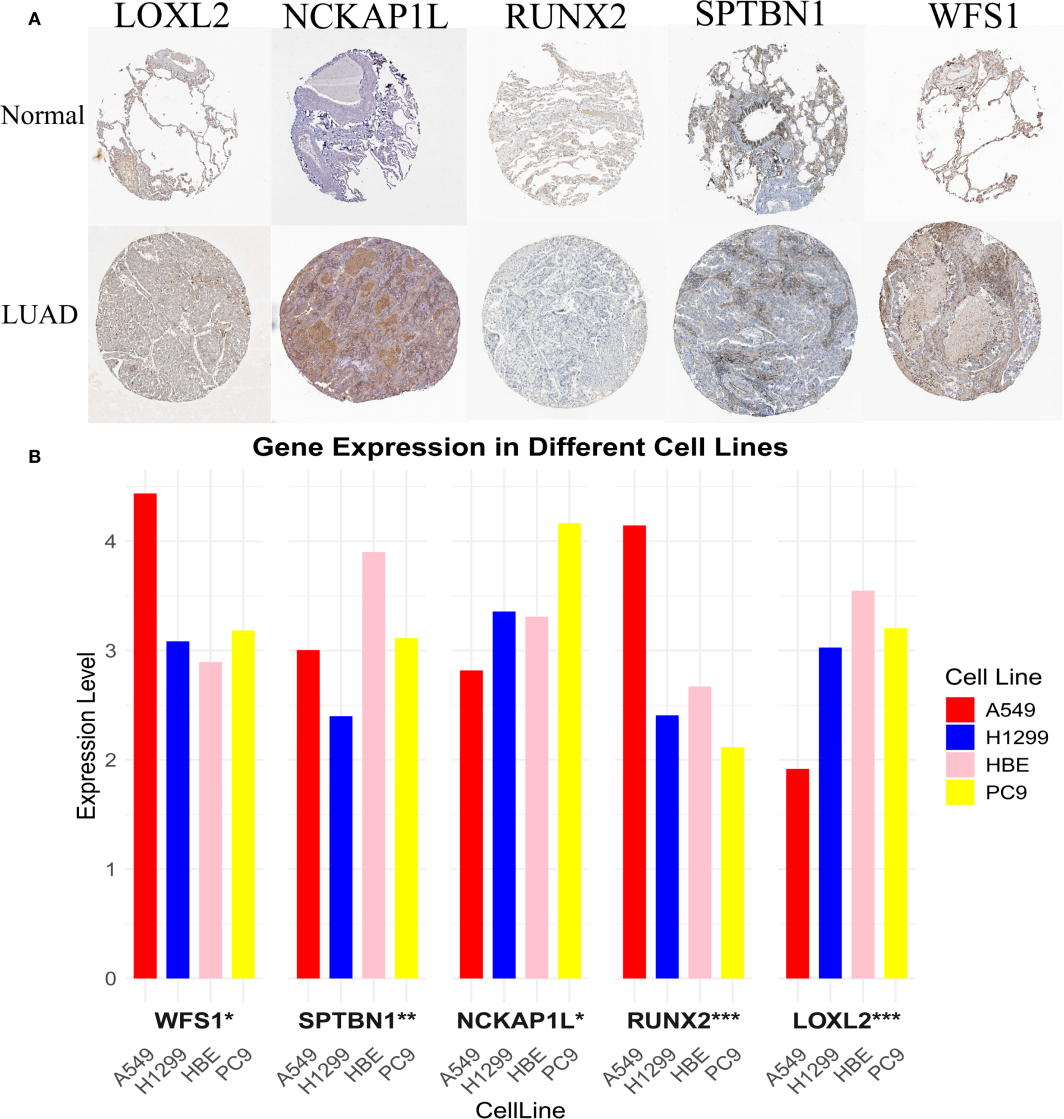
Experimental validation of EMRG expression. **(A)** mRNA expression profiles of EMRGs from the Human Protein Atlas (HPA). **(B)** RT-qPCR validation of EMRG expression in LUAD cell lines compared with normal cell lines. *P < 0.05, **P < 0.01, ***P < 0.001.

We analyzed the protein expression of EMRGs in both t- and n-tissues (**Figure 8A**). In addition, RT-qPCR analysis revealed that LOXL2 and SPTBN1 were predominantly expressed in normal cell lines, whereas higher levels of NCKAP1L, RUNX2, and WFS1 were detected in tumor cell lines (**Figure 8B**).

## Discussion

Our study highlights the urgent need for robust, biology-informed prognostic models that integrate molecular and clinical data in LUAD (33). We employed 117 ML algorithm pairings and MR analysis to establish a prognostic model involving five EMRGs. This model consistently stratified patients by OS in TCGA and GEO cohorts, with AUC values exceeding 0.70 across time points. The combination of stepwise Cox regression and GBM yielded the highest C-index, underscoring the rigor of our multi-algorithm approach. The nomogram constructed from this model showed excellent calibration and clinical benefit in DCA, outperforming clinical features alone. Furthermore, the model proved robust across different age, stage, and smoking subgroups. Its strong association with TMB supports a mechanistic link between metabolic reprogramming and genomic instability. The five EMRGs in our model—LOXL2, RUNX2, NCKAP1L, WFS1, and SPTBN1—have been implicated in diverse cancer-related processes (34). LOXL2 regulates ECM remodeling and stiffness, a key factor in tumor invasion and metastasis, often via ZEB1-mediated upregulation (35). RUNX2 promotes glycolysis and enhances invasiveness through HDAC-dependent osteopontin splicing in NSCLC (36). NCKAP1L, mainly expressed in immune cells, regulates actin dynamics and T cell activation, bridging metabolism and immune modulation (37). The oncogenic SPTBN1–ALK fusion may drive resistance to therapy by enhancing cytoskeletal signaling and tumor adaptability (38).

Of note, growing evidence has highlighted the regulatory role of N6-methyladenosine (m^6^A) RNA methylation in shaping cancer metabolism and immune escape. Recent studies suggest that m^6^A “writers”, “erasers”, and “readers” dynamically regulate the stability and translation of EMRGs (39, 40). The scRNA-seq analysis revealed specific gene expression in different cell types, suggesting their involvement in transcriptional regulation, immune evasion, and stromal remodeling in the TME. These effects can be conceptualized in three mechanistic axes: In T/NK cells, EMRGs impair cytotoxic function via immune checkpoint activation and altered receptor signaling (41). In myeloid cells, they promote immune tolerance through IL-1 family cytokines, PD-L1 expression, and IDO1-mediated suppression (42). In fibroblasts, ECM regulators such as LOX and MMP11 reinforce tumor-supportive stroma and impede immune infiltration (43–47). Functional enrichment analysis further supported this, with the high-risk group enriched in ECM-receptor interaction and DNA replication licensing pathways. The former mediates tumor-stroma interactions and physical barriers to immunity, while the latter reflects uncontrolled S-phase entry and genomic instability—both hallmarks of aggressive LUAD (48, 49).

In addition to prognostication, our model offers therapeutic insight. Apoptotic agents, particularly Docetaxel and Sepantronium bromide, were more effective in the high-risk LUAD population (50, 51). Mechanistically, immune-excluded tumors may lack the immune-based resistance seen in “hot” tumors, making them more reliant on intrinsic survival pathways (e.g., BCL2 family). Targeting these may overcome resistance in metabolically reprogrammed tumors. Conversely, low-risk patients responded better to CDK9 inhibitors such as CDK9_5038, CDK9_5576, and Dinaciclib, suggesting distinct therapeutic vulnerabilities based on metabolic phenotype. TIDE analysis further supported clinical utility: high-risk patients had elevated T-cell dysfunction and TIDE scores, indicating an immunosuppressive microenvironment and potentially poorer ICI response (52, 53). Meanwhile, low-risk patients exhibited stronger immune infiltration and HLA expression, favoring ICI responsiveness. These findings underscore the value of EMRG signatures in capturing the interplay between tumor metabolism and immune dynamics, aiding both prognosis and treatment selection (54, 55).

Our study introduces several innovations: MR was used to select genes with causal associations, reducing bias inherent in correlation-based models; multiple ML algorithms were systematically tested for optimal performance; single-cell transcriptomic validation provided cellular-resolution insight; and a clinically interpretable nomogram was built to bridge research and clinical application. Compared to existing LUAD models, which often rely on bulk RNA-seq or single-pathway markers, our multi-omics framework offers enhanced robustness and translational potential. Nonetheless, limitations exist. First, all training and validation data were retrospective, introducing potential bias. Second, while MR mitigates confounding, it relies on the quality of eQTL datasets and may not eliminate pleiotropy. Our drug sensitivity predictions provide useful hypotheses for potential therapeutic options. However, these results are derived entirely from in silico analysis and their clinical relevance remains hypothetical. Further preclinical and clinical validation will be required. Lastly, our scRNA-seq analysis was based on nine LUAD samples, which is relatively small. This limited sample size may restrict the generalizability of cell-type specific conclusions. Validation in larger single-cell cohorts will be important to confirm our findings. We were unable to validate the link between EMRG-defined subgroups and ICI responsiveness in independent LUAD cohorts due to lack of available data. Therefore, our conclusions regarding immunotherapy response remain indirect. Future validation in ICI-treated cohorts is warranted.

In brief, we designed and confirmed the accuracy of an EMRG-based model to predict LUAD outcomes using MR, ML, and single-cell transcriptomic data. This model effectively stratifies patients by survival risk and offers mechanistic insights and therapeutic guidance, advancing personalized medicine in LUAD.

## Conclusion

Our prognostic model based on 5 EMRGs and validated its performance in multiple LUAD cohorts, when combined with clinical factors, the constructed nomogram further enhances its value in prognostic assessment. HRG exhibits higher TMB, impaired T cell function, and ECM remodeling, which may lead to poor outcomes. Drug susceptibility analysis suggests that HRG may benefit from docetaxel and sepanium bromide, while LRG responds better to CDK9-targeted agents. The scRNA-seq reveals different EMRG expression patterns across different cell types, leading to a better understanding of tumor heterogeneity.

## Data Availability

The datasets presented in this study can be found in online repositories. The names of the repository/repositories and accession number(s) can be found in the article/**Supplementary Material**.

## Supplementary material

Supplementary Figure 1 Forest plots for MR results of selected EMRGs.

Supplementary Figure 2 Scatter plots for MR results of selected EMRGs.

Supplementary Figure 3 K-M and ROC curves for TCGA-LUAD dataset (A-B), for GSE72094 dataset (C-D), for GSE50081 dataset (E-F), for GSE30219 dataset (G-H).

Supplementary Figure 4 Internal training and external validation of the gene signature prediction model. Risk distribution and survival scatter plots, and gene differential expression heat maps in TCGA cohort (A), in GSE72094 cohort (B), in GSE50081 cohort (C), and in GSE30219 cohort (D).

Supplementary Figure 5 Scatter plots showing association between clinical features and risk scores in TCGA cohort (A-D); Heatmap integrating clinical traits with expression levels of 5 model genes (E).

Supplementary Figure 6 Stratified survival analysis of the EMRGs model across clinical subgroups in TCGA-LUAD (A-K).

Supplementary Figure 7 KEGG enrichment analysis. Circle chart (A); GO enrichment. Analysis Bar chart (B).

Supplementary Figure 8 Correlation analysis between risk score and immune infiltration under multiple algorithms.

Supplementary Figure 10 TIDE analysis comparing T cell dysfunction (A), T cell exclusion (B), and TIDE score (C) between HRG and LRG.

Supplementary Figure 11 ScRNA analysis of EMRGs. Quality control and parameter selection in single-cell RNA-seq data (A-B); Clusters determined at different resolution in FindClusters function (C); PCA dimension reduction and ElbowPlot identifying major PCs (D-E).

Supplementary Figure 12 ceRNA network of EMRGs constructed by integrating lncRNA-miRNA-mRNA relationships.

Supplementary Table 1 Primer sequences for 5 EMRGs.

Supplementary Table 2 Mendelian randomization results of 254 EMRGs significantly associated with LUAD across five MR methods.

Supplementary Table 3 The 9 overlapping genes identified from MR and DEG intersection.

Supplementary Table 4 Cochran’s Q and Egger intercept tests of heterogeneity and pleiotropy in MR analysis.

Supplementary Table 5 KEGG enrichment analysis results of DEGs between HRG&LRG.

Supplementary Table 6 GO enrichment analysis results of DEGs between HRG&LRG.

Supplementary Table 7 Estimated IC50 values of 89 antitumor drugs for HRG&LRG.

Supplementary Table 8 Predicted lncRNA-miRNA-mRNA interactions in the EM-gene-associated ceRNA network.

## Glossary

AUC: Area Under the Curve
CBCAS: Cell Bank of the Chinese Academy of Sciences
DCA: Decision Curve Analysis
DEGs: Differentially Expressed Genes
EM: Energy Metabolism
EMRGs: Energy Metabolism-Related Genes
eQTL: Expression Quantitative Trait Loci
FDR: False Discovery Rate
GBM: Gradient Boosting Machine
GDSC: Genomics of Drug Sensitivity in Cancer
GEO: Gene Expression Omnibus
GO: Gene Ontology
HPA: Human Protein Atlas
HR: Hazard Ratio
HRG: High-Risk Group
IC50: Half Maximal Inhibitory Concentration
ICIs: Immune Checkpoint Inhibitors
IV: Instrumental Variable
IVW: Inverse Variance Weighted
KEGG: Kyoto Encyclopedia of Genes and Genomes
K-M: Kaplan–Meier
LASSO: Least Absolute Shrinkage and Selection Operator
LD: Linkage Disequilibrium
LOOCV: Leave-One-Out Cross-Validation
LRG: Low-Risk Group
LUAD: Lung Adenocarcinoma
MR: Mendelian Randomization
NSCLC: Non-Small Cell Lung Cancer
OR: Odds Ratio
PCA: Principal Component Analysis
plsRcox: Partial Least Squares Regression for Cox Models
RF: Random Forest
ROC: Receiver Operating Characteristic
RSF: Random Survival Forest
RT-qPCR: Reverse Transcription Quantitative Polymerase Chain Reaction
scRNA-seq: Single-Cell RNA Sequencing
SNP: Single Nucleotide Polymorphism
TCGA: The Cancer Genome Atlas
TIDE: Tumor Immune Dysfunction and Exclusion
TIMER: Tumor Immune Estimation Resource
TMB: Tumor Mutational Burden
TME: Tumor Microenvironment
UMAP: Uniform Manifold Approximation and Projection.
