# Supplementary figures and images for: Integrative multi-omics reveals energy metabolism–related prognostic signatures and immunogenetic landscapes in lung adenocarcinoma
Source: Front Immunol. 2025 Oct 14;16:1679464. doi: 10.3389/fimmu.2025.1679464 (PMC12558868; doi:10.3389/fimmu.2025.1679464)

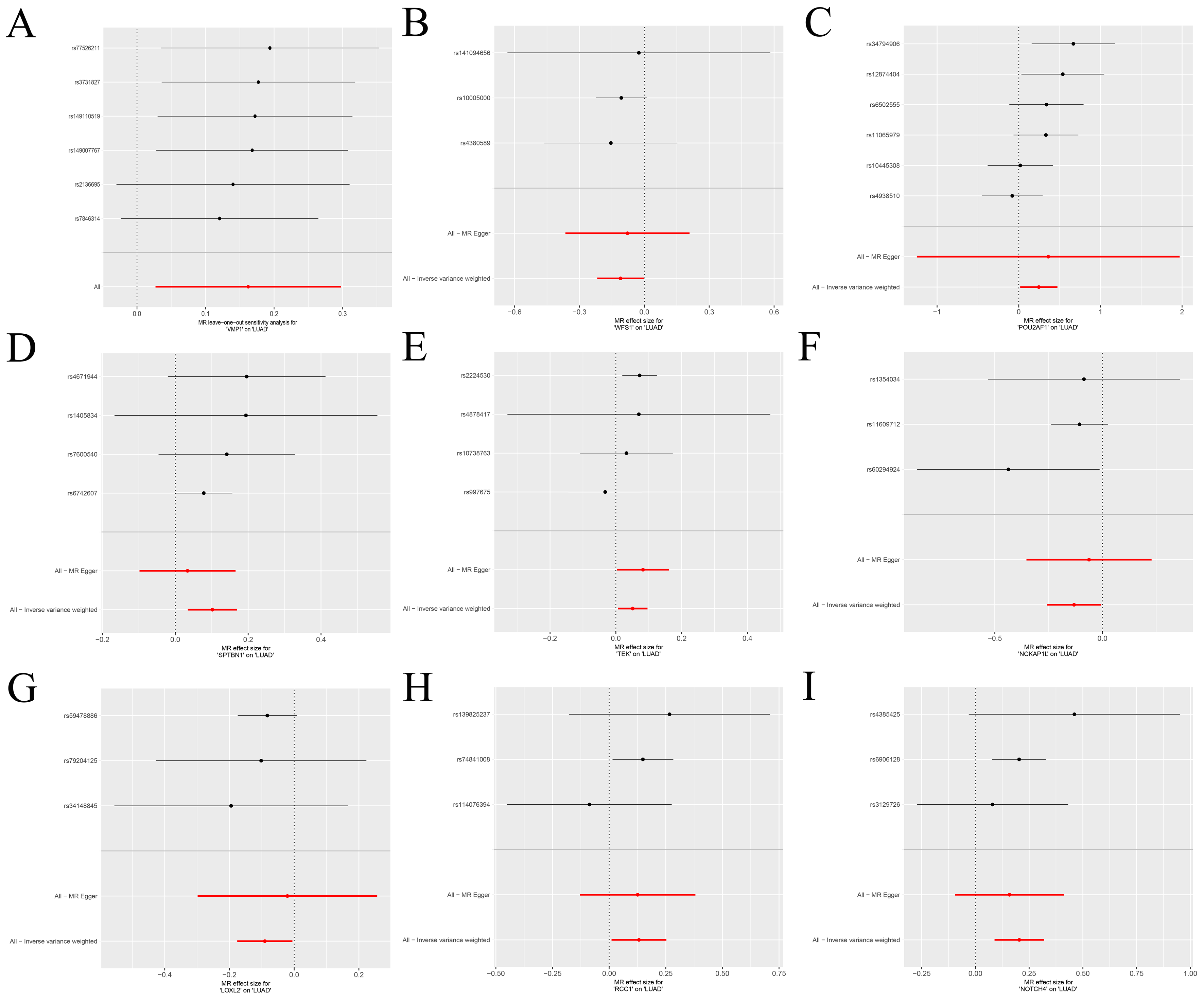

Supplement: Supplementary Figure 1 — Forest plots for MR results of selected EMRGs. [file Image1.tif]

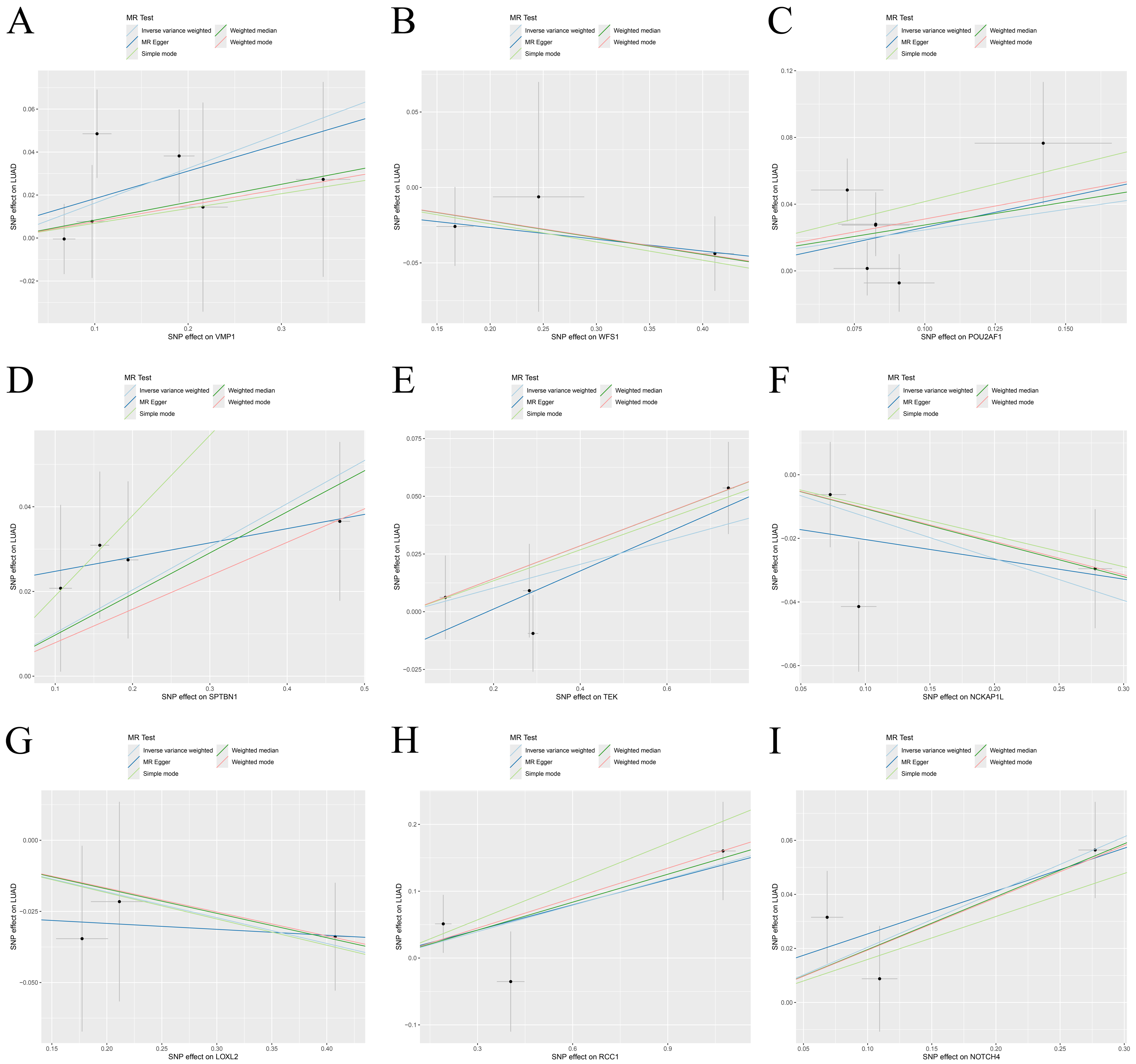

Supplement: Supplementary Figure 2 — Scatter plots for MR results of selected EMRGs. [file Image2.tif]

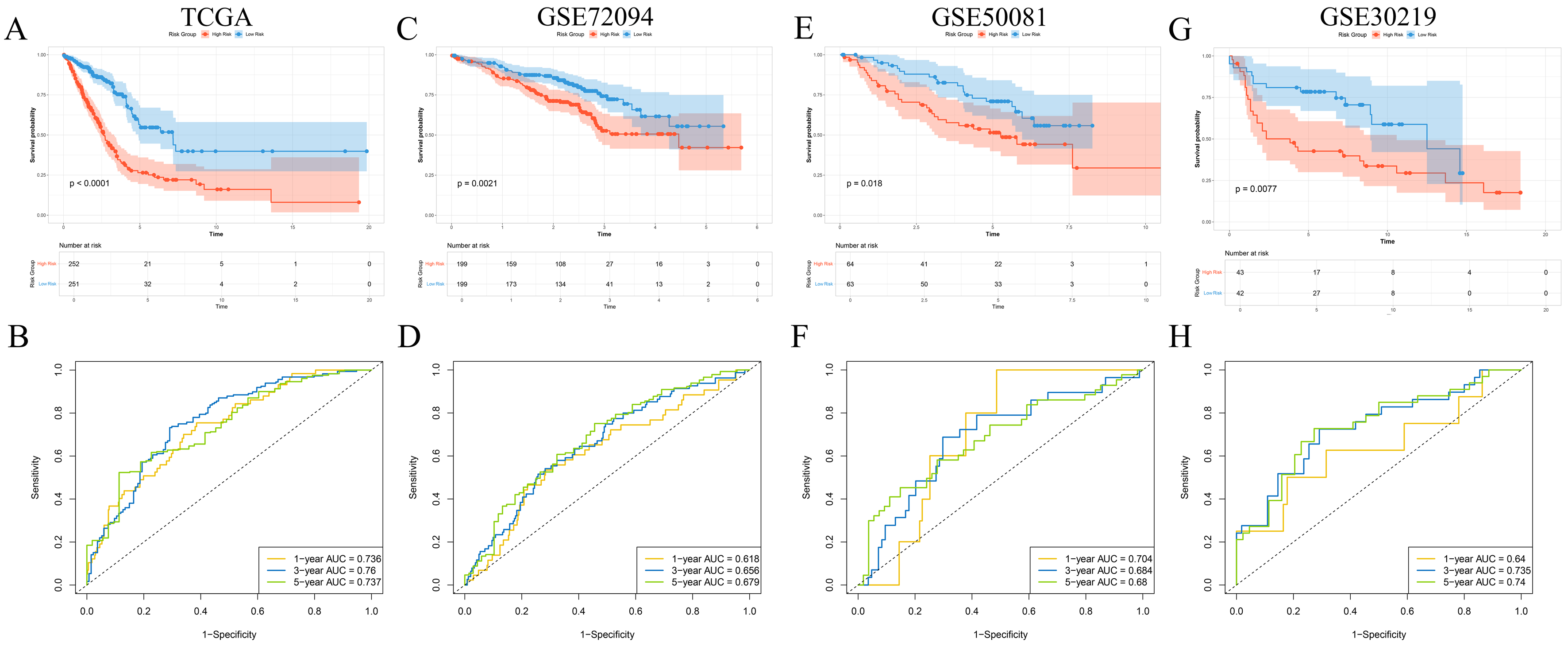

Supplement: Supplementary Figure 3 — K-M and ROC curves for TCGA-LUAD dataset (A-B), for GSE72094 dataset (C-D), for GSE50081 dataset (E-F), for GSE30219 dataset (G-H). [file Image3.tif]

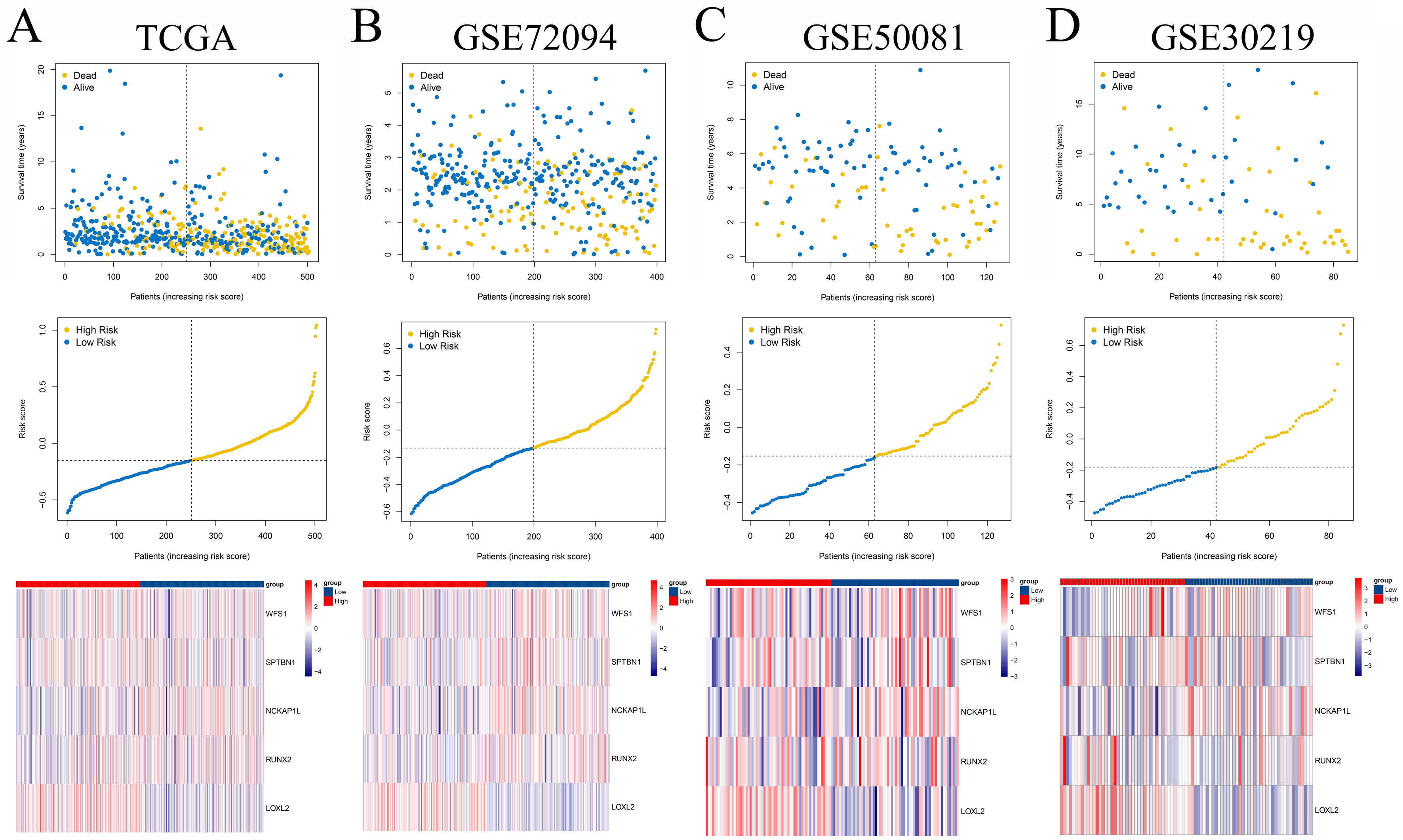

Supplement: Supplementary Figure 4 — Internal training and external validation of the gene signature prediction model. Risk distribution and survival scatter plots, and gene differential expression heat maps in TCGA cohort (A), in GSE72094 cohort (B), in GSE50081 cohort (C), and in GSE30219 cohort (D). [file Image4.tif]

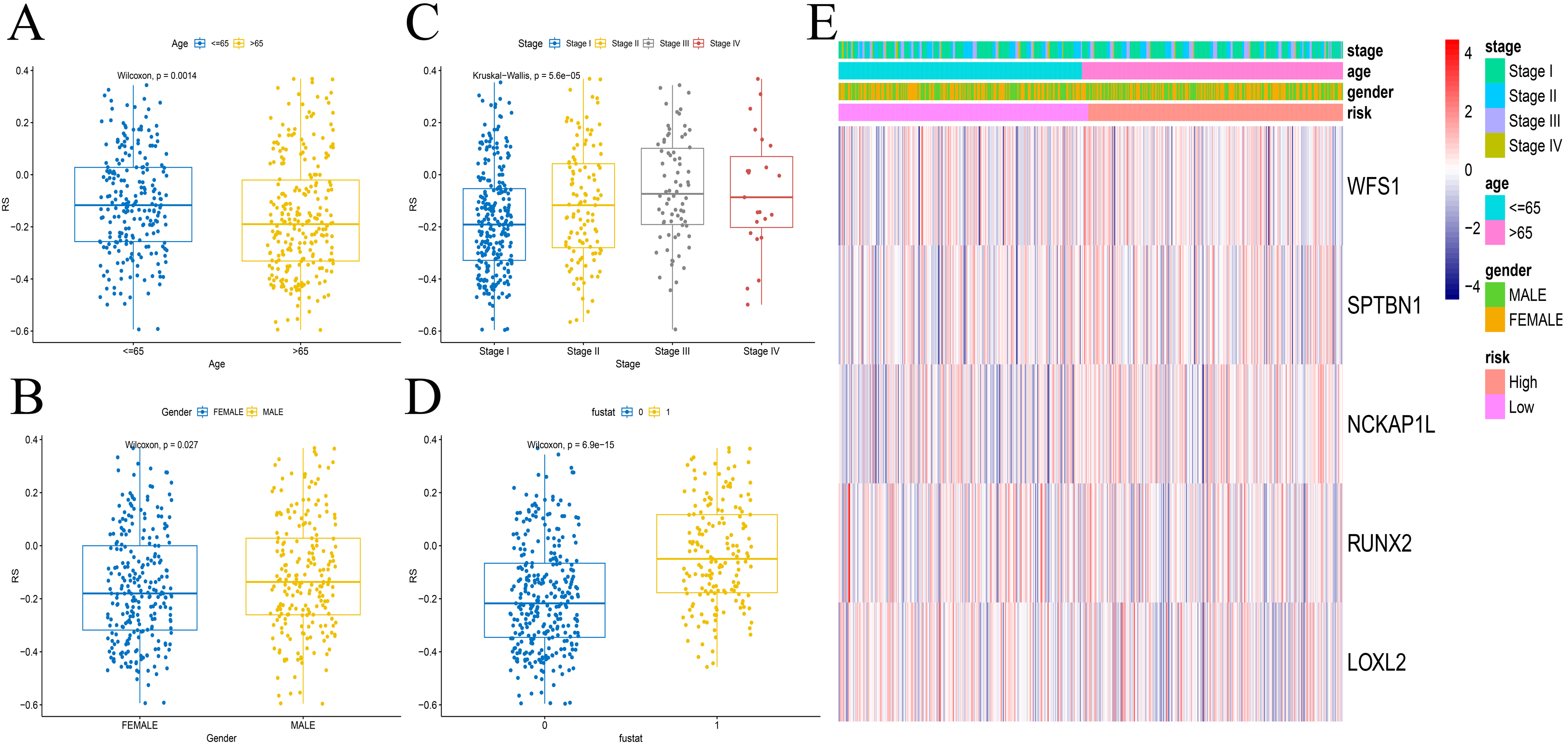

Supplement: Supplementary Figure 5 — Scatter plots showing association between clinical features and risk scores in TCGA cohort (A-D); Heatmap integrating clinical traits with expression levels of 5 model genes (E). [file Image5.tif]

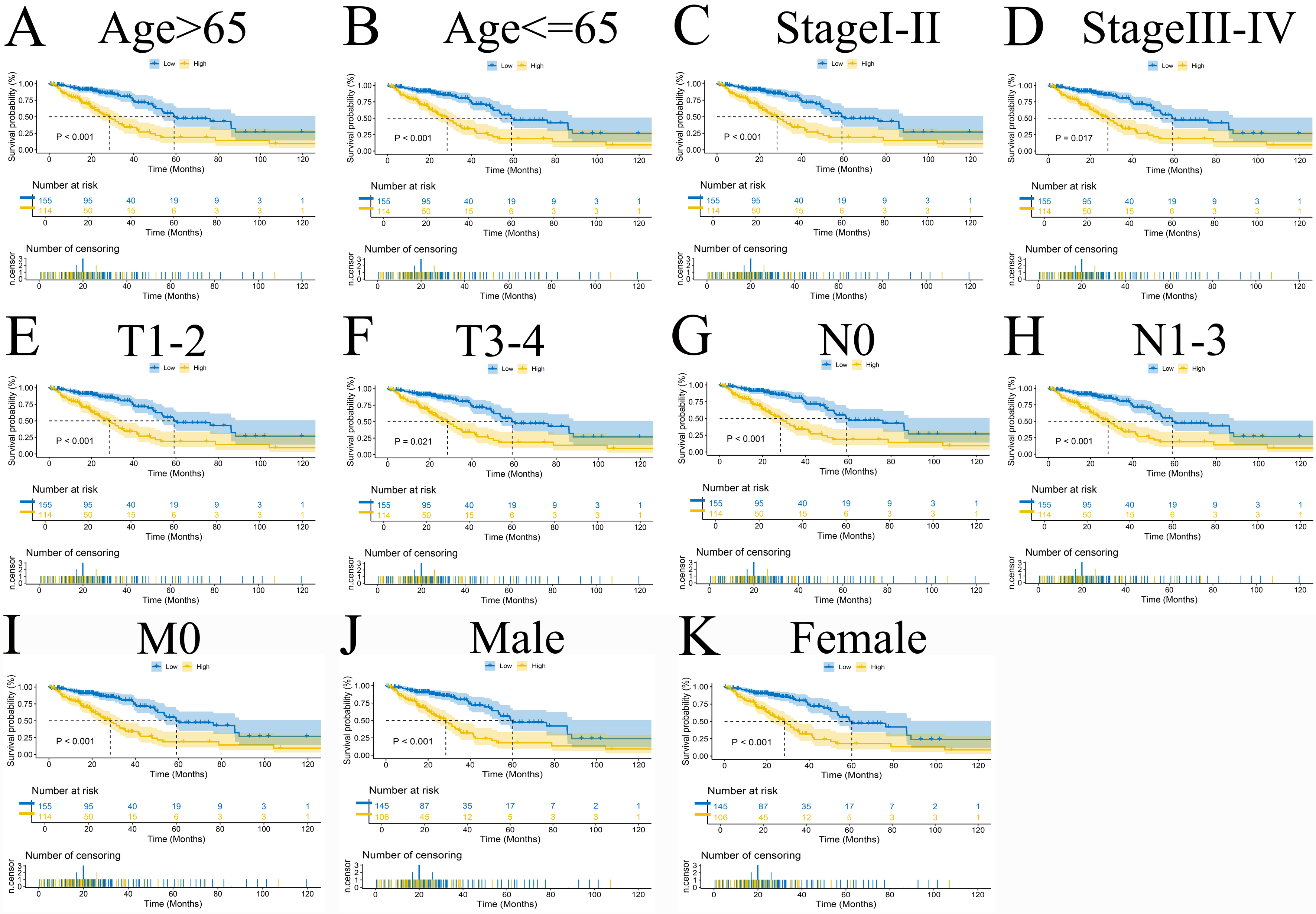

Supplement: Supplementary Figure 6 — Stratified survival analysis of the EMRGs model across clinical subgroups in TCGA-LUAD (A-K). [file Image6.tif]

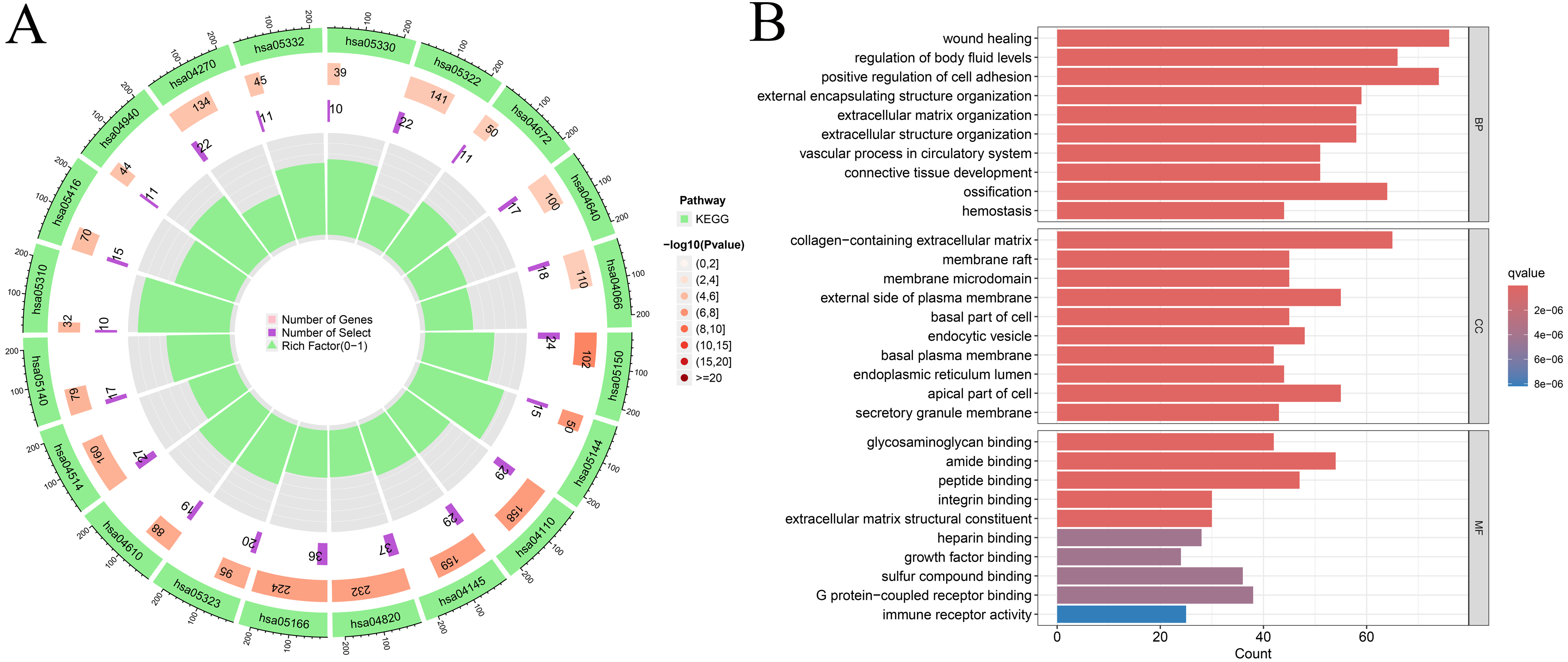

Supplement: Supplementary Figure 7 — KEGG enrichment analysis. Circle chart (A); GO enrichment. Analysis Bar chart (B). [file Image7.tif]

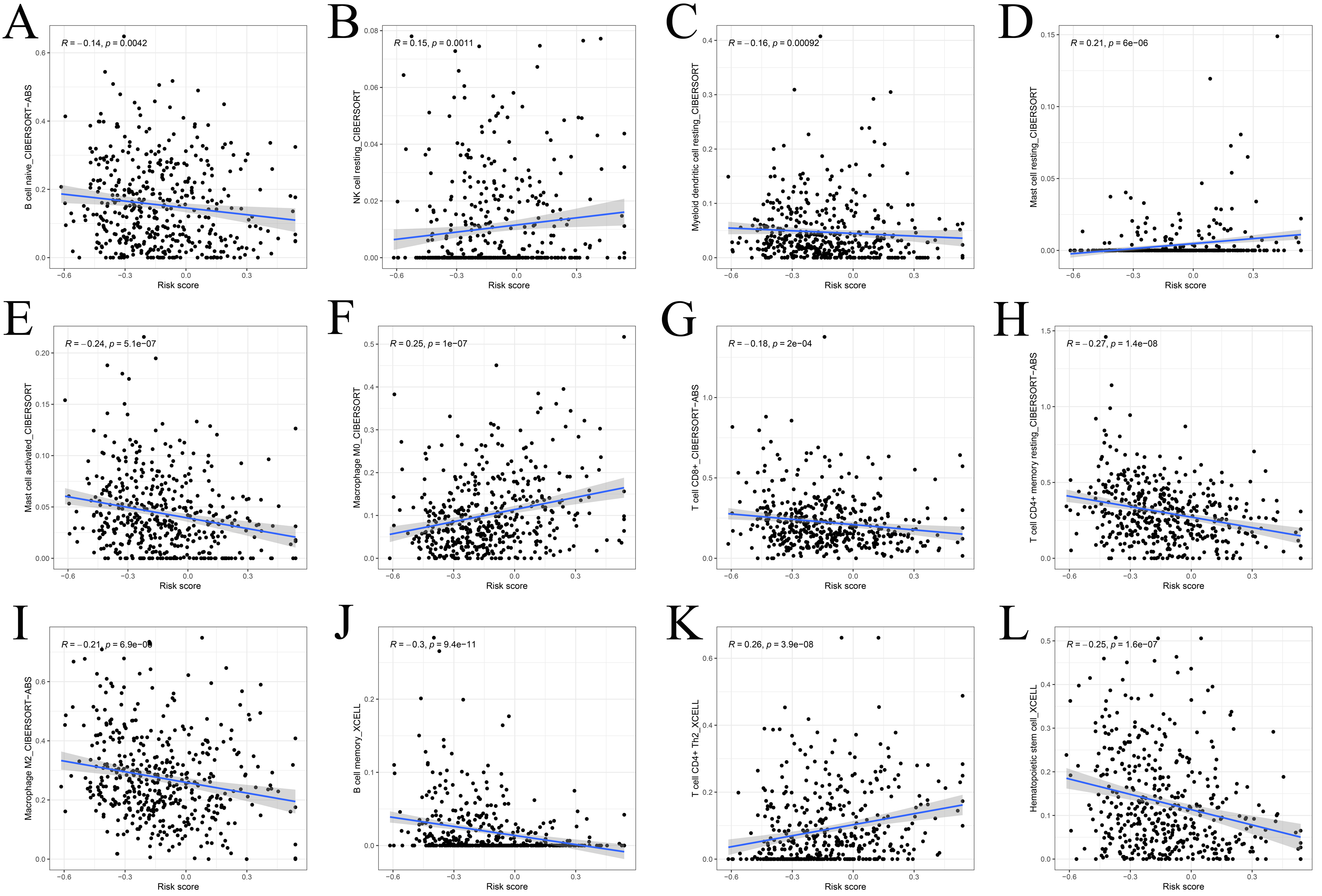

Supplement: Supplementary Figure 8 — Correlation analysis between risk score and immune infiltration under multiple algorithms. [file Image8.tif]

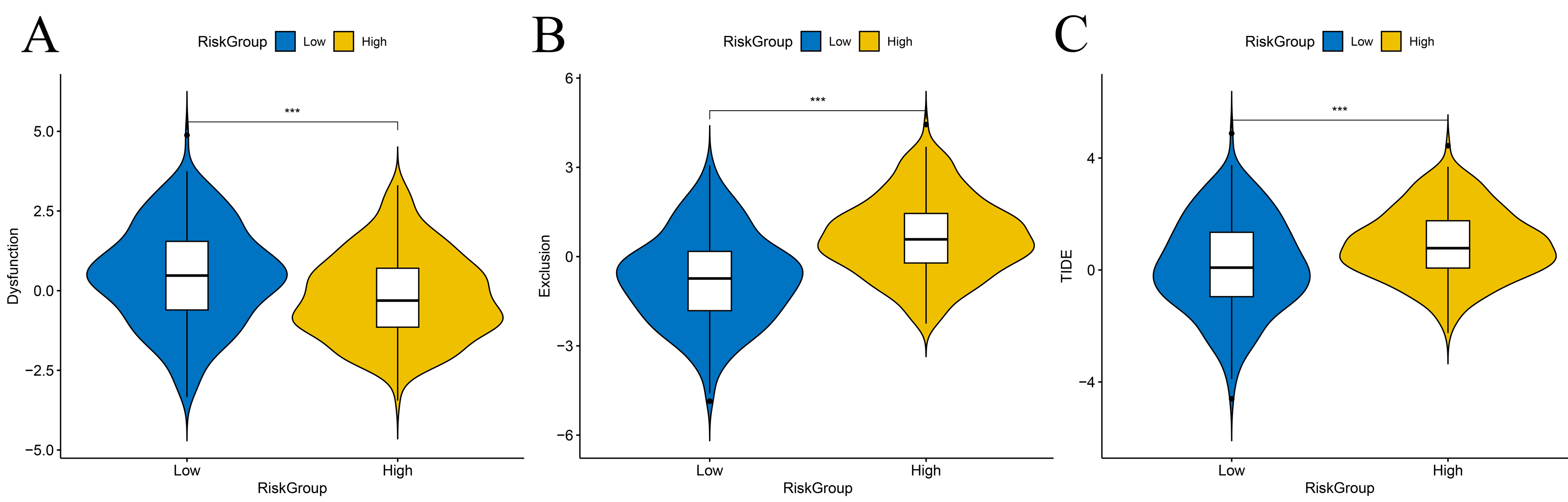

Supplement: Supplementary Figure 10 — TIDE analysis comparing T cell dysfunction (A), T cell exclusion (B), and TIDE score (C) between HRG and LRG. [file Image9.tif]

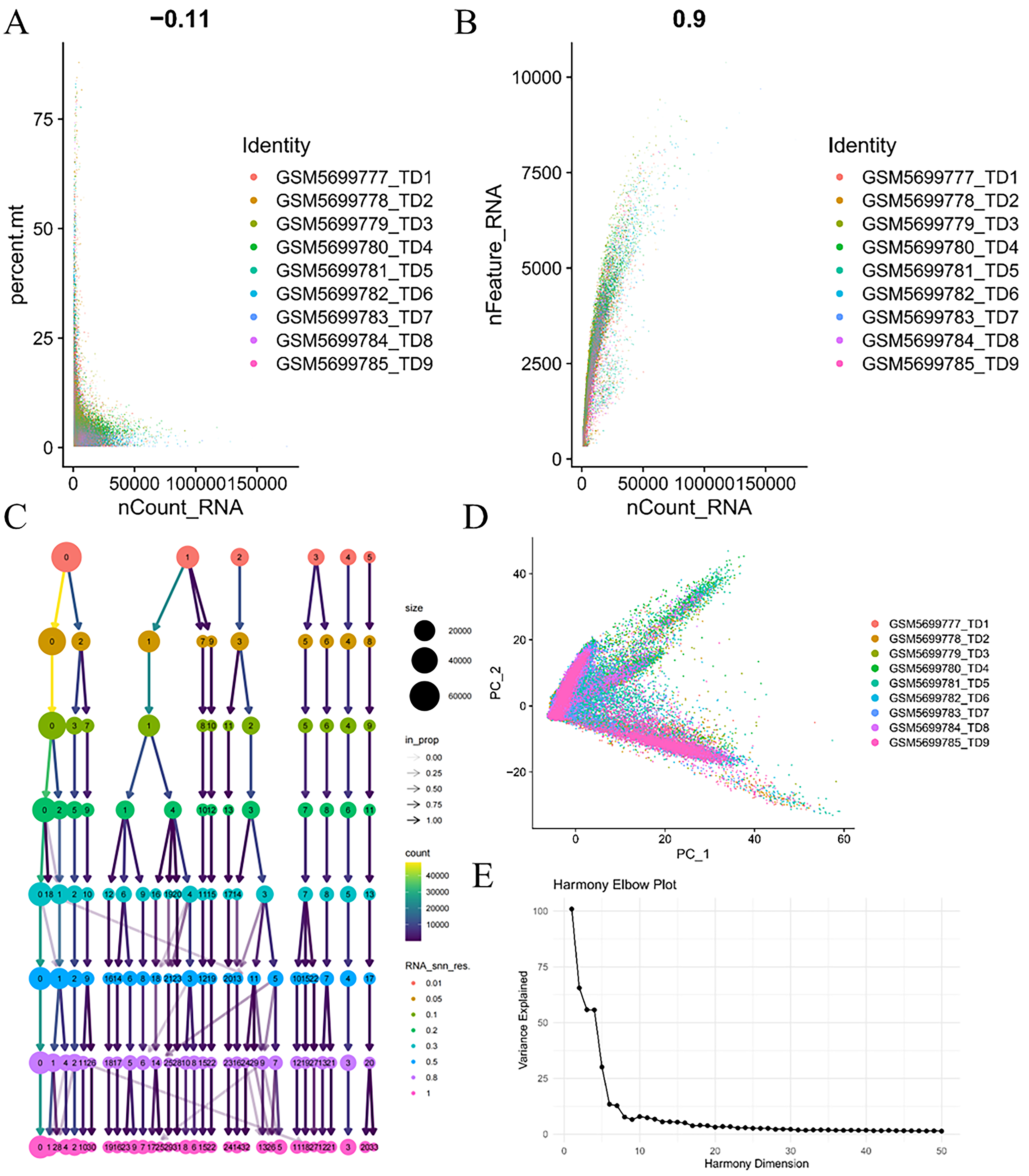

Supplement: Supplementary Figure 11 — ScRNA analysis of EMRGs. Quality control and parameter selection in single-cell RNA-seq data (A-B); Clusters determined at different resolution in FindClusters function (C); PCA dimension reduction and ElbowPlot identifying major PCs (D-E). [file Image10.tif]

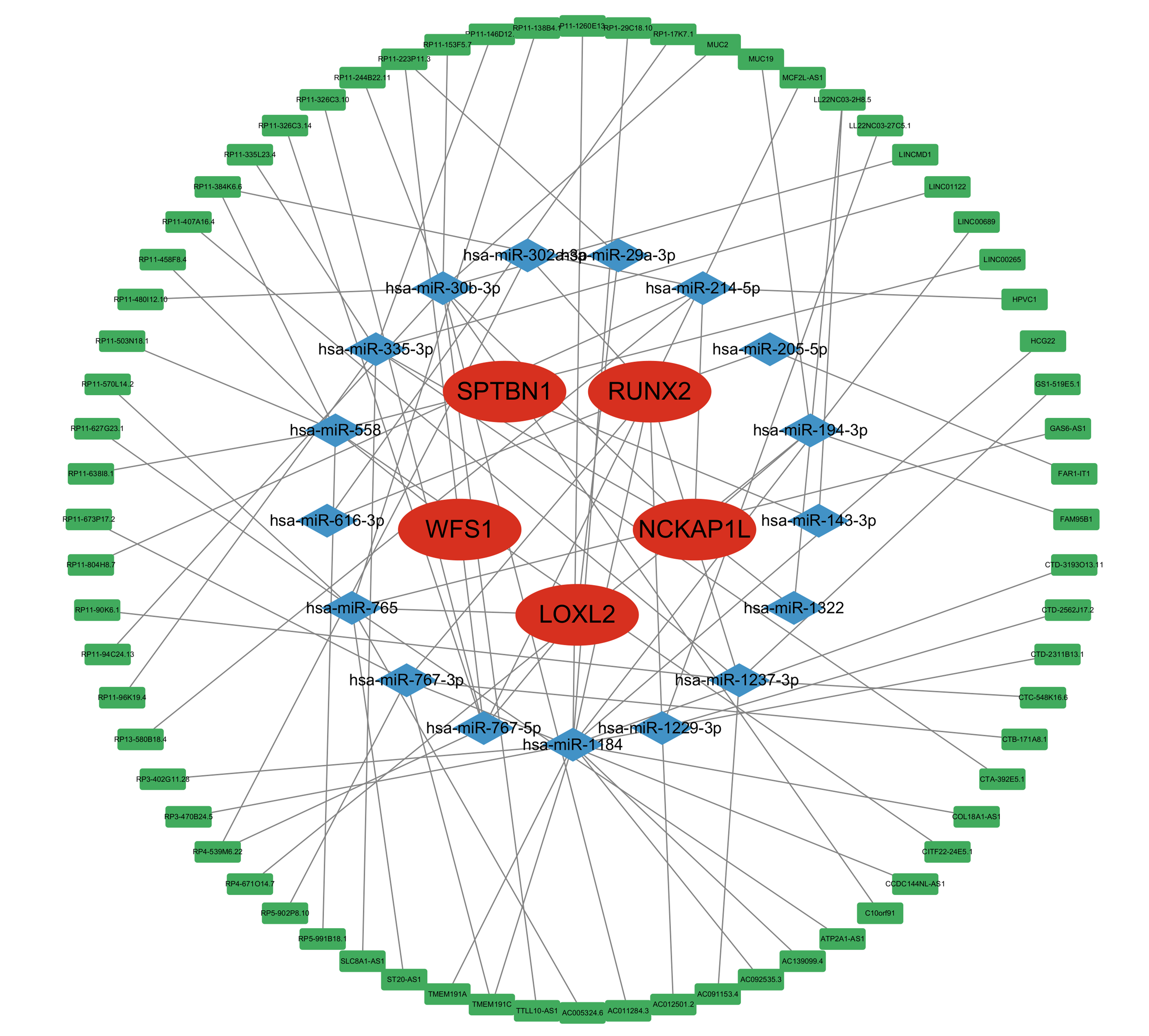

Supplement: Supplementary Figure 12 — ceRNA network of EMRGs constructed by integrating lncRNA-miRNA-mRNA relationships. [file Image11.tif]
