# Supplementary material for: Integrative multi-omics reveals energy metabolism–related prognostic signatures and immunogenetic landscapes in lung adenocarcinoma
Source: Front Immunol. 2025 Oct 14;16:1679464. doi: 10.3389/fimmu.2025.1679464 (PMC12558868; doi:10.3389/fimmu.2025.1679464)
Supplement: Supplementary Table 1 — Primer sequences for 5 EMRGs. [file Table1.docx]

**Table S1** Primer sequences for 5 EN-related mRNAs.

| **Gene id** | **Forward Primer** | **Reverse Primer** |
| --- | --- | --- |
| WFS1 | GTTCCCGACTCAATGCCACA | CCGCTGCGTCTCTAACACC |
| RUNX2 | TGGTTACTGTCATGGCGGGTA | TCTCAGATCGTTGAACCTTGCTA |
| SPTBN1 | GGGGTCCCATGACATCGTG | CCGGTGTTTGTGTATCAGTGC |
| LOXL2 | AGGACATTCGGATTCGAGCC | CTTCCTCCGTGAGGCAAAC |
| NCKAP1L | GGACCAGTACATCGTGAAAAAGC | TGATCCCGAAATTCCATGACATC |

**Abbreviations:** EN: Energy metabolism; mRNA: Messenger RNA.
