# Supplementary material for: Integrative multi-omics reveals energy metabolism–related prognostic signatures and immunogenetic landscapes in lung adenocarcinoma
Source: Front Immunol. 2025 Oct 14;16:1679464. doi: 10.3389/fimmu.2025.1679464 (PMC12558868; doi:10.3389/fimmu.2025.1679464)
Supplement: Supplementary Table 2 — Mendelian randomization results of 254 EMRGs significantly associated with LUAD across five MR methods. [file Table2.docx]

**Table S2** The MR analysis of causal relationship between genes and LUAD based on five methods.

| **Exposure** | **method** | **Nsnp** | **B** | **Se** | **pval** | **OR** |
| --- | --- | --- | --- | --- | --- | --- |
| ABHD14B | MR Egger | 3 | -0.04 | 0.22 | 0.88 | 0.96 |
|  | Weighted median | 3 | -0.12 | 0.06 | 0.05 | 0.89 |
|  | Inverse variance weighted | 3 | -0.12 | 0.06 | 0.04 | 0.89 |
|  | Simple mode | 3 | -0.17 | 0.09 | 0.18 | 0.84 |
|  | Weighted mode | 3 | -0.15 | 0.07 | 0.16 | 0.86 |
| ABO | MR Egger | 3 | 0.01 | 0.3 | 0.97 | 1.01 |
|  | Weighted median | 3 | 0.11 | 0.04 | 0.01 | 1.12 |
|  | Inverse variance weighted | 3 | 0.11 | 0.05 | 0.01 | 1.12 |
|  | Simple mode | 3 | 0.07 | 0.06 | 0.35 | 1.08 |
|  | Weighted mode | 3 | 0.11 | 0.05 | 0.16 | 1.11 |
| ACADM | MR Egger | 4 | -0.05 | 0.09 | 0.63 | 0.95 |
|  | Weighted median | 4 | -0.07 | 0.04 | 0.05 | 0.93 |
|  | Inverse variance weighted | 4 | -0.09 | 0.05 | 0.05 | 0.91 |
|  | Simple mode | 4 | -0.08 | 0.07 | 0.31 | 0.92 |
|  | Weighted mode | 4 | -0.07 | 0.04 | 0.15 | 0.93 |
| ADGRE4P | MR Egger | 3 | -0.1 | 0.09 | 0.47 | 0.9 |
|  | Weighted median | 3 | -0.08 | 0.04 | 0.05 | 0.92 |
|  | Inverse variance weighted | 3 | -0.09 | 0.04 | 0.04 | 0.92 |
|  | Simple mode | 3 | -0.04 | 0.07 | 0.61 | 0.96 |
|  | Weighted mode | 3 | -0.08 | 0.04 | 0.19 | 0.92 |
| ADM | MR Egger | 7 | -0.08 | 0.07 | 0.3 | 0.92 |
|  | Weighted median | 7 | -0.08 | 0.04 | 0.05 | 0.92 |
|  | Inverse variance weighted | 7 | -0.07 | 0.04 | 0.04 | 0.93 |
|  | Simple mode | 7 | -0.07 | 0.07 | 0.36 | 0.93 |
|  | Weighted mode | 7 | -0.08 | 0.04 | 0.09 | 0.92 |
| ANKRD50 | MR Egger | 3 | 0.07 | 0.06 | 0.45 | 1.07 |
|  | Weighted median | 3 | 0.09 | 0.04 | 0.03 | 1.09 |
|  | Inverse variance weighted | 3 | 0.09 | 0.04 | 0.03 | 1.1 |
|  | Simple mode | 3 | 0.17 | 0.08 | 0.18 | 1.18 |
|  | Weighted mode | 3 | 0.09 | 0.04 | 0.15 | 1.09 |
| AP2M1 | MR Egger | 5 | -0.05 | 0.06 | 0.43 | 0.95 |
|  | Weighted median | 5 | -0.09 | 0.04 | 0.05 | 0.92 |
|  | Inverse variance weighted | 5 | -0.09 | 0.04 | 0.02 | 0.91 |
|  | Simple mode | 5 | -0.24 | 0.1 | 0.09 | 0.79 |
|  | Weighted mode | 5 | -0.07 | 0.04 | 0.17 | 0.93 |
| AP3M1 | MR Egger | 5 | 0.14 | 0.12 | 0.35 | 1.15 |
|  | Weighted median | 5 | 0.12 | 0.05 | 0.01 | 1.13 |
|  | Inverse variance weighted | 5 | 0.12 | 0.04 | 0.01 | 1.12 |
|  | Simple mode | 5 | 0.07 | 0.09 | 0.47 | 1.07 |
|  | Weighted mode | 5 | 0.13 | 0.05 | 0.05 | 1.14 |
| ARHGAP33 | MR Egger | 3 | 0.02 | 0.07 | 0.8 | 1.02 |
|  | Weighted median | 3 | 0.07 | 0.03 | 0.04 | 1.07 |
|  | Inverse variance weighted | 3 | 0.07 | 0.03 | 0.02 | 1.08 |
|  | Simple mode | 3 | 0.07 | 0.04 | 0.2 | 1.07 |
|  | Weighted mode | 3 | 0.07 | 0.03 | 0.18 | 1.07 |
| ARL16 | MR Egger | 4 | 0.08 | 0.05 | 0.23 | 1.09 |
|  | Weighted median | 4 | 0.08 | 0.03 | 0.01 | 1.08 |
|  | Inverse variance weighted | 4 | 0.07 | 0.03 | 0.01 | 1.07 |
|  | Simple mode | 4 | 0.08 | 0.04 | 0.17 | 1.08 |
|  | Weighted mode | 4 | 0.08 | 0.03 | 0.09 | 1.08 |
| ASB16-AS1 | MR Egger | 4 | -0.04 | 0.16 | 0.83 | 0.96 |
|  | Weighted median | 4 | -0.08 | 0.04 | 0.09 | 0.93 |
|  | Inverse variance weighted | 4 | -0.08 | 0.04 | 0.04 | 0.92 |
|  | Simple mode | 4 | -0.04 | 0.09 | 0.65 | 0.96 |
|  | Weighted mode | 4 | -0.08 | 0.04 | 0.19 | 0.93 |
| ASNSD1 | MR Egger | 3 | 0.05 | 0.05 | 0.51 | 1.05 |
|  | Weighted median | 3 | 0.07 | 0.04 | 0.06 | 1.08 |
|  | Inverse variance weighted | 3 | 0.08 | 0.04 | 0.04 | 1.08 |
|  | Simple mode | 3 | 0.14 | 0.08 | 0.2 | 1.15 |
|  | Weighted mode | 3 | 0.07 | 0.04 | 0.22 | 1.07 |
| ATF6 | MR Egger | 8 | 0.07 | 0.04 | 0.12 | 1.08 |
|  | Weighted median | 8 | 0.06 | 0.03 | 0.09 | 1.06 |
|  | Inverse variance weighted | 8 | 0.06 | 0.03 | 0.05 | 1.06 |
|  | Simple mode | 8 | 0.02 | 0.07 | 0.77 | 1.02 |
|  | Weighted mode | 8 | 0.05 | 0.04 | 0.17 | 1.06 |
| ATP10A | MR Egger | 4 | -0.07 | 0.14 | 0.67 | 0.93 |
|  | Weighted median | 4 | -0.1 | 0.04 | 0.01 | 0.91 |
|  | Inverse variance weighted | 4 | -0.09 | 0.04 | 0.02 | 0.91 |
|  | Simple mode | 4 | -0.1 | 0.05 | 0.17 | 0.91 |
|  | Weighted mode | 4 | -0.1 | 0.05 | 0.12 | 0.91 |
| B4GALNT4 | MR Egger | 3 | -0.12 | 0.17 | 0.61 | 0.88 |
|  | Weighted median | 3 | -0.19 | 0.09 | 0.04 | 0.82 |
|  | Inverse variance weighted | 3 | -0.21 | 0.09 | 0.02 | 0.81 |
|  | Simple mode | 3 | -0.15 | 0.14 | 0.4 | 0.86 |
|  | Weighted mode | 3 | -0.18 | 0.1 | 0.21 | 0.83 |
| B4GALT3 | MR Egger | 10 | 0.19 | 0.16 | 0.26 | 1.21 |
|  | Weighted median | 10 | 0.19 | 0.07 | 0.01 | 1.2 |
|  | Inverse variance weighted | 10 | 0.14 | 0.06 | 0.02 | 1.15 |
|  | Simple mode | 10 | 0.17 | 0.11 | 0.17 | 1.19 |
|  | Weighted mode | 10 | 0.17 | 0.08 | 0.06 | 1.19 |
| BANK1 | MR Egger | 9 | 0.73 | 0.37 | 0.09 | 2.07 |
|  | Weighted median | 9 | 0.13 | 0.08 | 0.11 | 1.14 |
|  | Inverse variance weighted | 9 | 0.18 | 0.07 | 0.01 | 1.2 |
|  | Simple mode | 9 | 0.32 | 0.14 | 0.06 | 1.38 |
|  | Weighted mode | 9 | 0.11 | 0.14 | 0.46 | 1.11 |
| BLVRA | MR Egger | 4 | -0.13 | 0.1 | 0.29 | 0.87 |
|  | Weighted median | 4 | -0.08 | 0.03 | 0.02 | 0.92 |
|  | Inverse variance weighted | 4 | -0.07 | 0.03 | 0.02 | 0.93 |
|  | Simple mode | 4 | -0.07 | 0.05 | 0.24 | 0.93 |
|  | Weighted mode | 4 | -0.08 | 0.04 | 0.12 | 0.92 |
| BPGM | MR Egger | 6 | 0.13 | 0.16 | 0.47 | 1.14 |
|  | Weighted median | 6 | 0.12 | 0.07 | 0.07 | 1.12 |
|  | Inverse variance weighted | 6 | 0.12 | 0.05 | 0.02 | 1.13 |
|  | Simple mode | 6 | 0.1 | 0.09 | 0.36 | 1.1 |
|  | Weighted mode | 6 | 0.12 | 0.07 | 0.14 | 1.13 |
| BRAT1 | MR Egger | 3 | 0.29 | 0.15 | 0.31 | 1.33 |
|  | Weighted median | 3 | 0.18 | 0.06 | 0 | 1.2 |
|  | Inverse variance weighted | 3 | 0.18 | 0.06 | 0 | 1.19 |
|  | Simple mode | 3 | 0.04 | 0.13 | 0.79 | 1.04 |
|  | Weighted mode | 3 | 0.2 | 0.06 | 0.08 | 1.22 |
| BTN3A3 | MR Egger | 8 | 0.25 | 0.09 | 0.02 | 1.29 |
|  | Weighted median | 8 | 0.15 | 0.07 | 0.05 | 1.16 |
|  | Inverse variance weighted | 8 | 0.16 | 0.05 | 0 | 1.18 |
|  | Simple mode | 8 | 0.12 | 0.11 | 0.3 | 1.13 |
|  | Weighted mode | 8 | 0.15 | 0.09 | 0.14 | 1.17 |
| C12orf43 | MR Egger | 4 | 0.08 | 0.07 | 0.36 | 1.08 |
|  | Weighted median | 4 | 0.09 | 0.04 | 0.03 | 1.09 |
|  | Inverse variance weighted | 4 | 0.08 | 0.04 | 0.03 | 1.08 |
|  | Simple mode | 4 | 0.09 | 0.06 | 0.2 | 1.1 |
|  | Weighted mode | 4 | 0.09 | 0.04 | 0.12 | 1.09 |
| C16orf74 | MR Egger | 3 | 0.13 | 0.11 | 0.43 | 1.14 |
|  | Weighted median | 3 | 0.12 | 0.06 | 0.04 | 1.13 |
|  | Inverse variance weighted | 3 | 0.11 | 0.05 | 0.05 | 1.12 |
|  | Simple mode | 3 | 0.15 | 0.08 | 0.21 | 1.16 |
|  | Weighted mode | 3 | 0.12 | 0.06 | 0.17 | 1.13 |
| C1GALT1 | MR Egger | 6 | 0.19 | 0.1 | 0.15 | 1.21 |
|  | Weighted median | 6 | 0.07 | 0.04 | 0.06 | 1.08 |
|  | Inverse variance weighted | 6 | 0.07 | 0.03 | 0.04 | 1.07 |
|  | Simple mode | 6 | 0.08 | 0.06 | 0.24 | 1.09 |
|  | Weighted mode | 6 | 0.08 | 0.04 | 0.11 | 1.08 |
| C5 | MR Egger | 6 | 0.05 | 0.12 | 0.67 | 1.06 |
|  | Weighted median | 6 | 0.14 | 0.04 | 0 | 1.16 |
|  | Inverse variance weighted | 6 | 0.14 | 0.04 | 0 | 1.15 |
|  | Simple mode | 6 | 0.14 | 0.06 | 0.07 | 1.15 |
|  | Weighted mode | 6 | 0.14 | 0.04 | 0.02 | 1.15 |
| C7orf25 | MR Egger | 8 | 0.06 | 0.06 | 0.36 | 1.06 |
|  | Weighted median | 8 | 0.07 | 0.03 | 0.03 | 1.07 |
|  | Inverse variance weighted | 8 | 0.06 | 0.03 | 0.04 | 1.06 |
|  | Simple mode | 8 | 0.01 | 0.06 | 0.93 | 1.01 |
|  | Weighted mode | 8 | 0.06 | 0.03 | 0.11 | 1.06 |
| CACNA2D3 | MR Egger | 12 | 0.07 | 0.08 | 0.43 | 1.07 |
|  | Weighted median | 12 | 0.1 | 0.05 | 0.04 | 1.1 |
|  | Inverse variance weighted | 12 | 0.09 | 0.04 | 0.01 | 1.1 |
|  | Simple mode | 12 | 0.13 | 0.08 | 0.13 | 1.13 |
|  | Weighted mode | 12 | 0.09 | 0.05 | 0.07 | 1.1 |
| CARHSP1 | MR Egger | 9 | 0.16 | 0.05 | 0.01 | 1.18 |
|  | Weighted median | 9 | 0.13 | 0.03 | 0 | 1.14 |
|  | Inverse variance weighted | 9 | 0.11 | 0.03 | 0 | 1.12 |
|  | Simple mode | 9 | 0.09 | 0.08 | 0.25 | 1.1 |
|  | Weighted mode | 9 | 0.13 | 0.03 | 0 | 1.14 |
| CBFA2T3 | MR Egger | 3 | -0.23 | 0.19 | 0.44 | 0.79 |
|  | Weighted median | 3 | -0.19 | 0.09 | 0.03 | 0.82 |
|  | Inverse variance weighted | 3 | -0.18 | 0.08 | 0.03 | 0.83 |
|  | Simple mode | 3 | -0.19 | 0.12 | 0.25 | 0.83 |
|  | Weighted mode | 3 | -0.2 | 0.09 | 0.17 | 0.82 |
| CBS | MR Egger | 6 | 0.02 | 0.04 | 0.65 | 1.02 |
|  | Weighted median | 6 | 0.07 | 0.03 | 0.02 | 1.07 |
|  | Inverse variance weighted | 6 | 0.05 | 0.03 | 0.05 | 1.05 |
|  | Simple mode | 6 | 0.06 | 0.04 | 0.19 | 1.07 |
|  | Weighted mode | 6 | 0.06 | 0.03 | 0.11 | 1.06 |
| CCDC50 | MR Egger | 8 | 0.09 | 0.07 | 0.27 | 1.09 |
|  | Weighted median | 8 | 0.1 | 0.04 | 0.03 | 1.1 |
|  | Inverse variance weighted | 8 | 0.11 | 0.04 | 0 | 1.11 |
|  | Simple mode | 8 | 0.09 | 0.07 | 0.21 | 1.1 |
|  | Weighted mode | 8 | 0.1 | 0.04 | 0.06 | 1.1 |
| CCHCR1 | MR Egger | 3 | 0.08 | 0.3 | 0.83 | 1.09 |
|  | Weighted median | 3 | 0.12 | 0.04 | 0.01 | 1.13 |
|  | Inverse variance weighted | 3 | 0.12 | 0.04 | 0.01 | 1.12 |
|  | Simple mode | 3 | 0.17 | 0.07 | 0.14 | 1.19 |
|  | Weighted mode | 3 | 0.14 | 0.05 | 0.1 | 1.15 |
| CCSER2 | MR Egger | 10 | 0.03 | 0.05 | 0.54 | 1.03 |
|  | Weighted median | 10 | 0.06 | 0.03 | 0.07 | 1.06 |
|  | Inverse variance weighted | 10 | 0.07 | 0.03 | 0.01 | 1.07 |
|  | Simple mode | 10 | 0.05 | 0.05 | 0.27 | 1.06 |
|  | Weighted mode | 10 | 0.06 | 0.03 | 0.12 | 1.06 |
| CD33 | MR Egger | 5 | -0.1 | 0.06 | 0.2 | 0.9 |
|  | Weighted median | 5 | -0.09 | 0.05 | 0.08 | 0.91 |
|  | Inverse variance weighted | 5 | -0.1 | 0.05 | 0.05 | 0.91 |
|  | Simple mode | 5 | -0.07 | 0.08 | 0.46 | 0.94 |
|  | Weighted mode | 5 | -0.09 | 0.05 | 0.16 | 0.91 |
| CD40 | MR Egger | 3 | -0.1 | 0.05 | 0.26 | 0.9 |
|  | Weighted median | 3 | -0.1 | 0.03 | 0 | 0.91 |
|  | Inverse variance weighted | 3 | -0.1 | 0.03 | 0 | 0.91 |
|  | Simple mode | 3 | -0.11 | 0.04 | 0.13 | 0.9 |
|  | Weighted mode | 3 | -0.1 | 0.03 | 0.09 | 0.91 |
| CDH2 | MR Egger | 11 | -0.11 | 0.05 | 0.04 | 0.89 |
|  | Weighted median | 11 | -0.09 | 0.03 | 0.01 | 0.91 |
|  | Inverse variance weighted | 11 | -0.07 | 0.03 | 0.01 | 0.93 |
|  | Simple mode | 11 | -0.08 | 0.06 | 0.17 | 0.92 |
|  | Weighted mode | 11 | -0.09 | 0.03 | 0.02 | 0.92 |
| CEBPA | MR Egger | 3 | -0.28 | 0.3 | 0.52 | 0.75 |
|  | Weighted median | 3 | -0.22 | 0.09 | 0.02 | 0.8 |
|  | Inverse variance weighted | 3 | -0.2 | 0.09 | 0.02 | 0.82 |
|  | Simple mode | 3 | -0.23 | 0.12 | 0.2 | 0.8 |
|  | Weighted mode | 3 | -0.24 | 0.11 | 0.17 | 0.79 |
| CENPK | MR Egger | 6 | 0.1 | 0.05 | 0.15 | 1.1 |
|  | Weighted median | 6 | 0.06 | 0.03 | 0.04 | 1.06 |
|  | Inverse variance weighted | 6 | 0.06 | 0.03 | 0.03 | 1.06 |
|  | Simple mode | 6 | 0.02 | 0.05 | 0.66 | 1.02 |
|  | Weighted mode | 6 | 0.07 | 0.03 | 0.08 | 1.07 |
| CEP19 | MR Egger | 6 | -0.09 | 0.05 | 0.15 | 0.92 |
|  | Weighted median | 6 | -0.09 | 0.03 | 0.01 | 0.92 |
|  | Inverse variance weighted | 6 | -0.09 | 0.03 | 0.01 | 0.91 |
|  | Simple mode | 6 | -0.13 | 0.08 | 0.14 | 0.88 |
|  | Weighted mode | 6 | -0.08 | 0.04 | 0.06 | 0.92 |
| CEP63 | MR Egger | 8 | 0.03 | 0.06 | 0.63 | 1.03 |
|  | Weighted median | 8 | 0.07 | 0.03 | 0.03 | 1.07 |
|  | Inverse variance weighted | 8 | 0.07 | 0.03 | 0.02 | 1.07 |
|  | Simple mode | 8 | 0.08 | 0.07 | 0.29 | 1.08 |
|  | Weighted mode | 8 | 0.07 | 0.04 | 0.08 | 1.07 |
| CFAP410 | MR Egger | 5 | -0.04 | 0.04 | 0.36 | 0.96 |
|  | Weighted median | 5 | -0.06 | 0.03 | 0.06 | 0.94 |
|  | Inverse variance weighted | 5 | -0.06 | 0.03 | 0.03 | 0.95 |
|  | Simple mode | 5 | -0.09 | 0.04 | 0.1 | 0.91 |
|  | Weighted mode | 5 | -0.06 | 0.03 | 0.12 | 0.94 |
| CHPT1 | MR Egger | 7 | 0.09 | 0.05 | 0.14 | 1.09 |
|  | Weighted median | 7 | 0.08 | 0.03 | 0.02 | 1.08 |
|  | Inverse variance weighted | 7 | 0.07 | 0.03 | 0.01 | 1.08 |
|  | Simple mode | 7 | 0.11 | 0.06 | 0.1 | 1.12 |
|  | Weighted mode | 7 | 0.08 | 0.03 | 0.04 | 1.08 |
| CHST13 | MR Egger | 6 | 0.13 | 0.06 | 0.11 | 1.14 |
|  | Weighted median | 6 | 0.14 | 0.04 | 0 | 1.15 |
|  | Inverse variance weighted | 6 | 0.1 | 0.04 | 0.01 | 1.11 |
|  | Simple mode | 6 | 0.07 | 0.07 | 0.37 | 1.08 |
|  | Weighted mode | 6 | 0.12 | 0.04 | 0.03 | 1.13 |
| CHST15 | MR Egger | 4 | -0.3 | 0.14 | 0.16 | 0.74 |
|  | Weighted median | 4 | -0.21 | 0.07 | 0 | 0.81 |
|  | Inverse variance weighted | 4 | -0.18 | 0.07 | 0.01 | 0.84 |
|  | Simple mode | 4 | -0.19 | 0.12 | 0.2 | 0.82 |
|  | Weighted mode | 4 | -0.21 | 0.08 | 0.07 | 0.81 |
| CLEC4C | MR Egger | 8 | 0.02 | 0.06 | 0.74 | 1.02 |
|  | Weighted median | 8 | 0.06 | 0.04 | 0.12 | 1.06 |
|  | Inverse variance weighted | 8 | 0.08 | 0.04 | 0.04 | 1.08 |
|  | Simple mode | 8 | 0 | 0.07 | 1 | 1 |
|  | Weighted mode | 8 | 0.06 | 0.04 | 0.18 | 1.06 |
| CLIP4 | MR Egger | 3 | 0.16 | 0.17 | 0.52 | 1.18 |
|  | Weighted median | 3 | 0.15 | 0.06 | 0.01 | 1.17 |
|  | Inverse variance weighted | 3 | 0.15 | 0.06 | 0.01 | 1.16 |
|  | Simple mode | 3 | 0.18 | 0.09 | 0.16 | 1.2 |
|  | Weighted mode | 3 | 0.15 | 0.07 | 0.14 | 1.17 |
| CMBL | MR Egger | 8 | 0.06 | 0.03 | 0.1 | 1.07 |
|  | Weighted median | 8 | 0.05 | 0.03 | 0.08 | 1.05 |
|  | Inverse variance weighted | 8 | 0.05 | 0.02 | 0.04 | 1.05 |
|  | Simple mode | 8 | 0.04 | 0.04 | 0.43 | 1.04 |
|  | Weighted mode | 8 | 0.06 | 0.03 | 0.08 | 1.06 |
| COLGALT1 | MR Egger | 3 | -0.2 | 0.15 | 0.42 | 0.82 |
|  | Weighted median | 3 | -0.08 | 0.03 | 0.01 | 0.92 |
|  | Inverse variance weighted | 3 | -0.08 | 0.03 | 0.01 | 0.92 |
|  | Simple mode | 3 | -0.04 | 0.05 | 0.55 | 0.96 |
|  | Weighted mode | 3 | -0.09 | 0.03 | 0.12 | 0.91 |
| COMMD10 | MR Egger | 4 | 0.21 | 0.12 | 0.22 | 1.23 |
|  | Weighted median | 4 | 0.11 | 0.04 | 0.01 | 1.12 |
|  | Inverse variance weighted | 4 | 0.1 | 0.04 | 0.01 | 1.1 |
|  | Simple mode | 4 | 0.05 | 0.1 | 0.66 | 1.05 |
|  | Weighted mode | 4 | 0.11 | 0.04 | 0.06 | 1.12 |
| COX14 | MR Egger | 3 | 0.03 | 0.1 | 0.81 | 1.03 |
|  | Weighted median | 3 | 0.09 | 0.04 | 0.04 | 1.09 |
|  | Inverse variance weighted | 3 | 0.09 | 0.04 | 0.04 | 1.09 |
|  | Simple mode | 3 | 0.19 | 0.08 | 0.14 | 1.21 |
|  | Weighted mode | 3 | 0.08 | 0.05 | 0.23 | 1.08 |
| CTPS1 | MR Egger | 3 | -0.17 | 0.1 | 0.35 | 0.84 |
|  | Weighted median | 3 | -0.09 | 0.04 | 0.01 | 0.91 |
|  | Inverse variance weighted | 3 | -0.09 | 0.04 | 0.02 | 0.92 |
|  | Simple mode | 3 | -0.05 | 0.07 | 0.54 | 0.95 |
|  | Weighted mode | 3 | -0.09 | 0.04 | 0.12 | 0.91 |
| CYP1B1 | MR Egger | 12 | -0.06 | 0.04 | 0.13 | 0.94 |
|  | Weighted median | 12 | -0.06 | 0.03 | 0.07 | 0.94 |
|  | Inverse variance weighted | 12 | -0.06 | 0.03 | 0.01 | 0.94 |
|  | Simple mode | 12 | -0.18 | 0.06 | 0.01 | 0.84 |
|  | Weighted mode | 12 | -0.06 | 0.03 | 0.11 | 0.94 |
| CYP2S1 | MR Egger | 8 | -0.05 | 0.06 | 0.41 | 0.95 |
|  | Weighted median | 8 | -0.07 | 0.03 | 0.01 | 0.93 |
|  | Inverse variance weighted | 8 | -0.08 | 0.03 | 0.02 | 0.92 |
|  | Simple mode | 8 | -0.04 | 0.09 | 0.67 | 0.96 |
|  | Weighted mode | 8 | -0.07 | 0.03 | 0.06 | 0.93 |
| CYP2T1P | MR Egger | 4 | -0.13 | 0.1 | 0.3 | 0.88 |
|  | Weighted median | 4 | -0.1 | 0.03 | 0.01 | 0.91 |
|  | Inverse variance weighted | 4 | -0.11 | 0.04 | 0.01 | 0.9 |
|  | Simple mode | 4 | -0.12 | 0.07 | 0.19 | 0.88 |
|  | Weighted mode | 4 | -0.09 | 0.04 | 0.08 | 0.91 |
| CYTIP | MR Egger | 5 | 0.54 | 0.37 | 0.24 | 1.72 |
|  | Weighted median | 5 | 0.29 | 0.13 | 0.02 | 1.34 |
|  | Inverse variance weighted | 5 | 0.33 | 0.11 | 0 | 1.4 |
|  | Simple mode | 5 | 0.27 | 0.19 | 0.22 | 1.31 |
|  | Weighted mode | 5 | 0.26 | 0.16 | 0.18 | 1.29 |
| DCBLD1 | MR Egger | 4 | 0.14 | 0.1 | 0.29 | 1.15 |
|  | Weighted median | 4 | 0.18 | 0.04 | 0 | 1.19 |
|  | Inverse variance weighted | 4 | 0.18 | 0.04 | 0 | 1.2 |
|  | Simple mode | 4 | 0.19 | 0.06 | 0.05 | 1.21 |
|  | Weighted mode | 4 | 0.18 | 0.04 | 0.03 | 1.19 |
| DENND4B | MR Egger | 5 | 0.13 | 0.06 | 0.12 | 1.14 |
|  | Weighted median | 5 | 0.11 | 0.05 | 0.03 | 1.11 |
|  | Inverse variance weighted | 5 | 0.1 | 0.05 | 0.04 | 1.1 |
|  | Simple mode | 5 | 0.02 | 0.1 | 0.89 | 1.02 |
|  | Weighted mode | 5 | 0.12 | 0.05 | 0.1 | 1.12 |
| DIP2B | MR Egger | 5 | -0.19 | 0.08 | 0.11 | 0.83 |
|  | Weighted median | 5 | -0.17 | 0.05 | 0 | 0.84 |
|  | Inverse variance weighted | 5 | -0.16 | 0.04 | 0 | 0.85 |
|  | Simple mode | 5 | -0.19 | 0.07 | 0.05 | 0.83 |
|  | Weighted mode | 5 | -0.17 | 0.04 | 0.02 | 0.84 |
| DOCK2 | MR Egger | 5 | -0.11 | 0.09 | 0.31 | 0.9 |
|  | Weighted median | 5 | -0.11 | 0.05 | 0.05 | 0.9 |
|  | Inverse variance weighted | 5 | -0.1 | 0.05 | 0.02 | 0.9 |
|  | Simple mode | 5 | -0.08 | 0.07 | 0.32 | 0.93 |
|  | Weighted mode | 5 | -0.13 | 0.06 | 0.1 | 0.88 |
| DPEP3 | MR Egger | 10 | 0.07 | 0.07 | 0.36 | 1.07 |
|  | Weighted median | 10 | 0.07 | 0.04 | 0.11 | 1.07 |
|  | Inverse variance weighted | 10 | 0.07 | 0.03 | 0.04 | 1.07 |
|  | Simple mode | 10 | 0.07 | 0.07 | 0.35 | 1.07 |
|  | Weighted mode | 10 | 0.08 | 0.04 | 0.12 | 1.08 |
| DPF2 | MR Egger | 5 | 0.16 | 0.08 | 0.14 | 1.17 |
|  | Weighted median | 5 | 0.1 | 0.04 | 0.02 | 1.1 |
|  | Inverse variance weighted | 5 | 0.09 | 0.04 | 0.01 | 1.1 |
|  | Simple mode | 5 | 0.08 | 0.06 | 0.28 | 1.08 |
|  | Weighted mode | 5 | 0.1 | 0.04 | 0.08 | 1.1 |
| DRD4 | MR Egger | 3 | -0.19 | 0.14 | 0.41 | 0.83 |
|  | Weighted median | 3 | -0.18 | 0.08 | 0.03 | 0.84 |
|  | Inverse variance weighted | 3 | -0.16 | 0.07 | 0.03 | 0.85 |
|  | Simple mode | 3 | -0.24 | 0.11 | 0.17 | 0.79 |
|  | Weighted mode | 3 | -0.19 | 0.08 | 0.14 | 0.83 |
| DTHD1 | MR Egger | 4 | 0.13 | 0.15 | 0.45 | 1.14 |
|  | Weighted median | 4 | 0.12 | 0.07 | 0.09 | 1.13 |
|  | Inverse variance weighted | 4 | 0.13 | 0.06 | 0.04 | 1.14 |
|  | Simple mode | 4 | 0.14 | 0.11 | 0.3 | 1.15 |
|  | Weighted mode | 4 | 0.12 | 0.08 | 0.24 | 1.12 |
| ECI2 | MR Egger | 3 | -0.17 | 0.1 | 0.35 | 0.84 |
|  | Weighted median | 3 | -0.13 | 0.05 | 0.01 | 0.88 |
|  | Inverse variance weighted | 3 | -0.12 | 0.05 | 0.02 | 0.89 |
|  | Simple mode | 3 | -0.16 | 0.1 | 0.26 | 0.85 |
|  | Weighted mode | 3 | -0.13 | 0.05 | 0.13 | 0.88 |
| EGLN2 | MR Egger | 3 | 0.02 | 0.11 | 0.91 | 1.02 |
|  | Weighted median | 3 | 0.11 | 0.04 | 0 | 1.11 |
|  | Inverse variance weighted | 3 | 0.11 | 0.04 | 0 | 1.11 |
|  | Simple mode | 3 | 0.17 | 0.06 | 0.11 | 1.19 |
|  | Weighted mode | 3 | 0.09 | 0.04 | 0.12 | 1.1 |
| ENOSF1 | MR Egger | 4 | 0.16 | 0.1 | 0.23 | 1.18 |
|  | Weighted median | 4 | 0.09 | 0.04 | 0.03 | 1.1 |
|  | Inverse variance weighted | 4 | 0.09 | 0.04 | 0.02 | 1.09 |
|  | Simple mode | 4 | 0.08 | 0.06 | 0.29 | 1.08 |
|  | Weighted mode | 4 | 0.09 | 0.05 | 0.15 | 1.1 |
| EPHB4 | MR Egger | 8 | -0.12 | 0.13 | 0.38 | 0.89 |
|  | Weighted median | 8 | -0.11 | 0.04 | 0 | 0.89 |
|  | Inverse variance weighted | 8 | -0.08 | 0.03 | 0.01 | 0.92 |
|  | Simple mode | 8 | -0.04 | 0.07 | 0.56 | 0.96 |
|  | Weighted mode | 8 | -0.12 | 0.04 | 0.02 | 0.89 |
| EXTL2 | MR Egger | 3 | 0.14 | 0.09 | 0.37 | 1.15 |
|  | Weighted median | 3 | 0.08 | 0.04 | 0.03 | 1.08 |
|  | Inverse variance weighted | 3 | 0.07 | 0.03 | 0.03 | 1.08 |
|  | Simple mode | 3 | 0.09 | 0.05 | 0.2 | 1.1 |
|  | Weighted mode | 3 | 0.08 | 0.04 | 0.16 | 1.08 |
| FAAP24 | MR Egger | 5 | -0.09 | 0.07 | 0.29 | 0.91 |
|  | Weighted median | 5 | -0.09 | 0.04 | 0.02 | 0.92 |
|  | Inverse variance weighted | 5 | -0.09 | 0.04 | 0.01 | 0.91 |
|  | Simple mode | 5 | -0.15 | 0.07 | 0.09 | 0.86 |
|  | Weighted mode | 5 | -0.09 | 0.04 | 0.09 | 0.92 |
| FAHD2B | MR Egger | 3 | 0.21 | 0.29 | 0.6 | 1.24 |
|  | Weighted median | 3 | 0.11 | 0.05 | 0.04 | 1.12 |
|  | Inverse variance weighted | 3 | 0.11 | 0.05 | 0.03 | 1.11 |
|  | Simple mode | 3 | 0.11 | 0.06 | 0.22 | 1.11 |
|  | Weighted mode | 3 | 0.11 | 0.06 | 0.2 | 1.12 |
| FAM167A | MR Egger | 4 | 0 | 0.1 | 0.97 | 1 |
|  | Weighted median | 4 | -0.06 | 0.03 | 0.08 | 0.94 |
|  | Inverse variance weighted | 4 | -0.06 | 0.03 | 0.05 | 0.94 |
|  | Simple mode | 4 | -0.06 | 0.05 | 0.28 | 0.94 |
|  | Weighted mode | 4 | -0.06 | 0.03 | 0.15 | 0.94 |
| FAM174A | MR Egger | 7 | -0.05 | 0.07 | 0.51 | 0.95 |
|  | Weighted median | 7 | -0.11 | 0.04 | 0.01 | 0.9 |
|  | Inverse variance weighted | 7 | -0.11 | 0.04 | 0 | 0.89 |
|  | Simple mode | 7 | -0.11 | 0.07 | 0.17 | 0.9 |
|  | Weighted mode | 7 | -0.11 | 0.04 | 0.04 | 0.9 |
| FAM241A | MR Egger | 5 | -0.15 | 0.1 | 0.2 | 0.86 |
|  | Weighted median | 5 | -0.12 | 0.04 | 0 | 0.88 |
|  | Inverse variance weighted | 5 | -0.11 | 0.04 | 0.01 | 0.89 |
|  | Simple mode | 5 | -0.09 | 0.11 | 0.46 | 0.92 |
|  | Weighted mode | 5 | -0.13 | 0.04 | 0.04 | 0.88 |
| FLJ12825 | MR Egger | 3 | 0.05 | 0.12 | 0.74 | 1.06 |
|  | Weighted median | 3 | 0.09 | 0.04 | 0.03 | 1.09 |
|  | Inverse variance weighted | 3 | 0.09 | 0.04 | 0.03 | 1.09 |
|  | Simple mode | 3 | 0.07 | 0.07 | 0.41 | 1.08 |
|  | Weighted mode | 3 | 0.08 | 0.04 | 0.17 | 1.09 |
| FLT1 | MR Egger | 5 | -0.22 | 0.09 | 0.09 | 0.8 |
|  | Weighted median | 5 | -0.17 | 0.06 | 0 | 0.85 |
|  | Inverse variance weighted | 5 | -0.14 | 0.05 | 0.01 | 0.87 |
|  | Simple mode | 5 | -0.15 | 0.1 | 0.2 | 0.86 |
|  | Weighted mode | 5 | -0.17 | 0.06 | 0.04 | 0.84 |
| FOXO4 | MR Egger | 3 | -0.03 | 0.71 | 0.98 | 0.97 |
|  | Weighted median | 3 | -0.21 | 0.12 | 0.07 | 0.81 |
|  | Inverse variance weighted | 3 | -0.23 | 0.11 | 0.03 | 0.79 |
|  | Simple mode | 3 | -0.2 | 0.15 | 0.32 | 0.82 |
|  | Weighted mode | 3 | -0.2 | 0.15 | 0.3 | 0.82 |
| FYN | MR Egger | 3 | -0.04 | 0.35 | 0.93 | 0.96 |
|  | Weighted median | 3 | -0.15 | 0.1 | 0.12 | 0.86 |
|  | Inverse variance weighted | 3 | -0.2 | 0.09 | 0.03 | 0.82 |
|  | Simple mode | 3 | -0.16 | 0.14 | 0.39 | 0.86 |
|  | Weighted mode | 3 | -0.14 | 0.11 | 0.34 | 0.87 |
| GBAP1 | MR Egger | 5 | -0.51 | 0.55 | 0.42 | 0.6 |
|  | Weighted median | 5 | -0.09 | 0.04 | 0.03 | 0.91 |
|  | Inverse variance weighted | 5 | -0.08 | 0.04 | 0.05 | 0.93 |
|  | Simple mode | 5 | -0.1 | 0.07 | 0.22 | 0.91 |
|  | Weighted mode | 5 | -0.09 | 0.04 | 0.09 | 0.91 |
| GLIPR2 | MR Egger | 7 | 0.02 | 0.09 | 0.87 | 1.02 |
|  | Weighted median | 7 | 0.08 | 0.05 | 0.14 | 1.08 |
|  | Inverse variance weighted | 7 | 0.09 | 0.04 | 0.04 | 1.09 |
|  | Simple mode | 7 | 0.02 | 0.09 | 0.8 | 1.02 |
|  | Weighted mode | 7 | 0.07 | 0.06 | 0.24 | 1.08 |
| GPSM2 | MR Egger | 3 | 0.21 | 0.25 | 0.55 | 1.24 |
|  | Weighted median | 3 | 0.25 | 0.1 | 0.01 | 1.28 |
|  | Inverse variance weighted | 3 | 0.25 | 0.09 | 0.01 | 1.28 |
|  | Simple mode | 3 | 0.35 | 0.15 | 0.14 | 1.42 |
|  | Weighted mode | 3 | 0.19 | 0.12 | 0.26 | 1.2 |
| GRAMD4 | MR Egger | 3 | -0.01 | 0.11 | 0.96 | 0.99 |
|  | Weighted median | 3 | -0.09 | 0.04 | 0.04 | 0.92 |
|  | Inverse variance weighted | 3 | -0.09 | 0.04 | 0.01 | 0.91 |
|  | Simple mode | 3 | -0.13 | 0.07 | 0.22 | 0.88 |
|  | Weighted mode | 3 | -0.07 | 0.04 | 0.23 | 0.93 |
| GSN | MR Egger | 7 | 0.08 | 0.05 | 0.17 | 1.09 |
|  | Weighted median | 7 | 0.07 | 0.03 | 0.03 | 1.07 |
|  | Inverse variance weighted | 7 | 0.08 | 0.03 | 0.01 | 1.08 |
|  | Simple mode | 7 | 0 | 0.08 | 0.98 | 1 |
|  | Weighted mode | 7 | 0.07 | 0.03 | 0.08 | 1.08 |
| GSTO1 | MR Egger | 3 | 0.15 | 0.15 | 0.49 | 1.16 |
|  | Weighted median | 3 | 0.11 | 0.05 | 0.01 | 1.12 |
|  | Inverse variance weighted | 3 | 0.11 | 0.04 | 0.01 | 1.12 |
|  | Simple mode | 3 | 0.08 | 0.07 | 0.4 | 1.08 |
|  | Weighted mode | 3 | 0.15 | 0.06 | 0.12 | 1.16 |
| GTF3A | MR Egger | 4 | 0 | 0.12 | 0.98 | 1 |
|  | Weighted median | 4 | 0.09 | 0.04 | 0.03 | 1.09 |
|  | Inverse variance weighted | 4 | 0.11 | 0.05 | 0.03 | 1.12 |
|  | Simple mode | 4 | 0.12 | 0.12 | 0.4 | 1.12 |
|  | Weighted mode | 4 | 0.09 | 0.04 | 0.14 | 1.09 |
| HADH | MR Egger | 3 | 0.31 | 0.2 | 0.37 | 1.36 |
|  | Weighted median | 3 | 0.23 | 0.07 | 0 | 1.26 |
|  | Inverse variance weighted | 3 | 0.17 | 0.08 | 0.03 | 1.19 |
|  | Simple mode | 3 | 0.23 | 0.12 | 0.21 | 1.25 |
|  | Weighted mode | 3 | 0.24 | 0.08 | 0.09 | 1.27 |
| HBD | MR Egger | 7 | 0.3 | 0.21 | 0.22 | 1.35 |
|  | Weighted median | 7 | 0.18 | 0.09 | 0.04 | 1.2 |
|  | Inverse variance weighted | 7 | 0.21 | 0.07 | 0 | 1.23 |
|  | Simple mode | 7 | 0.22 | 0.12 | 0.12 | 1.24 |
|  | Weighted mode | 7 | 0.19 | 0.1 | 0.09 | 1.21 |
| HBG1 | MR Egger | 5 | -0.02 | 0.06 | 0.74 | 0.98 |
|  | Weighted median | 5 | -0.03 | 0.04 | 0.34 | 0.97 |
|  | Inverse variance weighted | 5 | -0.06 | 0.03 | 0.04 | 0.94 |
|  | Simple mode | 5 | -0.06 | 0.04 | 0.25 | 0.95 |
|  | Weighted mode | 5 | -0.05 | 0.04 | 0.26 | 0.95 |
| HCP5 | MR Egger | 4 | -0.41 | 0.22 | 0.21 | 0.66 |
|  | Weighted median | 4 | -0.15 | 0.12 | 0.2 | 0.86 |
|  | Inverse variance weighted | 4 | -0.23 | 0.11 | 0.03 | 0.8 |
|  | Simple mode | 4 | -0.05 | 0.2 | 0.83 | 0.95 |
|  | Weighted mode | 4 | -0.42 | 0.13 | 0.05 | 0.66 |
| HLA-C | MR Egger | 7 | 0.2 | 0.08 | 0.06 | 1.22 |
|  | Weighted median | 7 | 0.11 | 0.04 | 0 | 1.11 |
|  | Inverse variance weighted | 7 | 0.11 | 0.03 | 0 | 1.11 |
|  | Simple mode | 7 | 0.08 | 0.05 | 0.17 | 1.08 |
|  | Weighted mode | 7 | 0.09 | 0.04 | 0.05 | 1.1 |
| HOXA5 | MR Egger | 3 | 0.07 | 0.14 | 0.68 | 1.08 |
|  | Weighted median | 3 | 0.14 | 0.04 | 0 | 1.15 |
|  | Inverse variance weighted | 3 | 0.14 | 0.04 | 0 | 1.15 |
|  | Simple mode | 3 | 0.18 | 0.06 | 0.1 | 1.2 |
|  | Weighted mode | 3 | 0.11 | 0.05 | 0.13 | 1.12 |
| HPS4 | MR Egger | 3 | 0.09 | 0.1 | 0.54 | 1.09 |
|  | Weighted median | 3 | 0.13 | 0.08 | 0.13 | 1.13 |
|  | Inverse variance weighted | 3 | 0.15 | 0.08 | 0.05 | 1.16 |
|  | Simple mode | 3 | 0.12 | 0.1 | 0.35 | 1.13 |
|  | Weighted mode | 3 | 0.13 | 0.09 | 0.31 | 1.13 |
| ICAM3 | MR Egger | 3 | -0.11 | 0.07 | 0.35 | 0.9 |
|  | Weighted median | 3 | -0.09 | 0.04 | 0.01 | 0.91 |
|  | Inverse variance weighted | 3 | -0.09 | 0.04 | 0.01 | 0.91 |
|  | Simple mode | 3 | -0.12 | 0.07 | 0.22 | 0.89 |
|  | Weighted mode | 3 | -0.09 | 0.04 | 0.12 | 0.91 |
| IER3 | MR Egger | 8 | 0.14 | 0.08 | 0.13 | 1.15 |
|  | Weighted median | 8 | 0.11 | 0.04 | 0.01 | 1.11 |
|  | Inverse variance weighted | 8 | 0.11 | 0.04 | 0.01 | 1.11 |
|  | Simple mode | 8 | 0.11 | 0.08 | 0.23 | 1.11 |
|  | Weighted mode | 8 | 0.11 | 0.04 | 0.04 | 1.11 |
| IGSF9B | MR Egger | 3 | -0.26 | 0.13 | 0.29 | 0.77 |
|  | Weighted median | 3 | -0.17 | 0.06 | 0.01 | 0.84 |
|  | Inverse variance weighted | 3 | -0.15 | 0.06 | 0.01 | 0.86 |
|  | Simple mode | 3 | -0.19 | 0.08 | 0.13 | 0.83 |
|  | Weighted mode | 3 | -0.17 | 0.07 | 0.13 | 0.84 |
| INSC | MR Egger | 4 | 0.11 | 0.13 | 0.5 | 1.12 |
|  | Weighted median | 4 | 0.12 | 0.06 | 0.05 | 1.12 |
|  | Inverse variance weighted | 4 | 0.12 | 0.05 | 0.03 | 1.12 |
|  | Simple mode | 4 | 0.09 | 0.08 | 0.36 | 1.09 |
|  | Weighted mode | 4 | 0.12 | 0.06 | 0.15 | 1.12 |
| IPO8 | MR Egger | 5 | 0.03 | 0.05 | 0.62 | 1.03 |
|  | Weighted median | 5 | 0.05 | 0.03 | 0.11 | 1.05 |
|  | Inverse variance weighted | 5 | 0.06 | 0.03 | 0.03 | 1.06 |
|  | Simple mode | 5 | 0.05 | 0.05 | 0.36 | 1.05 |
|  | Weighted mode | 5 | 0.05 | 0.03 | 0.24 | 1.05 |
| IREB2 | MR Egger | 4 | -0.43 | 0.07 | 0.03 | 0.65 |
|  | Weighted median | 4 | -0.35 | 0.03 | 0 | 0.71 |
|  | Inverse variance weighted | 4 | -0.35 | 0.04 | 0 | 0.71 |
|  | Simple mode | 4 | -0.37 | 0.1 | 0.04 | 0.69 |
|  | Weighted mode | 4 | -0.36 | 0.03 | 0 | 0.7 |
| IRX3 | MR Egger | 8 | 0.14 | 0.05 | 0.03 | 1.15 |
|  | Weighted median | 8 | 0.09 | 0.03 | 0 | 1.1 |
|  | Inverse variance weighted | 8 | 0.09 | 0.03 | 0 | 1.09 |
|  | Simple mode | 8 | 0.03 | 0.09 | 0.72 | 1.03 |
|  | Weighted mode | 8 | 0.1 | 0.03 | 0.03 | 1.1 |
| JARID2 | MR Egger | 7 | -0.04 | 0.05 | 0.43 | 0.96 |
|  | Weighted median | 7 | -0.07 | 0.04 | 0.04 | 0.93 |
|  | Inverse variance weighted | 7 | -0.09 | 0.03 | 0.01 | 0.92 |
|  | Simple mode | 7 | -0.06 | 0.08 | 0.49 | 0.94 |
|  | Weighted mode | 7 | -0.07 | 0.03 | 0.08 | 0.93 |
| KANSL1-AS1 | MR Egger | 5 | -0.05 | 0.04 | 0.32 | 0.96 |
|  | Weighted median | 5 | -0.07 | 0.02 | 0 | 0.93 |
|  | Inverse variance weighted | 5 | -0.07 | 0.02 | 0 | 0.93 |
|  | Simple mode | 5 | -0.11 | 0.04 | 0.05 | 0.89 |
|  | Weighted mode | 5 | -0.07 | 0.02 | 0.04 | 0.93 |
| KAT2B | MR Egger | 5 | -0.12 | 0.08 | 0.23 | 0.89 |
|  | Weighted median | 5 | -0.1 | 0.05 | 0.06 | 0.91 |
|  | Inverse variance weighted | 5 | -0.11 | 0.05 | 0.03 | 0.9 |
|  | Simple mode | 5 | -0.1 | 0.09 | 0.32 | 0.91 |
|  | Weighted mode | 5 | -0.1 | 0.05 | 0.14 | 0.91 |
| KCNMB1 | MR Egger | 5 | -0.07 | 0.06 | 0.37 | 0.93 |
|  | Weighted median | 5 | -0.1 | 0.04 | 0.01 | 0.91 |
|  | Inverse variance weighted | 5 | -0.09 | 0.04 | 0.01 | 0.91 |
|  | Simple mode | 5 | -0.08 | 0.06 | 0.28 | 0.93 |
|  | Weighted mode | 5 | -0.1 | 0.04 | 0.08 | 0.91 |
| KIAA2013 | MR Egger | 3 | 0.03 | 0.06 | 0.74 | 1.03 |
|  | Weighted median | 3 | 0.1 | 0.04 | 0.01 | 1.11 |
|  | Inverse variance weighted | 3 | 0.1 | 0.05 | 0.02 | 1.11 |
|  | Simple mode | 3 | 0.34 | 0.16 | 0.17 | 1.41 |
|  | Weighted mode | 3 | 0.09 | 0.04 | 0.16 | 1.09 |
| KLHL6 | MR Egger | 4 | 0.06 | 0.1 | 0.58 | 1.07 |
|  | Weighted median | 4 | 0.11 | 0.06 | 0.06 | 1.11 |
|  | Inverse variance weighted | 4 | 0.12 | 0.05 | 0.03 | 1.12 |
|  | Simple mode | 4 | 0.13 | 0.08 | 0.17 | 1.14 |
|  | Weighted mode | 4 | 0.1 | 0.06 | 0.2 | 1.11 |
| L1TD1 | MR Egger | 6 | 0.04 | 0.04 | 0.34 | 1.04 |
|  | Weighted median | 6 | 0.06 | 0.03 | 0.07 | 1.06 |
|  | Inverse variance weighted | 6 | 0.07 | 0.03 | 0.02 | 1.07 |
|  | Simple mode | 6 | 0.14 | 0.07 | 0.12 | 1.15 |
|  | Weighted mode | 6 | 0.04 | 0.04 | 0.27 | 1.04 |
| LAMTOR4 | MR Egger | 3 | -0.28 | 0.27 | 0.48 | 0.75 |
|  | Weighted median | 3 | -0.1 | 0.05 | 0.02 | 0.9 |
|  | Inverse variance weighted | 3 | -0.1 | 0.05 | 0.02 | 0.9 |
|  | Simple mode | 3 | -0.09 | 0.08 | 0.37 | 0.92 |
|  | Weighted mode | 3 | -0.1 | 0.05 | 0.18 | 0.9 |
| LARGE2 | MR Egger | 3 | 0.11 | 0.24 | 0.74 | 1.11 |
|  | Weighted median | 3 | 0.24 | 0.12 | 0.04 | 1.27 |
|  | Inverse variance weighted | 3 | 0.26 | 0.1 | 0.01 | 1.3 |
|  | Simple mode | 3 | 0.22 | 0.15 | 0.28 | 1.25 |
|  | Weighted mode | 3 | 0.24 | 0.14 | 0.24 | 1.27 |
| LGMN | MR Egger | 3 | 0.12 | 0.11 | 0.46 | 1.13 |
|  | Weighted median | 3 | 0.13 | 0.05 | 0.01 | 1.14 |
|  | Inverse variance weighted | 3 | 0.14 | 0.05 | 0.01 | 1.15 |
|  | Simple mode | 3 | 0.11 | 0.09 | 0.35 | 1.11 |
|  | Weighted mode | 3 | 0.13 | 0.06 | 0.14 | 1.14 |
| LIMCH1 | MR Egger | 3 | 0.05 | 0.42 | 0.92 | 1.05 |
|  | Weighted median | 3 | 0.1 | 0.08 | 0.22 | 1.1 |
|  | Inverse variance weighted | 3 | 0.15 | 0.07 | 0.04 | 1.16 |
|  | Simple mode | 3 | 0.1 | 0.11 | 0.48 | 1.1 |
|  | Weighted mode | 3 | 0.09 | 0.09 | 0.43 | 1.09 |
| LINC00243 | MR Egger | 4 | 0.15 | 0.07 | 0.17 | 1.17 |
|  | Weighted median | 4 | 0.2 | 0.04 | 0 | 1.22 |
|  | Inverse variance weighted | 4 | 0.21 | 0.04 | 0 | 1.23 |
|  | Simple mode | 4 | 0.26 | 0.08 | 0.04 | 1.29 |
|  | Weighted mode | 4 | 0.19 | 0.05 | 0.03 | 1.21 |
| LINC00665 | MR Egger | 4 | 0.07 | 0.1 | 0.52 | 1.08 |
|  | Weighted median | 4 | 0.13 | 0.04 | 0 | 1.14 |
|  | Inverse variance weighted | 4 | 0.13 | 0.04 | 0 | 1.14 |
|  | Simple mode | 4 | 0.19 | 0.06 | 0.05 | 1.2 |
|  | Weighted mode | 4 | 0.12 | 0.05 | 0.08 | 1.13 |
| LINC01150 | MR Egger | 3 | 0.1 | 0.19 | 0.7 | 1.1 |
|  | Weighted median | 3 | 0.27 | 0.08 | 0 | 1.32 |
|  | Inverse variance weighted | 3 | 0.28 | 0.08 | 0 | 1.33 |
|  | Simple mode | 3 | 0.42 | 0.14 | 0.1 | 1.52 |
|  | Weighted mode | 3 | 0.25 | 0.09 | 0.11 | 1.28 |
| LINC02210 | MR Egger | 5 | -0.05 | 0.05 | 0.37 | 0.95 |
|  | Weighted median | 5 | -0.07 | 0.02 | 0 | 0.94 |
|  | Inverse variance weighted | 5 | -0.06 | 0.02 | 0.02 | 0.95 |
|  | Simple mode | 5 | -0.1 | 0.03 | 0.04 | 0.9 |
|  | Weighted mode | 5 | -0.07 | 0.02 | 0.04 | 0.93 |
| LINC02723 | MR Egger | 5 | 0.1 | 0.1 | 0.43 | 1.1 |
|  | Weighted median | 5 | 0.07 | 0.04 | 0.06 | 1.08 |
|  | Inverse variance weighted | 5 | 0.08 | 0.04 | 0.02 | 1.09 |
|  | Simple mode | 5 | 0.13 | 0.06 | 0.1 | 1.14 |
|  | Weighted mode | 5 | 0.07 | 0.04 | 0.13 | 1.08 |
| LLPH-DT | MR Egger | 3 | -0.06 | 0.15 | 0.75 | 0.94 |
|  | Weighted median | 3 | -0.07 | 0.03 | 0.03 | 0.93 |
|  | Inverse variance weighted | 3 | -0.07 | 0.03 | 0.03 | 0.93 |
|  | Simple mode | 3 | -0.07 | 0.05 | 0.29 | 0.93 |
|  | Weighted mode | 3 | -0.07 | 0.04 | 0.18 | 0.93 |
| LOXL2 | MR Egger | 3 | -0.02 | 0.14 | 0.91 | 0.98 |
|  | Weighted median | 3 | -0.09 | 0.05 | 0.06 | 0.92 |
|  | Inverse variance weighted | 3 | -0.09 | 0.04 | 0.04 | 0.91 |
|  | Simple mode | 3 | -0.09 | 0.07 | 0.32 | 0.91 |
|  | Weighted mode | 3 | -0.08 | 0.04 | 0.2 | 0.92 |
| LPIN1 | MR Egger | 5 | -0.03 | 0.06 | 0.63 | 0.97 |
|  | Weighted median | 5 | -0.07 | 0.04 | 0.05 | 0.93 |
|  | Inverse variance weighted | 5 | -0.08 | 0.03 | 0.02 | 0.93 |
|  | Simple mode | 5 | -0.02 | 0.06 | 0.7 | 0.98 |
|  | Weighted mode | 5 | -0.09 | 0.04 | 0.08 | 0.92 |
| LRRC27 | MR Egger | 3 | 0.05 | 0.08 | 0.65 | 1.05 |
|  | Weighted median | 3 | 0.08 | 0.05 | 0.1 | 1.08 |
|  | Inverse variance weighted | 3 | 0.09 | 0.05 | 0.05 | 1.1 |
|  | Simple mode | 3 | 0.08 | 0.08 | 0.42 | 1.09 |
|  | Weighted mode | 3 | 0.08 | 0.05 | 0.26 | 1.08 |
| LYAR | MR Egger | 4 | 0.1 | 0.08 | 0.33 | 1.11 |
|  | Weighted median | 4 | 0.08 | 0.04 | 0.04 | 1.08 |
|  | Inverse variance weighted | 4 | 0.08 | 0.04 | 0.04 | 1.08 |
|  | Simple mode | 4 | 0.06 | 0.07 | 0.47 | 1.06 |
|  | Weighted mode | 4 | 0.08 | 0.04 | 0.15 | 1.09 |
| MAP3K20 | MR Egger | 5 | 0.03 | 0.06 | 0.65 | 1.03 |
|  | Weighted median | 5 | 0.1 | 0.04 | 0.01 | 1.1 |
|  | Inverse variance weighted | 5 | 0.08 | 0.03 | 0.01 | 1.08 |
|  | Simple mode | 5 | 0.05 | 0.06 | 0.47 | 1.05 |
|  | Weighted mode | 5 | 0.08 | 0.04 | 0.1 | 1.09 |
| MAPK8IP1P2 | MR Egger | 3 | -0.2 | 0.09 | 0.27 | 0.82 |
|  | Weighted median | 3 | -0.07 | 0.02 | 0 | 0.93 |
|  | Inverse variance weighted | 3 | -0.07 | 0.03 | 0.01 | 0.93 |
|  | Simple mode | 3 | -0.07 | 0.03 | 0.1 | 0.93 |
|  | Weighted mode | 3 | -0.07 | 0.02 | 0.09 | 0.93 |
| MBTPS1 | MR Egger | 3 | 0.13 | 0.06 | 0.27 | 1.14 |
|  | Weighted median | 3 | 0.08 | 0.03 | 0.02 | 1.08 |
|  | Inverse variance weighted | 3 | 0.07 | 0.03 | 0.02 | 1.08 |
|  | Simple mode | 3 | 0.07 | 0.04 | 0.25 | 1.07 |
|  | Weighted mode | 3 | 0.08 | 0.04 | 0.16 | 1.08 |
| MCOLN2 | MR Egger | 5 | -0.05 | 0.07 | 0.51 | 0.95 |
|  | Weighted median | 5 | -0.08 | 0.04 | 0.02 | 0.92 |
|  | Inverse variance weighted | 5 | -0.08 | 0.03 | 0.01 | 0.92 |
|  | Simple mode | 5 | -0.07 | 0.05 | 0.22 | 0.93 |
|  | Weighted mode | 5 | -0.08 | 0.04 | 0.12 | 0.93 |
| MCOLN3 | MR Egger | 4 | -0.17 | 0.14 | 0.35 | 0.84 |
|  | Weighted median | 4 | -0.18 | 0.07 | 0.01 | 0.83 |
|  | Inverse variance weighted | 4 | -0.19 | 0.06 | 0 | 0.83 |
|  | Simple mode | 4 | -0.21 | 0.12 | 0.18 | 0.81 |
|  | Weighted mode | 4 | -0.18 | 0.07 | 0.07 | 0.83 |
| MDFIC | MR Egger | 4 | -0.1 | 0.17 | 0.62 | 0.91 |
|  | Weighted median | 4 | -0.18 | 0.09 | 0.04 | 0.83 |
|  | Inverse variance weighted | 4 | -0.21 | 0.08 | 0.01 | 0.81 |
|  | Simple mode | 4 | -0.19 | 0.13 | 0.24 | 0.83 |
|  | Weighted mode | 4 | -0.18 | 0.1 | 0.16 | 0.84 |
| METTL27 | MR Egger | 4 | -0.07 | 0.09 | 0.54 | 0.94 |
|  | Weighted median | 4 | -0.07 | 0.03 | 0.02 | 0.93 |
|  | Inverse variance weighted | 4 | -0.07 | 0.03 | 0.02 | 0.94 |
|  | Simple mode | 4 | -0.08 | 0.04 | 0.18 | 0.93 |
|  | Weighted mode | 4 | -0.07 | 0.03 | 0.1 | 0.93 |
| METTL7A | MR Egger | 3 | -0.01 | 0.09 | 0.91 | 0.99 |
|  | Weighted median | 3 | -0.11 | 0.04 | 0 | 0.89 |
|  | Inverse variance weighted | 3 | -0.12 | 0.06 | 0.04 | 0.89 |
|  | Simple mode | 3 | -0.31 | 0.16 | 0.2 | 0.73 |
|  | Weighted mode | 3 | -0.09 | 0.04 | 0.15 | 0.91 |
| MFN2 | MR Egger | 6 | 0.01 | 0.05 | 0.85 | 1.01 |
|  | Weighted median | 6 | 0.08 | 0.03 | 0.01 | 1.08 |
|  | Inverse variance weighted | 6 | 0.07 | 0.03 | 0.01 | 1.08 |
|  | Simple mode | 6 | 0.09 | 0.05 | 0.13 | 1.1 |
|  | Weighted mode | 6 | 0.08 | 0.03 | 0.06 | 1.08 |
| MFSD10 | MR Egger | 3 | 0.18 | 0.19 | 0.51 | 1.2 |
|  | Weighted median | 3 | 0.17 | 0.07 | 0.02 | 1.18 |
|  | Inverse variance weighted | 3 | 0.17 | 0.07 | 0.01 | 1.18 |
|  | Simple mode | 3 | 0.14 | 0.11 | 0.33 | 1.15 |
|  | Weighted mode | 3 | 0.16 | 0.08 | 0.17 | 1.17 |
| MFSD9 | MR Egger | 4 | -0.08 | 0.05 | 0.24 | 0.92 |
|  | Weighted median | 4 | -0.07 | 0.02 | 0.01 | 0.93 |
|  | Inverse variance weighted | 4 | -0.07 | 0.02 | 0 | 0.93 |
|  | Simple mode | 4 | -0.09 | 0.05 | 0.16 | 0.91 |
|  | Weighted mode | 4 | -0.07 | 0.03 | 0.07 | 0.93 |
| MKKS | MR Egger | 4 | 0.17 | 0.07 | 0.15 | 1.18 |
|  | Weighted median | 4 | 0.1 | 0.04 | 0 | 1.11 |
|  | Inverse variance weighted | 4 | 0.09 | 0.03 | 0.01 | 1.1 |
|  | Simple mode | 4 | 0.09 | 0.05 | 0.17 | 1.09 |
|  | Weighted mode | 4 | 0.1 | 0.04 | 0.08 | 1.11 |
| MPHOSPH6 | MR Egger | 5 | 0.07 | 0.05 | 0.23 | 1.07 |
|  | Weighted median | 5 | 0.12 | 0.03 | 0 | 1.12 |
|  | Inverse variance weighted | 5 | 0.13 | 0.04 | 0 | 1.13 |
|  | Simple mode | 5 | 0.14 | 0.11 | 0.25 | 1.15 |
|  | Weighted mode | 5 | 0.12 | 0.03 | 0.01 | 1.12 |
| MPZL3 | MR Egger | 3 | -0.63 | 0.6 | 0.48 | 0.53 |
|  | Weighted median | 3 | -0.25 | 0.04 | 0 | 0.78 |
|  | Inverse variance weighted | 3 | -0.25 | 0.07 | 0 | 0.78 |
|  | Simple mode | 3 | -0.32 | 0.11 | 0.1 | 0.72 |
|  | Weighted mode | 3 | -0.26 | 0.04 | 0.03 | 0.77 |
| MRE11 | MR Egger | 5 | 0.04 | 0.05 | 0.46 | 1.04 |
|  | Weighted median | 5 | 0.05 | 0.03 | 0.06 | 1.05 |
|  | Inverse variance weighted | 5 | 0.06 | 0.02 | 0.03 | 1.06 |
|  | Simple mode | 5 | 0.06 | 0.04 | 0.22 | 1.07 |
|  | Weighted mode | 5 | 0.05 | 0.03 | 0.14 | 1.05 |
| MRPL42 | MR Egger | 3 | -0.07 | 0.15 | 0.72 | 0.93 |
|  | Weighted median | 3 | -0.09 | 0.03 | 0.01 | 0.92 |
|  | Inverse variance weighted | 3 | -0.09 | 0.03 | 0.01 | 0.92 |
|  | Simple mode | 3 | -0.1 | 0.07 | 0.3 | 0.91 |
|  | Weighted mode | 3 | -0.09 | 0.04 | 0.13 | 0.92 |
| MST1L | MR Egger | 3 | 0.1 | 0.15 | 0.62 | 1.11 |
|  | Weighted median | 3 | 0.1 | 0.04 | 0.01 | 1.11 |
|  | Inverse variance weighted | 3 | 0.1 | 0.04 | 0.01 | 1.1 |
|  | Simple mode | 3 | 0.1 | 0.05 | 0.18 | 1.11 |
|  | Weighted mode | 3 | 0.1 | 0.04 | 0.13 | 1.11 |
| N4BP2 | MR Egger | 4 | -0.1 | 0.08 | 0.32 | 0.9 |
|  | Weighted median | 4 | -0.08 | 0.04 | 0.03 | 0.92 |
|  | Inverse variance weighted | 4 | -0.09 | 0.04 | 0.02 | 0.91 |
|  | Simple mode | 4 | -0.08 | 0.07 | 0.35 | 0.92 |
|  | Weighted mode | 4 | -0.08 | 0.04 | 0.15 | 0.92 |
| NAPSB | MR Egger | 4 | -0.03 | 0.04 | 0.49 | 0.97 |
|  | Weighted median | 4 | -0.05 | 0.03 | 0.05 | 0.95 |
|  | Inverse variance weighted | 4 | -0.05 | 0.02 | 0.03 | 0.95 |
|  | Simple mode | 4 | -0.05 | 0.03 | 0.2 | 0.95 |
|  | Weighted mode | 4 | -0.05 | 0.03 | 0.18 | 0.95 |
| NCKAP1L | MR Egger | 3 | -0.06 | 0.15 | 0.75 | 0.94 |
|  | Weighted median | 3 | -0.11 | 0.06 | 0.1 | 0.9 |
|  | Inverse variance weighted | 3 | -0.13 | 0.06 | 0.04 | 0.88 |
|  | Simple mode | 3 | -0.1 | 0.11 | 0.48 | 0.91 |
|  | Weighted mode | 3 | -0.1 | 0.07 | 0.25 | 0.9 |
| NCOA3 | MR Egger | 4 | -0.13 | 0.1 | 0.34 | 0.88 |
|  | Weighted median | 4 | -0.09 | 0.05 | 0.06 | 0.91 |
|  | Inverse variance weighted | 4 | -0.1 | 0.04 | 0.02 | 0.91 |
|  | Simple mode | 4 | -0.16 | 0.07 | 0.1 | 0.85 |
|  | Weighted mode | 4 | -0.08 | 0.05 | 0.22 | 0.92 |
| NEFH | MR Egger | 3 | 0.04 | 0.07 | 0.64 | 1.04 |
|  | Weighted median | 3 | 0.1 | 0.04 | 0.03 | 1.1 |
|  | Inverse variance weighted | 3 | 0.1 | 0.04 | 0.02 | 1.11 |
|  | Simple mode | 3 | 0.23 | 0.11 | 0.16 | 1.26 |
|  | Weighted mode | 3 | 0.09 | 0.04 | 0.18 | 1.09 |
| NFKBIA | MR Egger | 4 | -0.16 | 0.1 | 0.25 | 0.85 |
|  | Weighted median | 4 | -0.11 | 0.05 | 0.02 | 0.89 |
|  | Inverse variance weighted | 4 | -0.11 | 0.05 | 0.03 | 0.89 |
|  | Simple mode | 4 | 0 | 0.1 | 0.98 | 1 |
|  | Weighted mode | 4 | -0.13 | 0.05 | 0.08 | 0.88 |
| NIBAN2 | MR Egger | 3 | -0.19 | 0.12 | 0.37 | 0.83 |
|  | Weighted median | 3 | -0.18 | 0.06 | 0.01 | 0.84 |
|  | Inverse variance weighted | 3 | -0.18 | 0.06 | 0 | 0.84 |
|  | Simple mode | 3 | -0.21 | 0.09 | 0.16 | 0.81 |
|  | Weighted mode | 3 | -0.2 | 0.07 | 0.11 | 0.82 |
| NME8 | MR Egger | 6 | -0.1 | 0.07 | 0.21 | 0.91 |
|  | Weighted median | 6 | -0.07 | 0.04 | 0.07 | 0.94 |
|  | Inverse variance weighted | 6 | -0.07 | 0.03 | 0.05 | 0.93 |
|  | Simple mode | 6 | -0.05 | 0.08 | 0.56 | 0.95 |
|  | Weighted mode | 6 | -0.07 | 0.04 | 0.12 | 0.94 |
| NOP14-AS1 | MR Egger | 3 | -0.27 | 0.14 | 0.31 | 0.76 |
|  | Weighted median | 3 | -0.14 | 0.06 | 0.03 | 0.87 |
|  | Inverse variance weighted | 3 | -0.12 | 0.06 | 0.04 | 0.88 |
|  | Simple mode | 3 | -0.14 | 0.08 | 0.23 | 0.87 |
|  | Weighted mode | 3 | -0.14 | 0.08 | 0.21 | 0.87 |
| NOSIP | MR Egger | 5 | 0.12 | 0.1 | 0.29 | 1.13 |
|  | Weighted median | 5 | 0.2 | 0.06 | 0 | 1.22 |
|  | Inverse variance weighted | 5 | 0.18 | 0.06 | 0 | 1.2 |
|  | Simple mode | 5 | 0.24 | 0.12 | 0.11 | 1.27 |
|  | Weighted mode | 5 | 0.19 | 0.07 | 0.05 | 1.21 |
| NOTCH4 | MR Egger | 3 | 0.16 | 0.13 | 0.44 | 1.17 |
|  | Weighted median | 3 | 0.2 | 0.06 | 0 | 1.22 |
|  | Inverse variance weighted | 3 | 0.2 | 0.06 | 0 | 1.23 |
|  | Simple mode | 3 | 0.16 | 0.09 | 0.23 | 1.17 |
|  | Weighted mode | 3 | 0.19 | 0.06 | 0.1 | 1.21 |
| NPHP3 | MR Egger | 12 | 0.08 | 0.06 | 0.22 | 1.08 |
|  | Weighted median | 12 | 0.07 | 0.03 | 0.03 | 1.08 |
|  | Inverse variance weighted | 12 | 0.07 | 0.03 | 0.02 | 1.07 |
|  | Simple mode | 12 | 0.07 | 0.05 | 0.24 | 1.07 |
|  | Weighted mode | 12 | 0.08 | 0.03 | 0.04 | 1.08 |
| NPRL3 | MR Egger | 7 | -0.07 | 0.06 | 0.32 | 0.94 |
|  | Weighted median | 7 | -0.08 | 0.04 | 0.05 | 0.93 |
|  | Inverse variance weighted | 7 | -0.07 | 0.03 | 0.03 | 0.93 |
|  | Simple mode | 7 | -0.14 | 0.07 | 0.08 | 0.87 |
|  | Weighted mode | 7 | -0.08 | 0.05 | 0.14 | 0.93 |
| NT5C3B | MR Egger | 12 | -0.05 | 0.03 | 0.13 | 0.95 |
|  | Weighted median | 12 | -0.06 | 0.02 | 0.01 | 0.94 |
|  | Inverse variance weighted | 12 | -0.04 | 0.02 | 0.04 | 0.96 |
|  | Simple mode | 12 | -0.03 | 0.04 | 0.47 | 0.97 |
|  | Weighted mode | 12 | -0.06 | 0.02 | 0.03 | 0.94 |
| NT5DC3 | MR Egger | 5 | 0.06 | 0.04 | 0.21 | 1.07 |
|  | Weighted median | 5 | 0.05 | 0.02 | 0.03 | 1.05 |
|  | Inverse variance weighted | 5 | 0.05 | 0.02 | 0.01 | 1.05 |
|  | Simple mode | 5 | 0.05 | 0.04 | 0.32 | 1.05 |
|  | Weighted mode | 5 | 0.04 | 0.02 | 0.13 | 1.04 |
| NUDT16L2P | MR Egger | 3 | -0.15 | 0.08 | 0.31 | 0.86 |
|  | Weighted median | 3 | -0.08 | 0.04 | 0.03 | 0.92 |
|  | Inverse variance weighted | 3 | -0.08 | 0.04 | 0.04 | 0.92 |
|  | Simple mode | 3 | -0.07 | 0.06 | 0.39 | 0.94 |
|  | Weighted mode | 3 | -0.09 | 0.04 | 0.19 | 0.92 |
| OSBPL3 | MR Egger | 4 | -0.11 | 0.12 | 0.47 | 0.9 |
|  | Weighted median | 4 | -0.11 | 0.04 | 0.01 | 0.89 |
|  | Inverse variance weighted | 4 | -0.1 | 0.05 | 0.03 | 0.91 |
|  | Simple mode | 4 | -0.13 | 0.08 | 0.22 | 0.88 |
|  | Weighted mode | 4 | -0.11 | 0.04 | 0.07 | 0.9 |
| OSGIN1 | MR Egger | 3 | 0.08 | 0.1 | 0.57 | 1.08 |
|  | Weighted median | 3 | 0.1 | 0.05 | 0.03 | 1.11 |
|  | Inverse variance weighted | 3 | 0.1 | 0.05 | 0.04 | 1.1 |
|  | Simple mode | 3 | 0.13 | 0.08 | 0.24 | 1.14 |
|  | Weighted mode | 3 | 0.1 | 0.05 | 0.17 | 1.11 |
| PAPLN | MR Egger | 3 | 0.12 | 0.13 | 0.53 | 1.13 |
|  | Weighted median | 3 | 0.14 | 0.08 | 0.07 | 1.16 |
|  | Inverse variance weighted | 3 | 0.15 | 0.08 | 0.04 | 1.17 |
|  | Simple mode | 3 | 0.16 | 0.12 | 0.32 | 1.17 |
|  | Weighted mode | 3 | 0.14 | 0.08 | 0.24 | 1.15 |
| PDCD6P1 | MR Egger | 3 | 0.07 | 0.1 | 0.61 | 1.07 |
|  | Weighted median | 3 | 0.13 | 0.04 | 0 | 1.14 |
|  | Inverse variance weighted | 3 | 0.14 | 0.04 | 0 | 1.15 |
|  | Simple mode | 3 | 0.13 | 0.07 | 0.21 | 1.14 |
|  | Weighted mode | 3 | 0.12 | 0.05 | 0.12 | 1.13 |
| PDLIM1P4 | MR Egger | 3 | 0.1 | 0.11 | 0.54 | 1.1 |
|  | Weighted median | 3 | 0.14 | 0.05 | 0.01 | 1.15 |
|  | Inverse variance weighted | 3 | 0.15 | 0.05 | 0 | 1.16 |
|  | Simple mode | 3 | 0.12 | 0.07 | 0.22 | 1.13 |
|  | Weighted mode | 3 | 0.14 | 0.05 | 0.12 | 1.15 |
| PHF19 | MR Egger | 5 | 0.12 | 0.11 | 0.34 | 1.13 |
|  | Weighted median | 5 | 0.1 | 0.04 | 0.02 | 1.11 |
|  | Inverse variance weighted | 5 | 0.09 | 0.04 | 0.04 | 1.1 |
|  | Simple mode | 5 | 0.15 | 0.1 | 0.22 | 1.16 |
|  | Weighted mode | 5 | 0.11 | 0.04 | 0.07 | 1.11 |
| POLR1B | MR Egger | 4 | 0.1 | 0.05 | 0.2 | 1.1 |
|  | Weighted median | 4 | 0.08 | 0.04 | 0.06 | 1.08 |
|  | Inverse variance weighted | 4 | 0.07 | 0.04 | 0.05 | 1.08 |
|  | Simple mode | 4 | 0.04 | 0.06 | 0.55 | 1.04 |
|  | Weighted mode | 4 | 0.07 | 0.04 | 0.18 | 1.08 |
| POLR1H | MR Egger | 3 | -0.09 | 0.06 | 0.39 | 0.91 |
|  | Weighted median | 3 | -0.17 | 0.05 | 0 | 0.84 |
|  | Inverse variance weighted | 3 | -0.19 | 0.07 | 0.01 | 0.83 |
|  | Simple mode | 3 | -0.2 | 0.09 | 0.16 | 0.82 |
|  | Weighted mode | 3 | -0.16 | 0.05 | 0.07 | 0.85 |
| POLR3K | MR Egger | 3 | 0.12 | 0.08 | 0.39 | 1.12 |
|  | Weighted median | 3 | 0.11 | 0.06 | 0.04 | 1.12 |
|  | Inverse variance weighted | 3 | 0.12 | 0.05 | 0.03 | 1.13 |
|  | Simple mode | 3 | 0.11 | 0.1 | 0.36 | 1.12 |
|  | Weighted mode | 3 | 0.11 | 0.06 | 0.19 | 1.12 |
| POU2AF1 | MR Egger | 6 | 0.36 | 0.82 | 0.68 | 1.44 |
|  | Weighted median | 6 | 0.28 | 0.13 | 0.03 | 1.32 |
|  | Inverse variance weighted | 6 | 0.25 | 0.12 | 0.03 | 1.28 |
|  | Simple mode | 6 | 0.42 | 0.22 | 0.12 | 1.52 |
|  | Weighted mode | 6 | 0.31 | 0.2 | 0.18 | 1.36 |
| POU5F1 | MR Egger | 3 | -0.24 | 0.15 | 0.34 | 0.78 |
|  | Weighted median | 3 | -0.12 | 0.04 | 0 | 0.89 |
|  | Inverse variance weighted | 3 | -0.12 | 0.04 | 0 | 0.89 |
|  | Simple mode | 3 | -0.15 | 0.08 | 0.22 | 0.86 |
|  | Weighted mode | 3 | -0.13 | 0.04 | 0.08 | 0.88 |
| PPT1 | MR Egger | 7 | 0.05 | 0.03 | 0.21 | 1.05 |
|  | Weighted median | 7 | 0.05 | 0.03 | 0.05 | 1.05 |
|  | Inverse variance weighted | 7 | 0.06 | 0.02 | 0.01 | 1.06 |
|  | Simple mode | 7 | 0.04 | 0.04 | 0.3 | 1.05 |
|  | Weighted mode | 7 | 0.05 | 0.02 | 0.08 | 1.05 |
| PRMT5 | MR Egger | 3 | -0.14 | 0.12 | 0.46 | 0.87 |
|  | Weighted median | 3 | -0.09 | 0.04 | 0.01 | 0.91 |
|  | Inverse variance weighted | 3 | -0.09 | 0.04 | 0.01 | 0.91 |
|  | Simple mode | 3 | -0.06 | 0.07 | 0.48 | 0.94 |
|  | Weighted mode | 3 | -0.09 | 0.04 | 0.14 | 0.91 |
| PRPF31 | MR Egger | 3 | -0.11 | 0.05 | 0.29 | 0.9 |
|  | Weighted median | 3 | -0.08 | 0.04 | 0.03 | 0.92 |
|  | Inverse variance weighted | 3 | -0.08 | 0.04 | 0.03 | 0.92 |
|  | Simple mode | 3 | -0.12 | 0.07 | 0.23 | 0.89 |
|  | Weighted mode | 3 | -0.08 | 0.03 | 0.14 | 0.92 |
| PSORS1C3 | MR Egger | 4 | -0.06 | 0.11 | 0.65 | 0.94 |
|  | Weighted median | 4 | -0.09 | 0.04 | 0.02 | 0.91 |
|  | Inverse variance weighted | 4 | -0.1 | 0.05 | 0.05 | 0.91 |
|  | Simple mode | 4 | -0.14 | 0.07 | 0.12 | 0.87 |
|  | Weighted mode | 4 | -0.09 | 0.04 | 0.12 | 0.92 |
| PTDSS1 | MR Egger | 3 | -0.02 | 0.11 | 0.89 | 0.98 |
|  | Weighted median | 3 | -0.14 | 0.06 | 0.02 | 0.87 |
|  | Inverse variance weighted | 3 | -0.14 | 0.07 | 0.05 | 0.87 |
|  | Simple mode | 3 | -0.36 | 0.17 | 0.17 | 0.69 |
|  | Weighted mode | 3 | -0.1 | 0.07 | 0.3 | 0.91 |
| PTGFR | MR Egger | 3 | -0.44 | 0.25 | 0.33 | 0.64 |
|  | Weighted median | 3 | -0.24 | 0.07 | 0 | 0.79 |
|  | Inverse variance weighted | 3 | -0.22 | 0.1 | 0.03 | 0.8 |
|  | Simple mode | 3 | -0.14 | 0.18 | 0.52 | 0.87 |
|  | Weighted mode | 3 | -0.29 | 0.07 | 0.06 | 0.75 |
| PYROXD2 | MR Egger | 3 | 0.22 | 0.27 | 0.56 | 1.25 |
|  | Weighted median | 3 | 0.19 | 0.09 | 0.03 | 1.21 |
|  | Inverse variance weighted | 3 | 0.19 | 0.08 | 0.02 | 1.21 |
|  | Simple mode | 3 | 0.22 | 0.12 | 0.22 | 1.24 |
|  | Weighted mode | 3 | 0.19 | 0.1 | 0.2 | 1.21 |
| RAB8A | MR Egger | 3 | -0.09 | 0.07 | 0.39 | 0.91 |
|  | Weighted median | 3 | -0.09 | 0.04 | 0.04 | 0.91 |
|  | Inverse variance weighted | 3 | -0.09 | 0.04 | 0.04 | 0.91 |
|  | Simple mode | 3 | -0.11 | 0.08 | 0.33 | 0.9 |
|  | Weighted mode | 3 | -0.09 | 0.04 | 0.17 | 0.91 |
| RCBTB1 | MR Egger | 5 | 0.1 | 0.06 | 0.18 | 1.1 |
|  | Weighted median | 5 | 0.07 | 0.03 | 0.03 | 1.08 |
|  | Inverse variance weighted | 5 | 0.06 | 0.03 | 0.03 | 1.06 |
|  | Simple mode | 5 | 0.08 | 0.04 | 0.12 | 1.08 |
|  | Weighted mode | 5 | 0.07 | 0.03 | 0.12 | 1.07 |
| RCC1 | MR Egger | 3 | 0.13 | 0.13 | 0.51 | 1.13 |
|  | Weighted median | 3 | 0.14 | 0.06 | 0.03 | 1.15 |
|  | Inverse variance weighted | 3 | 0.13 | 0.06 | 0.03 | 1.14 |
|  | Simple mode | 3 | 0.19 | 0.1 | 0.2 | 1.21 |
|  | Weighted mode | 3 | 0.15 | 0.07 | 0.17 | 1.16 |
| RFTN1 | MR Egger | 3 | -0.15 | 0.25 | 0.64 | 0.86 |
|  | Weighted median | 3 | -0.11 | 0.05 | 0.03 | 0.89 |
|  | Inverse variance weighted | 3 | -0.12 | 0.05 | 0.01 | 0.89 |
|  | Simple mode | 3 | -0.1 | 0.06 | 0.23 | 0.9 |
|  | Weighted mode | 3 | -0.1 | 0.06 | 0.23 | 0.9 |
| RHOBTB3 | MR Egger | 3 | 0.15 | 0.2 | 0.58 | 1.16 |
|  | Weighted median | 3 | 0.2 | 0.11 | 0.08 | 1.22 |
|  | Inverse variance weighted | 3 | 0.2 | 0.1 | 0.04 | 1.23 |
|  | Simple mode | 3 | 0.21 | 0.14 | 0.27 | 1.24 |
|  | Weighted mode | 3 | 0.18 | 0.14 | 0.32 | 1.2 |
| RHOU | MR Egger | 5 | -0.01 | 0.07 | 0.9 | 0.99 |
|  | Weighted median | 5 | -0.1 | 0.04 | 0.02 | 0.91 |
|  | Inverse variance weighted | 5 | -0.09 | 0.04 | 0.01 | 0.92 |
|  | Simple mode | 5 | -0.09 | 0.06 | 0.18 | 0.91 |
|  | Weighted mode | 5 | -0.08 | 0.04 | 0.12 | 0.92 |
| RHPN2 | MR Egger | 3 | -0.13 | 0.12 | 0.47 | 0.88 |
|  | Weighted median | 3 | -0.15 | 0.06 | 0.01 | 0.86 |
|  | Inverse variance weighted | 3 | -0.15 | 0.06 | 0.01 | 0.86 |
|  | Simple mode | 3 | -0.13 | 0.08 | 0.24 | 0.88 |
|  | Weighted mode | 3 | -0.13 | 0.06 | 0.17 | 0.88 |
| RILP | MR Egger | 3 | 0 | 0.16 | 0.98 | 1 |
|  | Weighted median | 3 | -0.16 | 0.08 | 0.03 | 0.85 |
|  | Inverse variance weighted | 3 | -0.16 | 0.07 | 0.02 | 0.85 |
|  | Simple mode | 3 | -0.31 | 0.14 | 0.15 | 0.73 |
|  | Weighted mode | 3 | -0.12 | 0.08 | 0.3 | 0.89 |
| RINL | MR Egger | 3 | -0.07 | 0.14 | 0.69 | 0.93 |
|  | Weighted median | 3 | -0.19 | 0.06 | 0 | 0.83 |
|  | Inverse variance weighted | 3 | -0.19 | 0.06 | 0 | 0.82 |
|  | Simple mode | 3 | -0.14 | 0.1 | 0.31 | 0.87 |
|  | Weighted mode | 3 | -0.18 | 0.06 | 0.1 | 0.84 |
| RNASET2 | MR Egger | 5 | 0.1 | 0.04 | 0.08 | 1.11 |
|  | Weighted median | 5 | 0.07 | 0.02 | 0 | 1.08 |
|  | Inverse variance weighted | 5 | 0.07 | 0.02 | 0 | 1.08 |
|  | Simple mode | 5 | 0.02 | 0.04 | 0.64 | 1.02 |
|  | Weighted mode | 5 | 0.07 | 0.02 | 0.02 | 1.08 |
| RNF24 | MR Egger | 4 | -0.13 | 0.08 | 0.25 | 0.88 |
|  | Weighted median | 4 | -0.09 | 0.05 | 0.04 | 0.91 |
|  | Inverse variance weighted | 4 | -0.09 | 0.04 | 0.04 | 0.92 |
|  | Simple mode | 4 | -0.09 | 0.06 | 0.24 | 0.91 |
|  | Weighted mode | 4 | -0.1 | 0.05 | 0.13 | 0.91 |
| RNF39 | MR Egger | 3 | -0.27 | 0.21 | 0.43 | 0.76 |
|  | Weighted median | 3 | -0.22 | 0.07 | 0 | 0.8 |
|  | Inverse variance weighted | 3 | -0.21 | 0.08 | 0.01 | 0.81 |
|  | Simple mode | 3 | -0.29 | 0.11 | 0.12 | 0.75 |
|  | Weighted mode | 3 | -0.24 | 0.08 | 0.1 | 0.78 |
| RPA1 | MR Egger | 6 | -0.11 | 0.05 | 0.1 | 0.9 |
|  | Weighted median | 6 | -0.09 | 0.04 | 0.01 | 0.91 |
|  | Inverse variance weighted | 6 | -0.08 | 0.04 | 0.03 | 0.93 |
|  | Simple mode | 6 | -0.11 | 0.08 | 0.23 | 0.89 |
|  | Weighted mode | 6 | -0.1 | 0.04 | 0.06 | 0.91 |
| RTP4 | MR Egger | 4 | 0.18 | 0.08 | 0.14 | 1.2 |
|  | Weighted median | 4 | 0.11 | 0.04 | 0.01 | 1.12 |
|  | Inverse variance weighted | 4 | 0.11 | 0.04 | 0.01 | 1.11 |
|  | Simple mode | 4 | 0.14 | 0.07 | 0.13 | 1.15 |
|  | Weighted mode | 4 | 0.11 | 0.05 | 0.1 | 1.12 |
| RUNX2 | MR Egger | 5 | -0.11 | 0.09 | 0.32 | 0.9 |
|  | Weighted median | 5 | -0.1 | 0.04 | 0.01 | 0.91 |
|  | Inverse variance weighted | 5 | -0.08 | 0.04 | 0.05 | 0.92 |
|  | Simple mode | 5 | -0.13 | 0.05 | 0.06 | 0.88 |
|  | Weighted mode | 5 | -0.08 | 0.04 | 0.13 | 0.93 |
| S100A11 | MR Egger | 4 | 0.14 | 0.09 | 0.27 | 1.15 |
|  | Weighted median | 4 | 0.17 | 0.07 | 0.02 | 1.18 |
|  | Inverse variance weighted | 4 | 0.19 | 0.07 | 0 | 1.2 |
|  | Simple mode | 4 | 0.34 | 0.12 | 0.07 | 1.41 |
|  | Weighted mode | 4 | 0.16 | 0.07 | 0.11 | 1.17 |
| S1PR3 | MR Egger | 7 | -0.07 | 0.08 | 0.42 | 0.93 |
|  | Weighted median | 7 | -0.09 | 0.05 | 0.05 | 0.91 |
|  | Inverse variance weighted | 7 | -0.1 | 0.04 | 0.01 | 0.9 |
|  | Simple mode | 7 | -0.2 | 0.08 | 0.05 | 0.82 |
|  | Weighted mode | 7 | -0.1 | 0.05 | 0.09 | 0.91 |
| SARS2 | MR Egger | 5 | 0.07 | 0.08 | 0.39 | 1.08 |
|  | Weighted median | 5 | 0.08 | 0.04 | 0.03 | 1.08 |
|  | Inverse variance weighted | 5 | 0.07 | 0.03 | 0.04 | 1.07 |
|  | Simple mode | 5 | 0.08 | 0.05 | 0.18 | 1.08 |
|  | Weighted mode | 5 | 0.07 | 0.03 | 0.1 | 1.07 |
| SCO1 | MR Egger | 3 | -0.12 | 0.08 | 0.39 | 0.89 |
|  | Weighted median | 3 | -0.08 | 0.03 | 0.01 | 0.93 |
|  | Inverse variance weighted | 3 | -0.08 | 0.03 | 0.01 | 0.93 |
|  | Simple mode | 3 | -0.09 | 0.09 | 0.39 | 0.91 |
|  | Weighted mode | 3 | -0.08 | 0.03 | 0.13 | 0.92 |
| SDHAP1 | MR Egger | 4 | 0.15 | 0.16 | 0.45 | 1.16 |
|  | Weighted median | 4 | 0.13 | 0.05 | 0.01 | 1.14 |
|  | Inverse variance weighted | 4 | 0.14 | 0.06 | 0.02 | 1.15 |
|  | Simple mode | 4 | 0.12 | 0.13 | 0.39 | 1.13 |
|  | Weighted mode | 4 | 0.11 | 0.05 | 0.12 | 1.12 |
| SENP6 | MR Egger | 4 | 0.12 | 0.56 | 0.85 | 1.12 |
|  | Weighted median | 4 | 0.07 | 0.03 | 0.03 | 1.07 |
|  | Inverse variance weighted | 4 | 0.07 | 0.03 | 0.02 | 1.07 |
|  | Simple mode | 4 | 0.06 | 0.04 | 0.24 | 1.07 |
|  | Weighted mode | 4 | 0.07 | 0.03 | 0.14 | 1.07 |
| SENP7 | MR Egger | 7 | 0.11 | 0.08 | 0.21 | 1.11 |
|  | Weighted median | 7 | 0.06 | 0.02 | 0 | 1.06 |
|  | Inverse variance weighted | 7 | 0.06 | 0.02 | 0.01 | 1.06 |
|  | Simple mode | 7 | 0.08 | 0.05 | 0.15 | 1.08 |
|  | Weighted mode | 7 | 0.06 | 0.02 | 0.04 | 1.06 |
| SERPINF2 | MR Egger | 3 | 0.03 | 0.07 | 0.77 | 1.03 |
|  | Weighted median | 3 | 0.1 | 0.04 | 0.03 | 1.1 |
|  | Inverse variance weighted | 3 | 0.1 | 0.05 | 0.02 | 1.11 |
|  | Simple mode | 3 | 0.13 | 0.09 | 0.28 | 1.14 |
|  | Weighted mode | 3 | 0.09 | 0.05 | 0.2 | 1.09 |
| SETMAR | MR Egger | 3 | -0.15 | 0.11 | 0.42 | 0.86 |
|  | Weighted median | 3 | -0.14 | 0.06 | 0.03 | 0.87 |
|  | Inverse variance weighted | 3 | -0.14 | 0.06 | 0.02 | 0.87 |
|  | Simple mode | 3 | -0.1 | 0.08 | 0.34 | 0.9 |
|  | Weighted mode | 3 | -0.12 | 0.08 | 0.24 | 0.88 |
| SGTB | MR Egger | 3 | 0.26 | 0.12 | 0.28 | 1.3 |
|  | Weighted median | 3 | 0.09 | 0.04 | 0.03 | 1.09 |
|  | Inverse variance weighted | 3 | 0.09 | 0.04 | 0.02 | 1.09 |
|  | Simple mode | 3 | 0.05 | 0.06 | 0.47 | 1.05 |
|  | Weighted mode | 3 | 0.08 | 0.05 | 0.24 | 1.08 |
| SIPA1L2 | MR Egger | 7 | 0.12 | 0.05 | 0.05 | 1.13 |
|  | Weighted median | 7 | 0.09 | 0.03 | 0 | 1.09 |
|  | Inverse variance weighted | 7 | 0.08 | 0.03 | 0.01 | 1.08 |
|  | Simple mode | 7 | 0.06 | 0.06 | 0.36 | 1.07 |
|  | Weighted mode | 7 | 0.09 | 0.03 | 0.04 | 1.1 |
| SKAP2 | MR Egger | 7 | 0.05 | 0.21 | 0.83 | 1.05 |
|  | Weighted median | 7 | 0.07 | 0.02 | 0 | 1.07 |
|  | Inverse variance weighted | 7 | 0.06 | 0.02 | 0 | 1.06 |
|  | Simple mode | 7 | 0.09 | 0.04 | 0.06 | 1.09 |
|  | Weighted mode | 7 | 0.07 | 0.02 | 0.02 | 1.07 |
| SLC12A2-DT | MR Egger | 3 | -0.04 | 0.06 | 0.67 | 0.97 |
|  | Weighted median | 3 | -0.07 | 0.03 | 0.04 | 0.93 |
|  | Inverse variance weighted | 3 | -0.08 | 0.03 | 0.02 | 0.93 |
|  | Simple mode | 3 | -0.05 | 0.06 | 0.45 | 0.95 |
|  | Weighted mode | 3 | -0.07 | 0.04 | 0.2 | 0.93 |
| SLC18A1 | MR Egger | 3 | -0.08 | 0.11 | 0.59 | 0.92 |
|  | Weighted median | 3 | -0.08 | 0.04 | 0.04 | 0.93 |
|  | Inverse variance weighted | 3 | -0.08 | 0.04 | 0.04 | 0.93 |
|  | Simple mode | 3 | -0.06 | 0.07 | 0.43 | 0.94 |
|  | Weighted mode | 3 | -0.08 | 0.04 | 0.19 | 0.93 |
| SLC23A3 | MR Egger | 6 | 0.04 | 0.05 | 0.51 | 1.04 |
|  | Weighted median | 6 | 0.09 | 0.04 | 0.03 | 1.09 |
|  | Inverse variance weighted | 6 | 0.09 | 0.04 | 0.02 | 1.09 |
|  | Simple mode | 6 | 0.05 | 0.09 | 0.57 | 1.05 |
|  | Weighted mode | 6 | 0.09 | 0.04 | 0.07 | 1.09 |
| SLC25A29 | MR Egger | 5 | 0.05 | 0.06 | 0.48 | 1.05 |
|  | Weighted median | 5 | 0.06 | 0.04 | 0.08 | 1.07 |
|  | Inverse variance weighted | 5 | 0.07 | 0.03 | 0.02 | 1.08 |
|  | Simple mode | 5 | 0.07 | 0.05 | 0.21 | 1.07 |
|  | Weighted mode | 5 | 0.07 | 0.04 | 0.13 | 1.07 |
| SLC43A2 | MR Egger | 4 | -0.02 | 0.41 | 0.96 | 0.98 |
|  | Weighted median | 4 | -0.25 | 0.12 | 0.04 | 0.78 |
|  | Inverse variance weighted | 4 | -0.2 | 0.1 | 0.04 | 0.82 |
|  | Simple mode | 4 | -0.25 | 0.17 | 0.23 | 0.78 |
|  | Weighted mode | 4 | -0.27 | 0.13 | 0.13 | 0.77 |
| SNCA | MR Egger | 9 | 0.15 | 0.07 | 0.07 | 1.16 |
|  | Weighted median | 9 | 0.12 | 0.04 | 0.01 | 1.13 |
|  | Inverse variance weighted | 9 | 0.1 | 0.04 | 0.01 | 1.11 |
|  | Simple mode | 9 | 0.16 | 0.09 | 0.12 | 1.18 |
|  | Weighted mode | 9 | 0.13 | 0.05 | 0.03 | 1.14 |
| SNIP1 | MR Egger | 4 | 0.13 | 0.07 | 0.22 | 1.14 |
|  | Weighted median | 4 | 0.11 | 0.04 | 0.01 | 1.11 |
|  | Inverse variance weighted | 4 | 0.1 | 0.04 | 0.02 | 1.1 |
|  | Simple mode | 4 | 0.07 | 0.09 | 0.5 | 1.07 |
|  | Weighted mode | 4 | 0.12 | 0.04 | 0.07 | 1.13 |
| SNN | MR Egger | 3 | 0.52 | 0.41 | 0.43 | 1.68 |
|  | Weighted median | 3 | 0.43 | 0.15 | 0.01 | 1.54 |
|  | Inverse variance weighted | 3 | 0.38 | 0.16 | 0.01 | 1.46 |
|  | Simple mode | 3 | 0.54 | 0.23 | 0.15 | 1.72 |
|  | Weighted mode | 3 | 0.52 | 0.22 | 0.14 | 1.69 |
| SNRNP25 | MR Egger | 3 | -0.1 | 0.08 | 0.41 | 0.9 |
|  | Weighted median | 3 | -0.1 | 0.05 | 0.05 | 0.9 |
|  | Inverse variance weighted | 3 | -0.11 | 0.05 | 0.03 | 0.9 |
|  | Simple mode | 3 | -0.1 | 0.09 | 0.37 | 0.91 |
|  | Weighted mode | 3 | -0.1 | 0.05 | 0.19 | 0.9 |
| SNTB1 | MR Egger | 4 | 0.28 | 0.13 | 0.16 | 1.32 |
|  | Weighted median | 4 | 0.18 | 0.08 | 0.02 | 1.2 |
|  | Inverse variance weighted | 4 | 0.18 | 0.07 | 0.01 | 1.2 |
|  | Simple mode | 4 | 0.08 | 0.14 | 0.61 | 1.08 |
|  | Weighted mode | 4 | 0.28 | 0.1 | 0.06 | 1.32 |
| SPART | MR Egger | 6 | 0.03 | 0.05 | 0.6 | 1.03 |
|  | Weighted median | 6 | 0.05 | 0.03 | 0.05 | 1.05 |
|  | Inverse variance weighted | 6 | 0.05 | 0.02 | 0.02 | 1.05 |
|  | Simple mode | 6 | 0.05 | 0.03 | 0.15 | 1.05 |
|  | Weighted mode | 6 | 0.05 | 0.03 | 0.14 | 1.05 |
| SPTBN1 | MR Egger | 4 | 0.03 | 0.07 | 0.67 | 1.03 |
|  | Weighted median | 4 | 0.1 | 0.04 | 0.01 | 1.1 |
|  | Inverse variance weighted | 4 | 0.1 | 0.03 | 0 | 1.11 |
|  | Simple mode | 4 | 0.19 | 0.07 | 0.07 | 1.21 |
|  | Weighted mode | 4 | 0.08 | 0.04 | 0.12 | 1.08 |
| STAG3 | MR Egger | 4 | -0.05 | 0.06 | 0.48 | 0.95 |
|  | Weighted median | 4 | -0.11 | 0.03 | 0 | 0.9 |
|  | Inverse variance weighted | 4 | -0.1 | 0.03 | 0 | 0.9 |
|  | Simple mode | 4 | -0.1 | 0.05 | 0.15 | 0.91 |
|  | Weighted mode | 4 | -0.11 | 0.04 | 0.06 | 0.9 |
| STMN3 | MR Egger | 5 | 0 | 0.1 | 0.98 | 1 |
|  | Weighted median | 5 | 0.1 | 0.04 | 0.01 | 1.11 |
|  | Inverse variance weighted | 5 | 0.12 | 0.05 | 0.02 | 1.12 |
|  | Simple mode | 5 | 0.12 | 0.06 | 0.13 | 1.13 |
|  | Weighted mode | 5 | 0.09 | 0.04 | 0.1 | 1.1 |
| STN1 | MR Egger | 3 | -0.22 | 0.3 | 0.6 | 0.8 |
|  | Weighted median | 3 | -0.2 | 0.07 | 0 | 0.82 |
|  | Inverse variance weighted | 3 | -0.19 | 0.06 | 0 | 0.83 |
|  | Simple mode | 3 | -0.23 | 0.09 | 0.12 | 0.79 |
|  | Weighted mode | 3 | -0.2 | 0.08 | 0.13 | 0.82 |
| SULF2 | MR Egger | 10 | -0.05 | 0.03 | 0.12 | 0.95 |
|  | Weighted median | 10 | -0.05 | 0.02 | 0.04 | 0.95 |
|  | Inverse variance weighted | 10 | -0.04 | 0.02 | 0.04 | 0.96 |
|  | Simple mode | 10 | -0.07 | 0.04 | 0.12 | 0.94 |
|  | Weighted mode | 10 | -0.05 | 0.02 | 0.05 | 0.95 |
| TAF7 | MR Egger | 4 | 0.13 | 0.08 | 0.27 | 1.14 |
|  | Weighted median | 4 | 0.13 | 0.05 | 0.01 | 1.14 |
|  | Inverse variance weighted | 4 | 0.11 | 0.04 | 0.01 | 1.12 |
|  | Simple mode | 4 | 0 | 0.08 | 0.96 | 1 |
|  | Weighted mode | 4 | 0.15 | 0.06 | 0.08 | 1.16 |
| TBC1D8 | MR Egger | 5 | 0.07 | 0.06 | 0.31 | 1.08 |
|  | Weighted median | 5 | 0.08 | 0.04 | 0.06 | 1.08 |
|  | Inverse variance weighted | 5 | 0.09 | 0.04 | 0.02 | 1.1 |
|  | Simple mode | 5 | 0.14 | 0.08 | 0.13 | 1.16 |
|  | Weighted mode | 5 | 0.08 | 0.04 | 0.17 | 1.08 |
| TCF4 | MR Egger | 9 | 0.38 | 0.18 | 0.08 | 1.46 |
|  | Weighted median | 9 | 0.26 | 0.09 | 0 | 1.3 |
|  | Inverse variance weighted | 9 | 0.16 | 0.07 | 0.03 | 1.17 |
|  | Simple mode | 9 | 0.27 | 0.15 | 0.11 | 1.31 |
|  | Weighted mode | 9 | 0.28 | 0.11 | 0.03 | 1.32 |
| TCP11L1 | MR Egger | 3 | 0.26 | 0.12 | 0.27 | 1.3 |
|  | Weighted median | 3 | 0.16 | 0.04 | 0 | 1.17 |
|  | Inverse variance weighted | 3 | 0.15 | 0.04 | 0 | 1.17 |
|  | Simple mode | 3 | 0.1 | 0.09 | 0.37 | 1.1 |
|  | Weighted mode | 3 | 0.16 | 0.05 | 0.08 | 1.18 |
| TDRD9 | MR Egger | 6 | -0.07 | 0.04 | 0.19 | 0.93 |
|  | Weighted median | 6 | -0.06 | 0.03 | 0.04 | 0.94 |
|  | Inverse variance weighted | 6 | -0.06 | 0.03 | 0.02 | 0.94 |
|  | Simple mode | 6 | -0.08 | 0.07 | 0.3 | 0.92 |
|  | Weighted mode | 6 | -0.06 | 0.03 | 0.09 | 0.94 |
| TEK | MR Egger | 4 | 0.08 | 0.04 | 0.18 | 1.09 |
|  | Weighted median | 4 | 0.07 | 0.03 | 0 | 1.07 |
|  | Inverse variance weighted | 4 | 0.05 | 0.02 | 0.03 | 1.05 |
|  | Simple mode | 4 | 0.07 | 0.05 | 0.26 | 1.07 |
|  | Weighted mode | 4 | 0.07 | 0.03 | 0.07 | 1.07 |
| TGS1 | MR Egger | 3 | -0.08 | 0.05 | 0.33 | 0.92 |
|  | Weighted median | 3 | -0.11 | 0.03 | 0 | 0.9 |
|  | Inverse variance weighted | 3 | -0.12 | 0.04 | 0 | 0.89 |
|  | Simple mode | 3 | -0.13 | 0.1 | 0.31 | 0.88 |
|  | Weighted mode | 3 | -0.11 | 0.03 | 0.08 | 0.9 |
| TMEM273 | MR Egger | 6 | -0.06 | 0.06 | 0.38 | 0.94 |
|  | Weighted median | 6 | -0.07 | 0.03 | 0.05 | 0.93 |
|  | Inverse variance weighted | 6 | -0.08 | 0.03 | 0.01 | 0.92 |
|  | Simple mode | 6 | -0.21 | 0.06 | 0.01 | 0.81 |
|  | Weighted mode | 6 | -0.07 | 0.03 | 0.11 | 0.94 |
| TMEM80 | MR Egger | 4 | 0.19 | 0.09 | 0.17 | 1.21 |
|  | Weighted median | 4 | 0.1 | 0.03 | 0 | 1.11 |
|  | Inverse variance weighted | 4 | 0.09 | 0.04 | 0.01 | 1.1 |
|  | Simple mode | 4 | 0.09 | 0.04 | 0.11 | 1.1 |
|  | Weighted mode | 4 | 0.1 | 0.03 | 0.05 | 1.11 |
| TNFRSF13B | MR Egger | 6 | 0.06 | 0.35 | 0.87 | 1.06 |
|  | Weighted median | 6 | 0.25 | 0.12 | 0.03 | 1.28 |
|  | Inverse variance weighted | 6 | 0.22 | 0.09 | 0.02 | 1.24 |
|  | Simple mode | 6 | 0.26 | 0.19 | 0.22 | 1.29 |
|  | Weighted mode | 6 | 0.25 | 0.18 | 0.22 | 1.28 |
| TOB1 | MR Egger | 3 | -0.34 | 0.26 | 0.41 | 0.71 |
|  | Weighted median | 3 | -0.08 | 0.04 | 0.05 | 0.93 |
|  | Inverse variance weighted | 3 | -0.08 | 0.04 | 0.05 | 0.93 |
|  | Simple mode | 3 | -0.02 | 0.06 | 0.74 | 0.98 |
|  | Weighted mode | 3 | -0.09 | 0.05 | 0.19 | 0.92 |
| TOGARAM2 | MR Egger | 3 | -0.14 | 0.1 | 0.39 | 0.87 |
|  | Weighted median | 3 | -0.1 | 0.05 | 0.04 | 0.9 |
|  | Inverse variance weighted | 3 | -0.1 | 0.05 | 0.05 | 0.91 |
|  | Simple mode | 3 | -0.1 | 0.08 | 0.35 | 0.91 |
|  | Weighted mode | 3 | -0.11 | 0.05 | 0.18 | 0.9 |
| TRANK1 | MR Egger | 4 | 0.01 | 0.08 | 0.92 | 1.01 |
|  | Weighted median | 4 | 0.06 | 0.04 | 0.12 | 1.07 |
|  | Inverse variance weighted | 4 | 0.08 | 0.04 | 0.05 | 1.09 |
|  | Simple mode | 4 | 0.07 | 0.1 | 0.51 | 1.08 |
|  | Weighted mode | 4 | 0.06 | 0.04 | 0.24 | 1.07 |
| TRAPPC14 | MR Egger | 3 | 0.19 | 0.09 | 0.28 | 1.2 |
|  | Weighted median | 3 | 0.12 | 0.05 | 0.01 | 1.13 |
|  | Inverse variance weighted | 3 | 0.12 | 0.04 | 0.01 | 1.13 |
|  | Simple mode | 3 | 0.05 | 0.09 | 0.62 | 1.05 |
|  | Weighted mode | 3 | 0.13 | 0.05 | 0.1 | 1.14 |
| TRBV4-2 | MR Egger | 3 | 0.15 | 0.09 | 0.34 | 1.17 |
|  | Weighted median | 3 | 0.09 | 0.05 | 0.04 | 1.1 |
|  | Inverse variance weighted | 3 | 0.09 | 0.04 | 0.05 | 1.09 |
|  | Simple mode | 3 | 0.13 | 0.09 | 0.28 | 1.14 |
|  | Weighted mode | 3 | 0.09 | 0.05 | 0.19 | 1.1 |
| TRPC6 | MR Egger | 3 | -0.03 | 0.12 | 0.84 | 0.97 |
|  | Weighted median | 3 | -0.1 | 0.05 | 0.06 | 0.91 |
|  | Inverse variance weighted | 3 | -0.1 | 0.05 | 0.03 | 0.9 |
|  | Simple mode | 3 | -0.13 | 0.1 | 0.33 | 0.88 |
|  | Weighted mode | 3 | -0.09 | 0.05 | 0.2 | 0.91 |
| TXK | MR Egger | 5 | -0.05 | 0.06 | 0.48 | 0.95 |
|  | Weighted median | 5 | -0.08 | 0.04 | 0.06 | 0.93 |
|  | Inverse variance weighted | 5 | -0.1 | 0.05 | 0.05 | 0.91 |
|  | Simple mode | 5 | -0.06 | 0.12 | 0.65 | 0.94 |
|  | Weighted mode | 5 | -0.07 | 0.04 | 0.13 | 0.93 |
| TYK2 | MR Egger | 4 | 0.13 | 0.08 | 0.24 | 1.14 |
|  | Weighted median | 4 | 0.14 | 0.05 | 0 | 1.15 |
|  | Inverse variance weighted | 4 | 0.14 | 0.05 | 0 | 1.15 |
|  | Simple mode | 4 | 0.07 | 0.11 | 0.54 | 1.08 |
|  | Weighted mode | 4 | 0.13 | 0.05 | 0.09 | 1.14 |
| UBE2L6 | MR Egger | 5 | -0.06 | 0.14 | 0.7 | 0.94 |
|  | Weighted median | 5 | -0.16 | 0.07 | 0.02 | 0.85 |
|  | Inverse variance weighted | 5 | -0.16 | 0.06 | 0 | 0.85 |
|  | Simple mode | 5 | -0.27 | 0.11 | 0.07 | 0.76 |
|  | Weighted mode | 5 | -0.1 | 0.08 | 0.28 | 0.91 |
| UCKL1 | MR Egger | 5 | -0.24 | 0.07 | 0.04 | 0.78 |
|  | Weighted median | 5 | -0.2 | 0.04 | 0 | 0.82 |
|  | Inverse variance weighted | 5 | -0.18 | 0.04 | 0 | 0.83 |
|  | Simple mode | 5 | -0.14 | 0.07 | 0.11 | 0.87 |
|  | Weighted mode | 5 | -0.2 | 0.05 | 0.01 | 0.82 |
| USP10 | MR Egger | 4 | 0.04 | 0.13 | 0.77 | 1.05 |
|  | Weighted median | 4 | 0.19 | 0.1 | 0.04 | 1.21 |
|  | Inverse variance weighted | 4 | 0.21 | 0.08 | 0.01 | 1.23 |
|  | Simple mode | 4 | 0.34 | 0.15 | 0.11 | 1.4 |
|  | Weighted mode | 4 | 0.13 | 0.1 | 0.3 | 1.14 |
| USP39 | MR Egger | 6 | -0.07 | 0.1 | 0.55 | 0.94 |
|  | Weighted median | 6 | -0.1 | 0.04 | 0.03 | 0.91 |
|  | Inverse variance weighted | 6 | -0.11 | 0.05 | 0.04 | 0.9 |
|  | Simple mode | 6 | -0.13 | 0.12 | 0.32 | 0.88 |
|  | Weighted mode | 6 | -0.1 | 0.04 | 0.09 | 0.91 |
| VARS2 | MR Egger | 5 | -0.08 | 0.17 | 0.66 | 0.92 |
|  | Weighted median | 5 | -0.1 | 0.03 | 0 | 0.9 |
|  | Inverse variance weighted | 5 | -0.1 | 0.03 | 0 | 0.91 |
|  | Simple mode | 5 | -0.11 | 0.05 | 0.1 | 0.89 |
|  | Weighted mode | 5 | -0.1 | 0.04 | 0.05 | 0.9 |
| VKORC1L1 | MR Egger | 3 | -0.09 | 0.08 | 0.47 | 0.92 |
|  | Weighted median | 3 | -0.08 | 0.04 | 0.04 | 0.93 |
|  | Inverse variance weighted | 3 | -0.07 | 0.04 | 0.04 | 0.93 |
|  | Simple mode | 3 | -0.07 | 0.05 | 0.25 | 0.93 |
|  | Weighted mode | 3 | -0.08 | 0.04 | 0.17 | 0.93 |
| VMP1 | MR Egger | 6 | 0.13 | 0.14 | 0.41 | 1.14 |
|  | Weighted median | 6 | 0.08 | 0.09 | 0.33 | 1.09 |
|  | Inverse variance weighted | 6 | 0.16 | 0.07 | 0.02 | 1.18 |
|  | Simple mode | 6 | 0.07 | 0.12 | 0.59 | 1.07 |
|  | Weighted mode | 6 | 0.08 | 0.1 | 0.5 | 1.08 |
| WDFY3 | MR Egger | 3 | -0.35 | 0.31 | 0.46 | 0.7 |
|  | Weighted median | 3 | -0.17 | 0.1 | 0.09 | 0.85 |
|  | Inverse variance weighted | 3 | -0.2 | 0.09 | 0.03 | 0.82 |
|  | Simple mode | 3 | -0.06 | 0.15 | 0.73 | 0.94 |
|  | Weighted mode | 3 | -0.28 | 0.11 | 0.12 | 0.75 |
| WDR41 | MR Egger | 10 | 0.09 | 0.07 | 0.21 | 1.1 |
|  | Weighted median | 10 | 0.08 | 0.03 | 0 | 1.09 |
|  | Inverse variance weighted | 10 | 0.07 | 0.02 | 0 | 1.07 |
|  | Simple mode | 10 | 0.07 | 0.04 | 0.1 | 1.08 |
|  | Weighted mode | 10 | 0.08 | 0.03 | 0.02 | 1.08 |
| WFS1 | MR Egger | 3 | -0.08 | 0.15 | 0.69 | 0.93 |
|  | Weighted median | 3 | -0.11 | 0.06 | 0.05 | 0.89 |
|  | Inverse variance weighted | 3 | -0.11 | 0.06 | 0.05 | 0.9 |
|  | Simple mode | 3 | -0.12 | 0.08 | 0.28 | 0.89 |
|  | Weighted mode | 3 | -0.11 | 0.06 | 0.2 | 0.9 |
| WSB1 | MR Egger | 3 | 0.08 | 0.17 | 0.73 | 1.08 |
|  | Weighted median | 3 | 0.09 | 0.04 | 0.02 | 1.1 |
|  | Inverse variance weighted | 3 | 0.09 | 0.04 | 0.02 | 1.1 |
|  | Simple mode | 3 | 0.11 | 0.05 | 0.15 | 1.12 |
|  | Weighted mode | 3 | 0.08 | 0.04 | 0.21 | 1.08 |
| XRCC3 | MR Egger | 3 | 0.07 | 0.09 | 0.6 | 1.07 |
|  | Weighted median | 3 | 0.09 | 0.04 | 0.05 | 1.09 |
|  | Inverse variance weighted | 3 | 0.09 | 0.04 | 0.04 | 1.09 |
|  | Simple mode | 3 | 0.11 | 0.07 | 0.25 | 1.11 |
|  | Weighted mode | 3 | 0.09 | 0.05 | 0.19 | 1.09 |
| ZBTB25 | MR Egger | 4 | -0.1 | 0.09 | 0.35 | 0.9 |
|  | Weighted median | 4 | -0.07 | 0.03 | 0.04 | 0.93 |
|  | Inverse variance weighted | 4 | -0.07 | 0.03 | 0.04 | 0.94 |
|  | Simple mode | 4 | -0.01 | 0.08 | 0.9 | 0.99 |
|  | Weighted mode | 4 | -0.07 | 0.04 | 0.13 | 0.93 |
| ZFP30 | MR Egger | 3 | 0.05 | 0.1 | 0.71 | 1.05 |
|  | Weighted median | 3 | 0.11 | 0.04 | 0.01 | 1.11 |
|  | Inverse variance weighted | 3 | 0.1 | 0.05 | 0.02 | 1.11 |
|  | Simple mode | 3 | 0.14 | 0.07 | 0.18 | 1.15 |
|  | Weighted mode | 3 | 0.1 | 0.05 | 0.15 | 1.11 |
| ZFP82 | MR Egger | 4 | 0.08 | 0.07 | 0.37 | 1.08 |
|  | Weighted median | 4 | 0.08 | 0.04 | 0.06 | 1.08 |
|  | Inverse variance weighted | 4 | 0.09 | 0.04 | 0.02 | 1.09 |
|  | Simple mode | 4 | 0.03 | 0.06 | 0.61 | 1.03 |
|  | Weighted mode | 4 | 0.08 | 0.04 | 0.15 | 1.08 |
| ZMYND15 | MR Egger | 5 | 0.09 | 0.07 | 0.31 | 1.09 |
|  | Weighted median | 5 | 0.08 | 0.04 | 0.05 | 1.09 |
|  | Inverse variance weighted | 5 | 0.08 | 0.04 | 0.04 | 1.09 |
|  | Simple mode | 5 | 0.12 | 0.09 | 0.27 | 1.12 |
|  | Weighted mode | 5 | 0.08 | 0.04 | 0.13 | 1.09 |
| ZNF107 | MR Egger | 4 | 0.2 | 0.12 | 0.24 | 1.22 |
|  | Weighted median | 4 | 0.14 | 0.07 | 0.05 | 1.15 |
|  | Inverse variance weighted | 4 | 0.14 | 0.06 | 0.02 | 1.15 |
|  | Simple mode | 4 | 0.14 | 0.09 | 0.22 | 1.15 |
|  | Weighted mode | 4 | 0.14 | 0.08 | 0.18 | 1.15 |
| ZNF135 | MR Egger | 3 | 0.24 | 0.33 | 0.59 | 1.28 |
|  | Weighted median | 3 | 0.13 | 0.05 | 0.01 | 1.14 |
|  | Inverse variance weighted | 3 | 0.12 | 0.05 | 0.02 | 1.13 |
|  | Simple mode | 3 | 0.15 | 0.06 | 0.14 | 1.16 |
|  | Weighted mode | 3 | 0.14 | 0.06 | 0.14 | 1.15 |
| ZNF204P | MR Egger | 3 | 0.14 | 0.17 | 0.55 | 1.15 |
|  | Weighted median | 3 | 0.18 | 0.06 | 0 | 1.2 |
|  | Inverse variance weighted | 3 | 0.18 | 0.06 | 0.01 | 1.19 |
|  | Simple mode | 3 | 0.24 | 0.09 | 0.13 | 1.27 |
|  | Weighted mode | 3 | 0.19 | 0.07 | 0.1 | 1.21 |
| ZNF273 | MR Egger | 3 | -0.37 | 0.22 | 0.35 | 0.69 |
|  | Weighted median | 3 | -0.25 | 0.12 | 0.05 | 0.78 |
|  | Inverse variance weighted | 3 | -0.25 | 0.11 | 0.02 | 0.78 |
|  | Simple mode | 3 | -0.19 | 0.15 | 0.32 | 0.83 |
|  | Weighted mode | 3 | -0.27 | 0.16 | 0.22 | 0.76 |
| ZNF333 | MR Egger | 3 | 0.04 | 0.08 | 0.73 | 1.04 |
|  | Weighted median | 3 | 0.09 | 0.04 | 0.02 | 1.09 |
|  | Inverse variance weighted | 3 | 0.09 | 0.04 | 0.02 | 1.09 |
|  | Simple mode | 3 | 0.15 | 0.06 | 0.13 | 1.17 |
|  | Weighted mode | 3 | 0.06 | 0.04 | 0.29 | 1.06 |
| ZNF665 | MR Egger | 4 | 0.13 | 0.3 | 0.71 | 1.14 |
|  | Weighted median | 4 | 0.22 | 0.09 | 0.02 | 1.24 |
|  | Inverse variance weighted | 4 | 0.24 | 0.08 | 0 | 1.27 |
|  | Simple mode | 4 | 0.19 | 0.13 | 0.22 | 1.21 |
|  | Weighted mode | 4 | 0.2 | 0.12 | 0.18 | 1.22 |
| ZNF672 | MR Egger | 5 | -0.11 | 0.19 | 0.61 | 0.9 |
|  | Weighted median | 5 | -0.13 | 0.07 | 0.05 | 0.88 |
|  | Inverse variance weighted | 5 | -0.16 | 0.08 | 0.04 | 0.85 |
|  | Simple mode | 5 | -0.11 | 0.1 | 0.37 | 0.9 |
|  | Weighted mode | 5 | -0.13 | 0.07 | 0.15 | 0.88 |

**Abbreviations**: B: Beta-value; LUAD: Lung adenocarcinoma; MR: Mendelian randomization; Nsnp: Number of SNPs; OR: Odds ratio; Snp: Single nucleotide polymorphism; Se: Standard error.
