# Supplementary material for: Integrative multi-omics reveals energy metabolism–related prognostic signatures and immunogenetic landscapes in lung adenocarcinoma
Source: Front Immunol. 2025 Oct 14;16:1679464. doi: 10.3389/fimmu.2025.1679464 (PMC12558868; doi:10.3389/fimmu.2025.1679464)
Supplement: Supplementary Table 3 — The 9 overlapping genes identified from MR and DEG intersection. [file Table3.docx]

**Table S3** The 9 intersecting genes were based on MR analysis and differential analysis.

| **Gene** | **logFC** | **AveExpr** | **t** | **P-Value** | **adj.P.Val** | **B** |
| --- | --- | --- | --- | --- | --- | --- |
| VMP1 | 1.305 | 5.233 | 12.308 | 0.000 | 0.000 | 59.603 |
| WFS1 | -1.619 | 3.707 | -16.357 | 0.000 | 0.000 | 102.393 |
| POU2AF1 | 1.000 | 1.851 | 6.708 | 0.000 | 0.000 | 13.928 |
| SPTBN1 | -1.633 | 4.565 | -17.167 | 0.000 | 0.000 | 111.565 |
| TEK | -2.735 | 1.898 | -27.413 | 0.000 | 0.000 | 233.782 |
| NCKAP1L | -1.136 | 2.522 | -10.159 | 0.000 | 0.000 | 39.772 |
| LOXL2 | 1.148 | 2.799 | 7.650 | 0.000 | 0.000 | 20.136 |
| RCC1 | 1.578 | 3.993 | 18.309 | 0.000 | 0.000 | 124.722 |
| NOTCH4 | -1.357 | 1.818 | -16.643 | 0.000 | 0.000 | 105.616 |

**Abbreviations**: AveExpr: Average expression; B: Bayes moderated t-statistic; log FC: Log Fold Change; MR: Mendelian randomization.
