# Supplementary material for: Integrative multi-omics reveals energy metabolism–related prognostic signatures and immunogenetic landscapes in lung adenocarcinoma
Source: Front Immunol. 2025 Oct 14;16:1679464. doi: 10.3389/fimmu.2025.1679464 (PMC12558868; doi:10.3389/fimmu.2025.1679464)
Supplement: Supplementary Table 4 — Cochran’s Q and Egger intercept tests of heterogeneity and pleiotropy in MR analysis. [file Table4.docx]

**Table S4** The results of heterogeneity and horizontal pleiotropy tests in intersecting genes.

| **Exposure** | **Heterogeneity test** | | | | | | |  | **Pleiotropy test** | | |
| --- | --- | --- | --- | --- | --- | --- | --- | --- | --- | --- | --- |
|  | **MR Egger** | | |  | **IVW** | | |  | **MR Egger** | | |
|  | **Q** | **Q_df** | **Q_pval** |  | **Q** | **Q_df** | **Q_pval** |  | **egger_intercept** | **se** | **P-val** |
| WFS1 | 0.104 | 1 | 0.746 |  | 0.160 | 2 | 0.923 |  | -0.011 | 0.046 | 0.853 |
| TEK | 1.956 | 2 | 0.376 |  | 2.836 | 3 | 0.418 |  | -0.015 | 0.016 | 0.447 |
| SPTBN1 | 0.107 | 2 | 0.948 |  | 1.501 | 3 | 0.682 |  | 0.021 | 0.018 | 0.359 |
| RUNX2 | 4.975 | 3 | 0.174 |  | 5.211 | 4 | 0.266 |  | 0.008 | 0.022 | 0.731 |
| RCC1 | 1.808 | 1 | 0.179 |  | 1.814 | 2 | 0.404 |  | 0.004 | 0.070 | 0.964 |
| NOTCH4 | 1.301 | 1 | 0.254 |  | 1.525 | 2 | 0.467 |  | 0.010 | 0.023 | 0.750 |
| NCKAP1L | 1.662 | 1 | 0.197 |  | 2.176 | 2 | 0.337 |  | -0.014 | 0.025 | 0.677 |
| LOXL2 | 0.083 | 1 | 0.774 |  | 0.351 | 2 | 0.839 |  | -0.025 | 0.048 | 0.696 |
| VMP1 | 3.600 | 4 | 0.463 |  | 3.677 | 5 | 0.597 |  | 0.006 | 0.020 | 0.795 |

**Abbreviations**: df: Degree of freedom; IVW: Inverse variance weighted; MR: Mendelian randomization; Q: Cochran’s Q statistic; se: Standard error.
