# Supplementary material for: Integrative multi-omics reveals energy metabolism–related prognostic signatures and immunogenetic landscapes in lung adenocarcinoma
Source: Front Immunol. 2025 Oct 14;16:1679464. doi: 10.3389/fimmu.2025.1679464 (PMC12558868; doi:10.3389/fimmu.2025.1679464)
Supplement: Supplementary Table 5 — KEGG enrichment analysis results of DEGs between HRG&LRG. [file Table5.docx]

**Table S5** The top 6 items that were most significant in BP, CC, and MF in the GO enrichment analysis.

| **ONTOLOGY** | | **Description** | **p-value** | **p.adjust** | **q-value** | **Count** |
| --- | --- | --- | --- | --- | --- | --- |
|  | wound healing | 5.69E-23 | 3.13E-19 | 1.99E-19 | 76 |  |
|  | regulation of body fluid levels | 2.68E-20 | 7.39E-17 | 4.69E-17 | 66 |  |
| BP | positive regulation of cell adhesion | 1.45E-18 | 2.67E-15 | 1.69E-15 | 74 |  |
|  | external encapsulating structure organization | 4.71E-18 | 6.49E-15 | 4.12E-15 | 59 |  |
|  | extracellular matrix organization | 1.56E-17 | 1.65E-14 | 1.05E-14 | 58 |  |
|  | extracellular structure organization | 1.80E-17 | 1.65E-14 | 1.05E-14 | 58 |  |
|  | collagen-containing extracellular matrix | 4.77E-17 | 2.59E-14 | 1.82E-14 | 65 |  |
|  | membrane raft | 9.83E-13 | 1.96E-10 | 1.37E-10 | 45 |  |
| CC | membrane microdomain | 1.11E-12 | 1.96E-10 | 1.37E-10 | 45 |  |
|  | external side of plasma membrane | 1.44E-12 | 1.96E-10 | 1.37E-10 | 55 |  |
|  | basal part of cell | 9.57E-12 | 1.04E-09 | 7.30E-10 | 45 |  |
|  | endocytic vesicle | 2.77E-11 | 2.51E-09 | 1.76E-09 | 48 |  |
|  | glycosaminoglycan binding | 6.36E-13 | 6.32E-10 | 5.12E-10 | 42 |  |
|  | amide binding | 4.47E-11 | 2.22E-08 | 1.80E-08 | 54 |  |
| MF | peptide binding | 1.16E-10 | 3.83E-08 | 3.10E-08 | 47 |  |
|  | integrin binding | 1.54E-10 | 3.83E-08 | 3.10E-08 | 30 |  |
|  | extracellular matrix structural constituent | 6.36E-10 | 1.26E-07 | 1.02E-07 | 30 |  |
|  | heparin binding | 3.34E-08 | 5.13E-06 | 4.16E-06 | 28 |  |

**Abbreviations**: BP: Biological Process; CC: Cellular Component; GO: Gene Ontology; MF: Molecular Function.
