# Supplementary material for: Integrative multi-omics reveals energy metabolism–related prognostic signatures and immunogenetic landscapes in lung adenocarcinoma
Source: Front Immunol. 2025 Oct 14;16:1679464. doi: 10.3389/fimmu.2025.1679464 (PMC12558868; doi:10.3389/fimmu.2025.1679464)
Supplement: Supplementary Table 6 — GO enrichment analysis results of DEGs between HRG&LRG. [file Table6.docx]

**Table S6** The top 20 most significant pathways in KEGG pathway enrichment analysis.

| **Description** | **ES** | **p-value** | **p.adjust** | **q-value** | **NES** |
| --- | --- | --- | --- | --- | --- |
| DNA REPLICATION LICENSING | 0.8456 | 2.41E-09 | 1.62E-07 | 1.30E-07 | 2.5809 |
| PRE IC FORMATION | 0.8383 | 1.57E-09 | 1.62E-07 | 1.30E-07 | 2.6374 |
| ORGANIZATION OF THE OUTER KINETOCHOR | 0.8228 | 1.11E-06 | 5.26E-05 | 4.19E-05 | 2.3285 |
| ORGANIZATION OF THE INNER KINETOCHORE | 0.8205 | 6.24E-06 | 0.0002 | 0.0002 | 2.2094 |
| TRAIP DEPENDENT REPLISOME DISASSEMBLY | 0.8190 | 6.44E-06 | 0.0002 | 0.0002 | 2.2055 |
| ORIGIN UNWINDING AND ELONGATION | 0.8122 | 2.57E-09 | 1.62E-07 | 1.30E-07 | 2.6625 |
| MICROTUBULE DEPOLYMERIZATION | 0.7979 | 4.29E-05 | 0.0007 | 0.0006 | 2.1432 |
| COHESIN DISSOCIATION IN PROPHASE | 0.7895 | 2.99E-05 | 0.0006 | 0.0005 | 2.1260 |
| DNA REPLICATION TERMINATION | 0.7808 | 9.94E-06 | 0.0002 | 0.0002 | 2.2849 |
| CONDENSIN LOADING | 0.7767 | 8.18E-06 | 0.0002 | 0.0002 | 2.2419 |
| KINETOCHORE FIBER ORGANIZATION | 0.7746 | 0.0001 | 0.0016 | 0.0013 | 2.0808 |
| AMPLIFIED MYC TO P27 CELL CYCLE G1 S | 0.7612 | 0.0002 | 0.0022 | 0.0018 | 2.0447 |
| LONG PATCH BER | 0.7434 | 0.0002 | 0.0022 | 0.0018 | 2.0450 |
| HOMOLOGOUS RECOMBINATION IN ICLR | 0.7432 | 0.0005 | 0.0036 | 0.0029 | 1.9963 |
| SPINDLE ASSEMBLY CHECKPOINT SIGNALING | 0.7363 | 0.0001 | 0.0016 | 0.0013 | 2.0836 |
| DISASSEMBLY OF MCC | 0.7172 | 0.0006 | 0.0042 | 0.0034 | 1.9728 |
| FANCONI ANEMIA PATHWAY | 0.7092 | 0.0006 | 0.0041 | 0.0033 | 1.9765 |
| GLYCOLYSIS | 0.6915 | 0.0009 | 0.0057 | 0.0045 | 1.8621 |
| HTLV 1 TAX TO SPINDLE ASSEMBLY CHECKPOINT SIGNALING | 0.6865 | 0.0023 | 0.0133 | 0.0106 | 1.8441 |
| COHESIN DISSOCIATION IN ANAPHASE | 0.6725 | 0.0014 | 0.0083 | 0.0066 | 1.8110 |

**Abbreviations**: KEGG: Kyoto Encyclopedia of Genes and Genomes; ES: enrichment Score.
