# Supplementary material for: Integrative multi-omics reveals energy metabolism–related prognostic signatures and immunogenetic landscapes in lung adenocarcinoma
Source: Front Immunol. 2025 Oct 14;16:1679464. doi: 10.3389/fimmu.2025.1679464 (PMC12558868; doi:10.3389/fimmu.2025.1679464)
Supplement: Supplementary Table 7 — Estimated IC50 values of 89 antitumor drugs for HRG&LRG. [file Table7.docx]

**Table S7** The 83 chemotherapeutic drugs obtained from drug sensitivity analysis.

| **Drugs** | **Low-risk group** |  | **High-risk group** |  | **P-value** |
| --- | --- | --- | --- | --- | --- |
|  | **IC50 (25%-75%)** |  | **IC50 (25%-75%)** |  |  |
| ABT737 | 7.28(5.32-11.48) |  | 10.02(6.76-14.11) |  | 0.00 |
| Afuresertib | 10.84(7.68-15.79) |  | 14.15(9.37-20.15) |  | 0.00 |
| AGI.5198 | 95.70(83.26-117.55) |  | 110.98(93.16-129.03) |  | 0.00 |
| AGI.6780 | 57.92(46.76-70.42) |  | 67.13(53.18-86.26) |  | 0.00 |
| AMG.319 | 113.47(84.16-159.02) |  | 140.21(107.93-189.23) |  | 0.00 |
| Axitinib | 20.35(16.50-23.78) |  | 24.25(20.04-28.28) |  | 0.00 |
| AZD1208 | 175.30(139.02-238.52) |  | 219.63(166.69-282.73) |  | 0.00 |
| AZD5991 | 67.39(34.31-109.28) |  | 96.01(44.13-163.77) |  | 0.00 |
| AZD6482 | 22.55(18.44-26.54) |  | 26.29(22.34-31.03) |  | 0.00 |
| BIBR.1532 | 130.07(101.21-170.85) |  | 158.00(121.40-198.46) |  | 0.00 |
| BI.2536 | 1.61(1.13-2.10) |  | 1.27(0.87-1.72) |  | 0.00 |
| BMS.536924 | 8.63(6.20-11.93) |  | 7.27(5.53-10.32TIN) |  | 0.00 |
| BMS.754807 | 1.09(0.51-1.81) |  | 1.89(1.14-2.74) |  | 0.00 |
| Carmustine | 414.43(345.97-535.14) |  | 480.97(355.57-617.85) |  | 0.00 |
| CDK9_5038 | 0.08(0.06-0.12) |  | 0.10(0.07-0.16) |  | 0.00 |
| CDK9_5576 | 0.60(0.44-0.82) |  | 0.73(0.52-1.04) |  | 0.00 |
| Crizotinib | 22.07(16.57-31.64) |  | 26.46(19.28-35.90) |  | 0.00 |
| Cyclophosphamide | 155.71(130.64-197.73) |  | 188.68(148.45-234.68) |  | 0.00 |
| CZC24832 | 144.58(113.61-181.71) |  | 169.75(134.69-213.47) |  | 0.00 |
| Dasatinib | 5.89(2.98-10.88) |  | 5.24(2.10-9.03) |  | 0.02 |
| Dinaciclib | 0.06(0.04-0.07) |  | 0.06(0.05-0.09) |  | 0.00 |
| Docetaxel_1007 | 0.01(0.01-0.02) |  | 0.01(0.01-0.02) |  | 0.01 |
| Doramapimod | 80.60(69.92-96.03) |  | 98.96(86.27-116.23) |  | 0.00 |
| Elephantin | 28.46(20.45-41.53) |  | 35.80(24.80-49.42) |  | 0.00 |
| EPZ004777 | 150.87(116.19-208.35) |  | 189.80(142.62-238.32) |  | 0.00 |
| EPZ5676 | 232.33(175.50-305.19) |  | 279.73(212.76-365.79) |  | 0.00 |
| ERK_6604 | 32.55(23.51-48.26) |  | 27.98(20.29-41.66) |  | 0.00 |
| Fulvestrant | 16.78(13.69-21.79) |  | 20.14(15.65-25.30) |  | 0.00 |
| Gallibiscoquinazole | 12.78(10.74-15.67) |  | 14.50 (11.63-17.68) |  | 0.00 |
| GNE.317 | 1.57(1.16-2.30) |  | 1.72(1.25-2.49) |  | 0.01 |
| GSK1904529A | 71.01(53.94-94.32) |  | 82.19(61.82-109.67) |  | 0.00 |
| GSK2578215A | 129.00(107.62-155.97) |  | 146.13(115.13-170.28) |  | 0.00 |
| GSK2606414 | 38.28(29.45-49.45) |  | 43.60(33.90-57.83) |  | 0.00 |
| GSK269962A | 15.87(12.77-19.14) |  | 18.80(15.22-23.03) |  | 0.00 |
| GSK343 | 15.49(13.02-18.19) |  | 17.64(14.85-21.10) |  | 0.00 |
| GSK591 | 86.16(70.57-112.64) |  | 107.92(83.56-134.20) |  | 0.00 |
| I.BRD9 | 71.36(52.49-94.89) |  | 87.19(65.41-113.79) |  | 0.00 |
| Ibrutinib | 83.31(54.99-122.00) |  | 96.50(64.08-144.62) |  | 0.00 |
| Ipatasertib | 29.87(21.00-44.15) |  | 34.67(25.29-50.76) |  | 0.00 |
| IRAK4_4710 | 131.78(109.42-158.90) |  | 148.50(118.82-181.06) |  | 0.00 |
| JAK1_8709 | 56.51(42.37-79.00) |  | 68.31(51.03-97.46) |  | 0.00 |
| JQ1 | 7.96(4.73-12.88) |  | 12.48(8.07-19.12) |  | 0.00 |
| KRAS.G12C.Inhibitor.12 | 68.21(49.14-96.21) |  | 94.41(61.46-132.39) |  | 0.00 |
| Lapatinib | 21.66(14.97-30.55) |  | 19.23(13.06-27.99) |  | 0.02 |
| LGK974 | 50.01(38.19-67.12) |  | 61.07(43.52-86.70) |  | 0.00 |
| LJI308 | 147.92(115.05-186.23) |  | 174.25(132.70-228.29) |  | 0.00 |
| LY2109761 | 155.88(117.63-209.49) |  | 193.25(148.26-254.07) |  | 0.00 |
| MIRA.1 | 199.27(149.23-285.96) |  | 245.42(186.55-331.93) |  | 0.00 |
| Mitoxantrone | 1.46(0.75-2.65) |  | 2.10(1.18-3.82) |  | 0.00 |
| ML323 | 82.92(68.16-102.25) |  | 94.40(68.79-124.49) |  | 0.00 |
| MN.64 | 105.65(83.20-133.66) |  | 116.57(93.97-153.66) |  | 0.00 |
| Navitoclax | 5.54(3.45-10.40) |  | 7.33(4.66-14.06) |  | 0.00 |
| Nelarabine | 376.19(290.44-495.72) |  | 452.34(341.64-567.06) |  | 0.00 |
| Nilotinib | 31.49(20.43-47.20) |  | 44.10(29.45-59.12) |  | 0.00 |
| Niraparib | 63.52(44.97-89.48) |  | 89.16(61.51-119.82) |  | 0.00 |
| NU7441 | 12.97(11.22-14.99) |  | 14.07(12.02-16.36) |  | 0.00 |
| OF.1 | 53.51(41.40-69.07) |  | 67.58(50.05-95.07) |  | 0.00 |
| Oxaliplatin | 126.21(85.02-191.79) |  | 179.76(109.67-260.55) |  | 0.00 |
| P22077 | 80.10(58.15-110.83) |  | 103.66(70.00-141.51) |  | 0.00 |
| PAK_5339 | 10.27(8.65-12.40) |  | 11.72(9.34-13.84) |  | 0.00 |
| Palbociclib | 34.18(23.70-54.08) |  | 42.81(28.43-66.89) |  | 0.00 |
| PCI.34051 | 79.54(57.98-106.99) |  | 105.79(76.54-140.51) |  | 0.00 |
| PFI3 | 178.18(145.35-216.32) |  | 201.67(170.02-242.41) |  | 0.00 |
| Picolinici.acid | 156.01(129.90-188.89) |  | 180.13(148.31-225.85) |  | 0.00 |
| PLX.4720 | 78.21(55.62-113.07) |  | 90.20(63.62-122.49) |  | 0.00 |
| PRT062607 | 22.71(16.96-31.38) |  | 27.02(21.29-35.49) |  | 0.00 |
| Pyridostatin | 27.19(19.20-36.87) |  | 30.36(23.89-39.94) |  | 0.00 |
| Ribociclib | 39.30(32.56-46.16)) |  | 46.84(39.98-56.55) |  | 0.00 |
| Ruxolitinib | 115.49(93.32-147.21) |  | 137.82(105.88-170.93) |  | 0.00 |
| Sabutoclax | 0.61(0.46-0.81) |  | 0.73(0.51-1.03) |  | 0.00 |
| SB216763 | 142.99(107.45-207.70) |  | 202.45(150.21-281.29) |  | 0.00 |
| SCH772984 | 14.28(9.57-23.87) |  | 13.45(6.89-22.93) |  | 0.05 |
| Sepantronium.bromide | 0.01(0.01-0.02) |  | 0.01(0.01-0.02) |  | 0.01 |
| Sinularin | 32.42(25.49-41.41) |  | 38.12(30.88-51.48) |  | 0.00 |
| Sorafenib | 12.40(9.35-17.08) |  | 16.35(11.52-21.10) |  | 0.00 |
| TAF1_5496 | 42.59(27.95-57.55) |  | 52.35(35.26-91.50) |  | 0.00 |
| Talazoparib | 21.78(13.86-32.68) |  | 26.82(16.31-46.26) |  | 0.00 |
| Tamoxifen | 33.97(23.34-43.39) |  | 37.55(29.80-47.19) |  | 0.01 |
| Temozolomide | 374.59(270.44-525.23) |  | 432.51(310.78-565.11) |  | 0.01 |
| Teniposide | 1.36(0.78-2.45) |  | 1.90(1.02-2.24) |  | 0.00 |
| Topotecan | 0.89(0.53-1.47) |  | 1.27(0.76-2.24) |  | 0.00 |
| Tozasertib | 17.54(13.65-23.00) |  | 20.36(15.39-25.09) |  | 0.00 |
| Uprosertib_1553 | 16.13(11.83-24.10) |  | 22.06(15.53-32.82) |  | 0.00 |
| Uprosertib_2106 | 13.57(9.35-21.19) |  | 18.29(11.29-28.96) |  | 0.00 |
| Venetoclax | 8.14(5.89-10.95) |  | 9.78(7.54-12.69) |  | 0.00 |
| Vorinostat | 4.00(3.04-5.07) |  | 4.55(3.50-5.93) |  | 0.00 |
| Wnt.C59 | 61.33(48.62-75.42) |  | 74.95(57.68-96.24) |  | 0.00 |
| Zoledronate | 38.43(29.99-50.25) |  | 48.76(37.41-59.18) |  | 0.00 |
| ZM447439 | 17.54(14.39-20.73) |  | 19.48(16.69-23.59) |  | 0.00 |

**Abbreviation:** IC50: half maximal inhibitory concentration.
