# Supplementary material for: Integrative multi-omics reveals energy metabolism–related prognostic signatures and immunogenetic landscapes in lung adenocarcinoma
Source: Front Immunol. 2025 Oct 14;16:1679464. doi: 10.3389/fimmu.2025.1679464 (PMC12558868; doi:10.3389/fimmu.2025.1679464)
Supplement: Supplementary Table 8 — Predicted lncRNA-miRNA-mRNA interactions in the EM-gene-associated ceRNA network. [file Table8.docx]

**Table S8** Potential ceRNA regulatory relationships between mRNA, lncRNA, and miRNA.

| **mRNA/lncRNA** | **miRNA** | **Interaction (-miRNA)** | **mRNA/lncRNA** | **miRNA** | **Interaction (-miRNA)** |
| --- | --- | --- | --- | --- | --- |
| LOXL2 | hsa-miR-767-5p | mRNA | FAR1-IT1 | hsa-miR-205-5p | lncRNA |
| LOXL2 | hsa-miR-29a-3p | mRNA | CTA-392E5.1 | hsa-miR-335-3p | lncRNA |
| RUNX2 | hsa-miR-767-3p | mRNA | CITF22-24E5.1 | hsa-miR-558 | lncRNA |
| RUNX2 | hsa-miR-1229-3p | mRNA | RP11-503N18.1 | hsa-miR-558 | lncRNA |
| NCKAP1L | hsa-miR-335-3p | mRNA | CTD-3193O13.11 | hsa-miR-1184 | lncRNA |
| LOXL2 | hsa-miR-765 | mRNA | TTLL10-AS1 | hsa-miR-30b-3p | lncRNA |
| NCKAP1L | hsa-miR-214-5p | mRNA | RP11-223P11.3 | hsa-miR-29a-3p | lncRNA |
| RUNX2 | hsa-miR-302a-3p | mRNA | RP11-223P11.3 | hsa-miR-767-5p | lncRNA |
| RUNX2 | hsa-miR-1237-3p | mRNA | AC005324.6 | hsa-miR-765 | lncRNA |
| RUNX2 | hsa-miR-616-3p | mRNA | RP11-146D12.2 | hsa-miR-335-3p | lncRNA |
| NCKAP1L | hsa-miR-1322 | mRNA | RP11-638I8.1 | hsa-miR-558 | lncRNA |
| NCKAP1L | hsa-miR-194-3p | mRNA | RP11-335L23.4 | hsa-miR-335-3p | lncRNA |
| SPTBN1 | hsa-miR-143-3p | mRNA | RP11-94C24.13 | hsa-miR-30b-3p | lncRNA |
| NCKAP1L | hsa-miR-30b-3p | mRNA | RP1-29C18.10 | hsa-miR-1184 | lncRNA |
| WFS1 | hsa-miR-558 | mRNA | CTB-171A8.1 | hsa-miR-767-3p | lncRNA |
| RUNX2 | hsa-miR-1184 | mRNA | MCF2L-AS1 | hsa-miR-767-5p | lncRNA |
| RUNX2 | hsa-miR-205-5p | mRNA | CTD-2311B13.1 | hsa-miR-1184 | lncRNA |
| GAS6-AS1 | hsa-miR-765 | lncRNA | RP1-17K7.1 | hsa-miR-616-3p | lncRNA |
| RP11-326C3.10 | hsa-miR-767-5p | lncRNA | ATP2A1-AS1 | hsa-miR-765 | lncRNA |
| LL22NC03-2H8.5 | hsa-miR-1322 | lncRNA | RP11-407A16.4 | hsa-miR-1237-3p | lncRNA |
| C10orf91 | hsa-miR-30b-3p | lncRNA | RP11-244B22.11 | hsa-miR-30b-3p | lncRNA |
| RP11-326C3.14 | hsa-miR-767-5p | lncRNA | LINC01122 | hsa-miR-335-3p | lncRNA |
| RP11-138B4.1 | hsa-miR-765 | lncRNA | AC139099.4 | hsa-miR-1184 | lncRNA |
| TMEM191C | hsa-miR-1184 | lncRNA | RP11-384K6.6 | hsa-miR-214-5p | lncRNA |
| GS1-519E5.1 | hsa-miR-1237-3p | lncRNA | CTD-2562J17.2 | hsa-miR-1229-3p | lncRNA |
| RP11-627G23.1 | hsa-miR-765 | lncRNA | RP11-96K19.4 | hsa-miR-335-3p | lncRNA |
| LINC00689 | hsa-miR-1184 | lncRNA | CCDC144NL-AS1 | hsa-miR-767-3p | lncRNA |
| MUC2 | hsa-miR-30b-3p | lncRNA | COL18A1-AS1 | hsa-miR-1184 | lncRNA |
| RP11-458F8.4 | hsa-miR-558 | lncRNA | RP3-402G11.28 | hsa-miR-1184 | lncRNA |
| RP3-470B24.5 | hsa-miR-1184 | lncRNA | AC012501.2 | hsa-miR-1229-3p | lncRNA |
| SLC8A1-AS1 | hsa-miR-335-3p | lncRNA | RP11-480I12.10 | hsa-miR-30b-3p | lncRNA |
| RP11-384K6.6 | hsa-miR-558 | lncRNA | RP13-580B18.4 | hsa-miR-214-5p | lncRNA |
| RP4-539M6.22 | hsa-miR-302a-3p | lncRNA | RP4-539M6.22 | hsa-miR-767-5p | lncRNA |
| HCG22 | hsa-miR-1184 | lncRNA | RP11-673P17.2 | hsa-miR-767-3p | lncRNA |
| RP5-991B18.1 | hsa-miR-558 | lncRNA | CTC-548K16.6 | hsa-miR-1237-3p | lncRNA |
| RP4-671O14.7 | hsa-miR-194-3p | lncRNA | RP11-570L14.2 | hsa-miR-765 | lncRNA |
| LINC00265 | hsa-miR-558 | lncRNA | RP11-804H8.7 | hsa-miR-214-5p | lncRNA |
| HPVC1 | hsa-miR-214-5p | lncRNA | LL22NC03-2H8.5 | hsa-miR-143-3p | lncRNA |
| LL22NC03-27C5.1 | hsa-miR-1229-3p | lncRNA | AC091153.4 | hsa-miR-1237-3p | lncRNA |
| RP11-1260E13.4 | hsa-miR-1184 | lncRNA | TMEM191A | hsa-miR-1184 | lncRNA |
| MUC19 | hsa-miR-194-3p | lncRNA | AC011284.3 | hsa-miR-30b-3p | lncRNA |
| AC092535.3 | hsa-miR-1184 | lncRNA | RP5-902P8.10 | hsa-miR-767-3p | lncRNA |
| LINCMD1 | hsa-miR-30b-3p | lncRNA | RP11-90K6.1 | hsa-miR-1237-3p | lncRNA |
| RP11-153F5.7 | hsa-miR-30b-3p | lncRNA | ST20-AS1 | hsa-miR-765 | lncRNA |
| TMEM191C | hsa-miR-767-3p | lncRNA | FAM95B1 | hsa-miR-194-3p | lncRNA |

**Abbreviations**: ceRNA: Competing endogenous RNA; lncRNA: Long non-coding RNA; mRNA: Messenger RNA; miRNA: MicroRNA.
